# Protocols for cognitive enhancement. A user manual for Brain Health Services—part 5 of 6

**DOI:** 10.1186/s13195-021-00844-1

**Published:** 2021-10-11

**Authors:** Andrea Brioschi Guevara, Melanie Bieler, Daniele Altomare, Marcelo Berthier, Chantal Csajka, Sophie Dautricourt, Jean-François Démonet, Alessandra Dodich, Giovanni B. Frisoni, Carlo Miniussi, José Luis Molinuevo, Federica Ribaldi, Philip Scheltens, Gael Chételat

**Affiliations:** 1 Centre Leenaards de la Mémoire, Centre Hospitalier Universitaire Vaudois, Lausanne, Switzerland; 2 Laboratory of Neuroimaging of Aging (LANVIE), University of Geneva, Geneva, Switzerland; 3 Memory Clinic, Geneva University Hospitals, Geneva, Switzerland; 4 Unit of Cognitive Neurology and Aphasia, Centro de Investigaciones Médico-Sanitarias, University of Malaga, Malaga, Spain; 5 Instituto de Investigación Biomédica de Málaga – IBIMA, Malaga, Spain; 6 Center for Research and Innovation in clinical Pharmaceutical Sciences, University Hospital and University of Lausanne, Lausanne, Switzerland; 7 School of Pharmaceutical Sciences, University of Geneva, Geneva, Switzerland; 8 Institute of Pharmaceutical Sciences of Western Switzerland, University of Geneva, University of Lausanne, Geneva, Switzerland; 9 Normandie Univ, UNICAEN, INSERM, U1237, PhIND “Physiopathology and Imaging of Neurological Disorders”, Institut Blood and Brain @ Caen-Normandie, Cyceron, 14000 Caen, France; 10 Center for Mind/Brain Sciences – CIMeC, University of Trento, Rovereto, Italy; 11 Barcelonaβeta Brain Research Center, Pasqual Maragall Foundation, Barcelona, Spain; 12 Laboratory of Alzheimer’s Neuroimaging and Epidemiology (LANE), Saint John of God Clinical Research Centre, Brescia, Italy; 13 Department of Molecular and Translational Medicine, University of Brescia, Brescia, Italy; 14 Alzheimer Center Amsterdam, Department of Neurology, Amsterdam Neuroscience, Vrije Universiteit Amsterdam, Amsterdam UMC, Amsterdam, The Netherlands

**Keywords:** Subjective cognitive decline, Cognitive enhancement, Cognitive intervention, Mindfulness meditation, Physical training, Non-invasive brain stimulation, Drugs, Brain Health Service

## Abstract

Cognitive complaints in the absence of objective cognitive impairment, observed in patients with subjective cognitive decline (SCD), are common in old age. The first step to postpone cognitive decline is to use techniques known to improve cognition, i.e., cognitive enhancement techniques.

We aimed to provide clinical recommendations to improve cognitive performance in cognitively unimpaired individuals, by using cognitive, mental, or physical training (CMPT), non-invasive brain stimulations (NIBS), drugs, or nutrients. We made a systematic review of CMPT studies based on the GRADE method rating the strength of evidence.

CMPT have clinically relevant effects on cognitive and non-cognitive outcomes. The quality of evidence supporting the improvement of outcomes following a CMPT was high for metamemory; moderate for executive functions, attention, global cognition, and generalization in daily life; and low for objective memory, subjective memory, motivation, mood, and quality of life, as well as a transfer to other cognitive functions. Regarding specific interventions, CMPT based on repeated practice (e.g., video games or mindfulness, but not physical training) improved attention and executive functions significantly, while CMPT based on strategic learning significantly improved objective memory.

We found encouraging evidence supporting the potential effect of NIBS in improving memory performance, and reducing the perception of self-perceived memory decline in SCD. Yet, the high heterogeneity of stimulation protocols in the different studies prevent the issuing of clear-cut recommendations for implementation in a clinical setting. No conclusive argument was found to recommend any of the main pharmacological cognitive enhancement drugs (“smart drugs”, acetylcholinesterase inhibitors, memantine, antidepressant) or herbal extracts (*Panax ginseng*, *Gingko biloba*, and *Bacopa monnieri)* in people without cognitive impairment.

Altogether, this systematic review provides evidence for CMPT to improve cognition, encouraging results for NIBS although more studies are needed, while it does not support the use of drugs or nutrients.

**Supplementary Information:**

The online version contains supplementary material available at 10.1186/s13195-021-00844-1.

## Background

Forgetfulness is one of the most common worries among the elderly. While in some cases, subjects are satisfied with their cognitive functions and simply concerned about preserving them (worried-well, WW), others perceive a subjective decline in cognition in the absence of objective evidence of cognitive impairment (subjective cognitive decline, SCD). Although not described in DSM-V or ICD-11, the detection of SCD in clinical practice and the knowledge that biomarkers of neurodegenerative disorders appear long before the onset of objective cognitive deficits was a motivation for the SCD-Initiative working group to establish research criteria [1], recently commented and completed by Jessen et al. (2020) [2].

Representing a high percentage of patients seeking help in memory clinics for whom specific instructions are lacking [3], the definition of interventions to reduce the risk of cognitive decline and dementia in these subjects is a clinical need that is unmet. Up to 40 % of dementia cases could in fact be prevented by acting on modifiable factors (e.g., cardiovascular factors, depression, physical inactivity, social isolation, education) [4], thus interventions should target cognitively unimpaired individuals [5], especially those who have SCD. In order to address this need, we envision the creation of Brain Health Services, i.e. new services with specific missions, namely dementia risk profiling [6], dementia risk communication [7], dementia risk reduction [8], and cognitive enhancement [9], and with specific societal challenges [10].

This review focuses on randomized control trials (RCT) assessing techniques expected to improve cognition, thus targeting interventions that generally improve the performance in a short-term period (weeks, months), including cognitive, mental, or physical training (CMPT), non-invasive brain stimulations (NIBS), drugs, and nutrients.

The goal is to make “actionable” clinical recommendations based, whenever possible, on the Grading of recommendations assessment, development, and evaluation (GRADE) methodology.

### Cognitive, mental, or physical training (CMPT)

Here we considered as a CMPT intervention any training that had a potential impact on cognition, including cognitive intervention, physical activity and mental training e.g., mindfulness meditation.

Two recent papers, a systematic review and a meta-analysis, addressed the topic of cognitive enhancement with various interventions on the SCD population [11, 12]. Both of them found encouraging results in favor of a positive effect, not only on cognition, but also on well-being and quality of life. Smart et al. (2017) reviewed 9 studies (mainly RCT) addressing the effect of various non-pharmacological interventions on SCD older than 55 years [11]. Despite a large heterogeneity of designs and study quality, the interventions had a positive impact on the outcomes, with a small global effect size (effect size = 0.22, highest density intervals (HDI) = 0.01 to 0.51), which increased when taking into consideration only cognitive interventions (including mindfulness meditation) (effect size = 0.37, HDI: 0.06 to 0.71). Bhome et al. (2018) included 20 studies with both non-pharmacologic and pharmacologic interventions [12]. Cognitive training improved slightly, but significantly, objective cognitive performance. In contrast, psychological interventions (e.g., psycho-education, mindfulness meditation) significantly improved well-being but failed to improve metacognitive abilities or other cognitive performances.

#### Cognitive interventions and physical training

Cognitive intervention is a powerful mean to stimulate brain plasticity, as it showed not only an impact on behavior but also on the brain [13–15]. There are two main kinds of cognitive interventions: restorative (repeated practice) and compensation programs (strategic learning) (see Table 2); they both imply to train a specific cognitive function. However, a restorative program targets a dysfunctional cognitive function and aims to improve it with repeated practice. A compensatory program aims at supporting the impaired function, relying on unimpaired functions, and using strategies or metacognitive skills to compensate via alternative pathways [16].

Physical training intervention is a structured and repetitive program of physical exercise among which aerobic is usually an important part. It can be associated with some cognitive training or not. Studies showed that exercise leads to an increase in brain tissue, notably in the hippocampus, and an increased level of brain-derived neurotrophic factor [17].

#### Mindfulness meditation

Meditation refers to a set of emotional and attentional regulatory training exercises [18, 19], encompassing different practices, such as focused attention, open monitoring, and loving-kindness meditations [19]. Several mindfulness-based therapy programs have been developed for health care, the first one being the mindfulness-Based Stress Reduction program by Dr. Jon Kabat-Zinn [20]. Meditation-based intervention programs usually combine weekly sessions with an instructor and daily home practice, sometimes associated with one day of more intense practice. A typical meditation practice session would consist in sitting down in quiet environment and bringing your attention on your breath, without effort, gently refocusing on your breath each time your mind wanders, without judgment. Each session can combine different types of meditative practice, which relate to different targets, such as increasing skills in regulation of attention, skills in meta-cognition, and skills in compassion and loving-kindness [19, 21]. Most of the studies currently rely on 8-weeks mindfulness-based intervention, while longer interventions have recently been developed [21, 22].

### Non-invasive brain stimulation

**Fig. 1:**
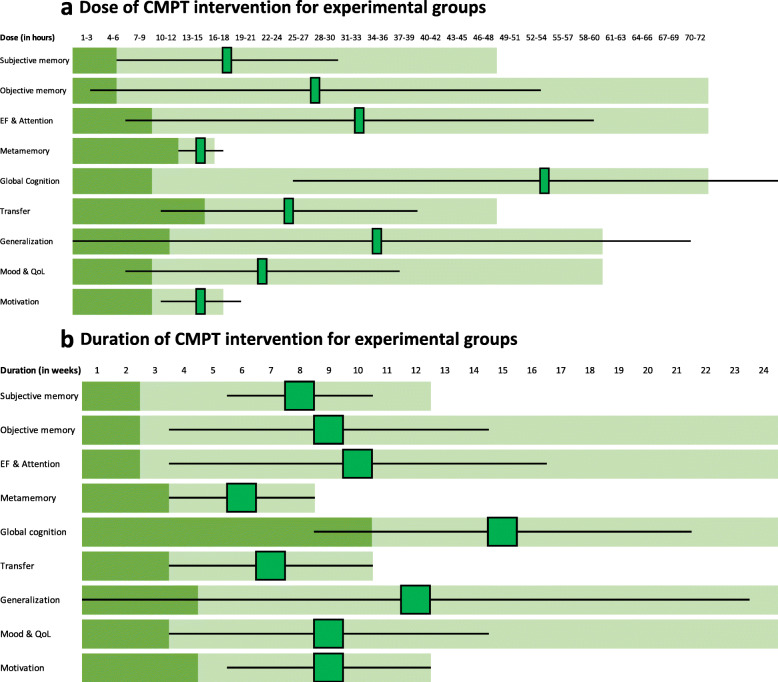
NIBS methods. **a** TMS. **b** tDCS. **a** TMS is able to generate a brief electric field in the targeted brain surface that causes a rapid depolarization of neurons above threshold. The repeated application of TMS (rTMS) induces effects that are defined as neuromodulation: low-frequency rTMS (< 1 Hz) mainly induces a reduction in the excitability, while high-frequency rTMS (between 5 and 25 Hz) induces facilitating effects in terms of excitability of the stimulated area (see [24]). **b** tDCS involves the application of weak electrical currents directly to the scalp, through a pair of electrodes, for a few minutes (~ 5–20). These currents generate an electric field that modulates neuronal activity. Several studies showed that anodal tDCS increases the frequency of neurons spontaneous discharge in the stimulated area, while cathodal tDCS has the opposite effect (see [25, 26])

Non-invasive brain stimulation (NIBS) includes different methods aimed at inducing transient changes in brain activity and consequent variations in behavioral responses. Among different NIBS techniques, the most used are repetitive transcranial magnetic stimulation (rTMS) and low intensity transcranial direct current stimulation (tDCS). Even if these two methods influence neuronal states through different means (see Fig. 1), they both imply, as an essential element, the induction of a modulation of the neural activity. The basic mechanism is the enhancement or inhibition of synaptic transmission, which can lead to changes in activity in specific cortical areas, and changes in functional connectivity between brain regions [23].

### Drugs

The aging process decreases cerebral blood flow and synaptic plasticity potentially leading to atrophy and loss of function [27]. Since aging is also accompanied by neurotransmitter dysfunction [1], there is a justification for evaluating the safety and efficacy of cognitive-enhancing drugs (CED or *smart drugs*) in individuals with SCD as well as in cognitively unimpaired older subjects. The aim of such a therapeutic approach is leveraging neurotransmitter activity to compensate for subtle aging-associated cognitive and behavioral changes [28–30].

## Methods

### Search strategy and selection criteria

A systematic approach has been used to review CMPT interventions (see Figure S1 and S2 in Supplementary Material). We considered CMPT intervention with no term restrictions in our systematic search. Those interventions were either unique or combined, with a high heterogeneity in designs (Table 1). We grouped those interventions in repeated practice (including mindfulness meditation, training on attention, executive functions, or memory), strategic learning (including psycho-education, learning of cognitive strategies), or physical training to help our understanding of their impact on our outcomes and to stay statistically rigorous (for grouping details and definition see Table 2).

**Table 1:** Experimental design of the selected studies

Author	Year	Refs	Nb of groups	Interv. group	Active Ctrl	Passive Ctrl
Cheng	2018	[31]	2	1	1	0
Innes	2018	[32]	2	1	1	0
Kwok	2013	[33]	2	1	1	0
Oh	2018	[34]	3	1	1	1
Pereira-Morales	2018	[35]	3	2	1	0
Small	2006	[36]	2	1	0	1
Smart	2016	[37]	2	1	1	0
Barnes	2013	[38]	4	3	1	0
Boa Sorte Silva	2018	[39]	2	1	1	0
Fabre	1999	[40]	4	3	0	1
Lautenschlager	2008	[41]	2	1	1	0
Andrewes	1996	[42]	2	1	1	0
Cohen-Mansfield	2015	[43]	3	1	2	0
Fairchild & Scogin	2010	[44]	2	1	0	1
Frankenmolen	2018	[45]	2	1	1	0
Hoogenhout	2012	[46]	2	1	0	1
McEwen	2018	[47]	2	1	1	0
Pike	2018	[48]	3	2	1	0
Scogin	1985	[49]	2	1	0	1
Valentijn	2005	[50]	3	2	0	1
van Hooren	2007	[51]	2	1	0	1
Youn	2011	[52]	2	1	0	1

**Table 2:** CMPT interventions

	Main intervention type	Objective(s)
Cognitive training	Repeated practice (RP)	To train a specific cognitive function, such as attention, by repeating a set of actions numerous times (e.g., in a video game or in mindfulness) to improve its performances (speed processing, decreasing the rate of errors for video game, or staying focus on breath and body sensations for mindfulness). It is often referred to as a restorative approach in patients' studies.
	Strategic learning (SL)	To optimize daily living functioning by learning strategies to optimally memorize new information, or by learning new methods to organize objects at home. It often contains psychoeducation and is referred to as compensatory approach in patients’ studies.
Physical training (PT)	Program of structured physical exercises	To practice sustained physical activity with a program that usually contains: warm up, aerobic exercises (e.g., running), +/− resistance training, and cool down exercises (stretching/relaxation). Aerobics, in particular, is known to lead to a high pulse rate of approximately 80% of one’s O_2_ maximal rate, which has a positive effect on brain tissue. It can be linked to cognitive intervention or not.
Example of active control interventions		
Passive programs	Watching videos or listening to music.	
Health program	To provide knowledge and advises on health factors linked to aging (cardiovascular disease prevention for example).	
Stretching program	To reinforce strength and, balance as well as relaxation.	

Briefly, we identified two streams of research, first using previous systematic reviews and, second, completing the review with recent works. Only two systematic reviews on SCD used a clear conceptual framework that was described by Jessen in 2014 [11, 12].

From the 29 studies involved in both reviews, we excluded 12 of them (see Figure S2 for details on selection). Regarding the research of more recent studies (October 2017- June 2020), we used similar but less restrictive terms than Bhome et al.’s (2018) since we included any kind of intervention. Altogether, our GRADE analysis was thus conducted on 22 articles, 17 from preview systematic reviews, and 5 recent publications (see Figure S1 for queries details and Figure S2 for details on selection).

As for CMPT interventions, the same literature review approach has been used for NIBS and drugs. However, literature findings for these techniques in SCD populations were very limited (i.e., 3 papers for NIBS and none for drugs, see results section for details). Therefore, in these cases, no GRADE analysis has been performed.

### GRADE and outcome measures

GRADE analysis aims to develop guidelines for clinicians based on a structured and transparent methodology for the rating of the quality of evidence [53].

GRADE analysis was implemented by two experienced neuropsychologists, following the methodology described in Guyatt et al. [53] and on “Gradepro.com” website. The quality of evidence was judged on several domains: risk of bias, inconsistency, indirectness, imprecision, and publication bias. We based our judgment for the risk of bias on allocation concealment, blinding, free of selective reporting, and mean intention to treat, as described in Guyatt et al. [53]. See Figure S3 in Supplementary Material for more details.

To select our outcomes, we identified any actionable domain that could be addressed by an intervention with a potential effect on people’s lives in our target population (i.e., SCD subjects). We chose cognitive domains that are relevant in pre-dementia syndromes and regarding intervention method (subjective and objective memory, metamemory, executive functions, attention, and global cognition), proximal and distal transfer, as well as generalization of the improvement on daily life activities. Moreover, we selected three non-cognitive domains for their impact on intervention success and/or on cognitive decline: motivation, mood, and quality of life.

### Statistics

To capture more information on the impact of specific interventions on the outcomes of interest, we completed the systematic review and GRADE analyses with additional statistics when the outcomes were addressed by more than five studies. Due to the abnormal distribution of most of our data and the use of categorical variables (efficacy: yes/no, intervention types), we carried out non-parametric analyses.

Using Fisher’s exact test, we analyzed each outcome of interest for the relationship between interventions and efficacy.

To understand whether the treatment’s dose (intervention’s total number of hours) and duration (number of weeks that the intervention lasted) are correlated with the efficacy of the treatment (dose-response, duration-response relationships), we ran Kruskal-Wallis analyses.

Analyses were performed using IBM SPSS Statistics 26 (SPSS-Inc., Chicago), with *p* < 0.05 as the significance level.

## Results

### Cognitive, mental, and physical training

#### Effect of interventions on a specific cognitive function (subjective and objective memory, metamemory, executive function/attention)

This review found 12 RCT studies that addressed subjective memory as an outcome [31, 32, 34–37, 40, 43–45, 48, 51] and 18 RCT studies that treated objective memory [31, 33–36, 38–47, 49, 50, 52]. The quality of evidence across studies for both outcomes was low (see Table 3).

**Table 3:**
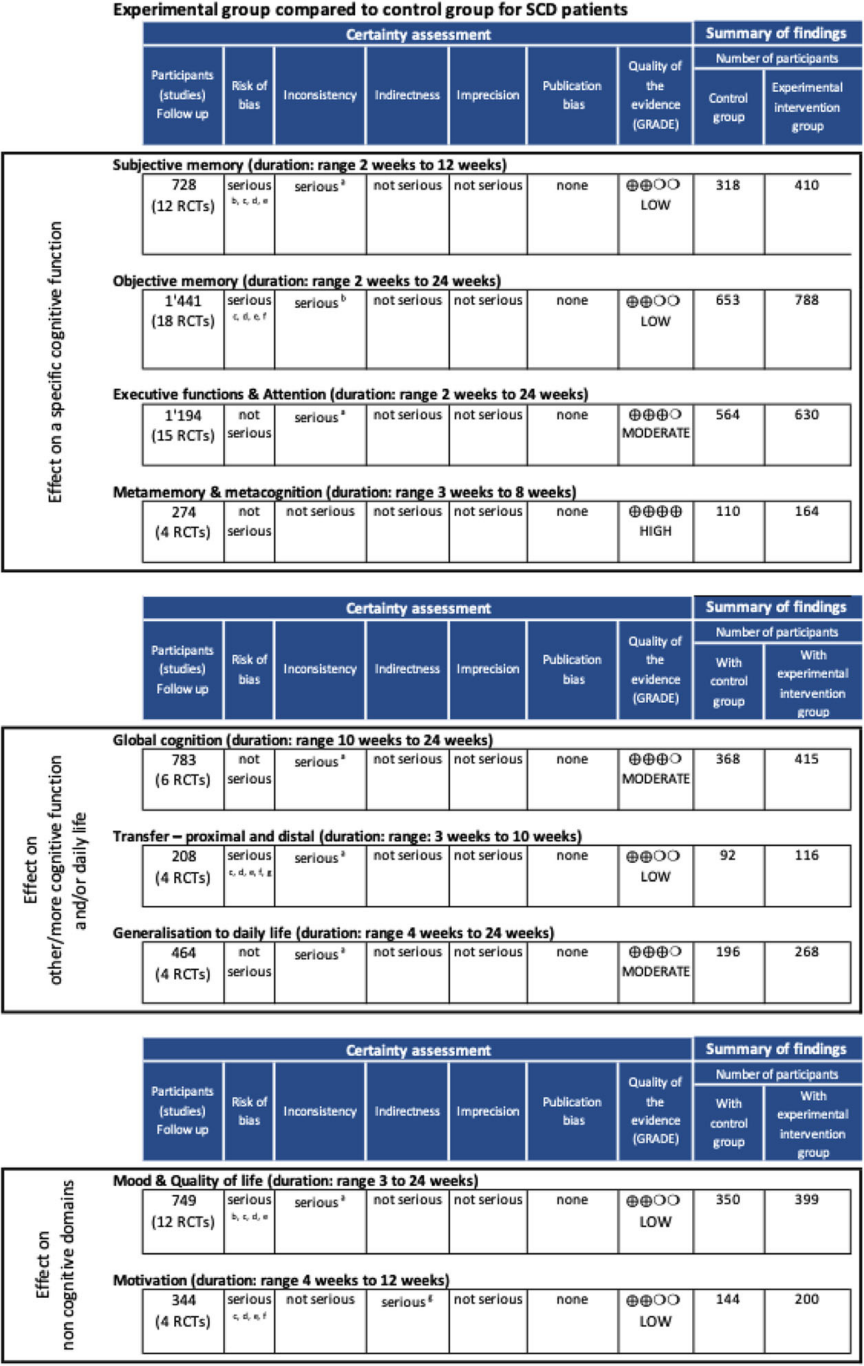
GRADE’s overall quality of evidence in SCD population engaged in CMPT

Legend: Actionable domains were identified and relevant outcomes for the SCD population were selected and classified in three sub-categories: (i) direct effects on a specific cognitive function, (ii) effects on global cognition and/or daily life, and (iii) effects on non-cognitive domains

GRADE Working Group grades of evidence. High quality: Further research is very unlikely to change our confidence in the estimate of effect. Moderate quality: Further research is likely to have an important impact on our confidence in the estimate of effect and may change the estimate. Low quality: Further research is very likely to have an important impact on our confidence in the estimate of effect and is likely to change the estimate. Very low quality: We are very uncertain about the estimate

^a^Results are very different depending on the study. ^b^Usually, studies show a positive impact, but sometimes it is not higher than other therapies. In 5 studies, there was no significant positive objective memory evolution. In 4 studies, there was a positive and significant improvement of objective memory but not significantly higher than in the other therapies. ^c^Few blinded studies. ^d^The inclusion criteria for SCD is not good enough, a major problem even in recent studies. ^e^Very often no mean intention to treat analyses. ^f^Allocation for treatment is always respected (RCT) and data are well reported. ^g^Use of other variables (attendance to a group, exercises’ done...)

Fifteen RCT studies addressed executive functions/attention as an outcome and the overall quality of evidence was moderate (Table 3) [31–39, 41, 43, 46, 47, 51, 52]. Qualitatively though, it is interesting to note that the inconsistency of results applies to all intervention types except for repeated practice: six repeated practice interventions over eight, improved executive functions and attention, including one of mindfulness meditation (Table 4) [31, 33–37].

**Table 4:**
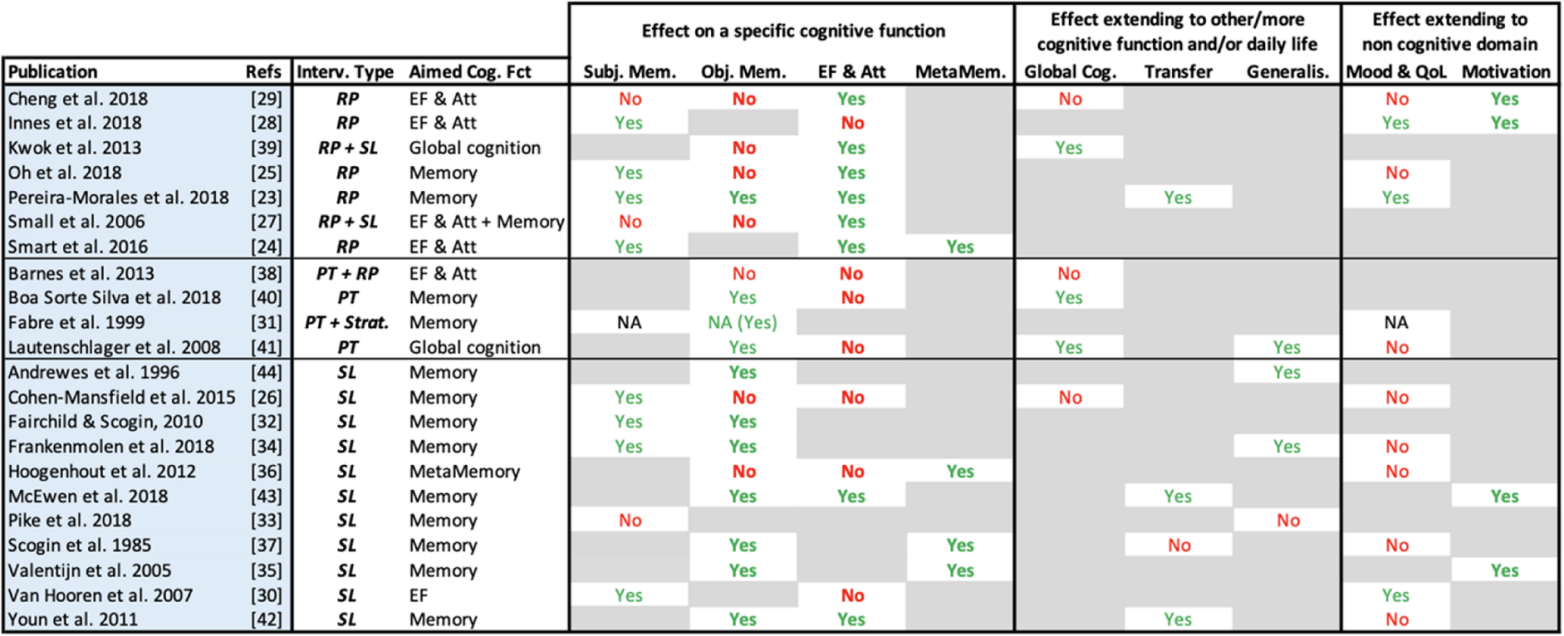
Efficacy of CMPT experimental interventions

Legend: The majority of these studies used a Time x Intervention design to check whether there was a differential effect on the studied outcome-dependent variable (objective memory for instance). This table summarizes the effects found by each study for all outcomes of interest (dependent variables): “Yes” corresponds to a significant effect on that outcome; “No” means that the interaction was not significant; “NA” was used when the design was not “Time x Intervention”; white cells represent the outcomes targeted by each study, whereas gray cells are outcomes not addressed within a study

Abbreviations: *Interv.* intervention, *Cog* Fct: cognitive function, *RP* repeated practice, *SL* strategic learning, *PT* physical training; *Subj. Mem.* subjective memory, *Obj. Mem.* objective memory, *EF & Att* executive functions and attention, *MetaMem.* metamemory, *Cog.* cognition, *Generalis.* generalization to daily life, *QoL* quality of life

Metamemory outcome was addressed in only 4 studies, [37, 46, 49, 50] which showed the high quality of evidence (Table 3). Compared to control groups, all studies found a significant improvement in metamemory after the intervention (repeated practice—more specifically mindfulness meditation, and strategic learning, alone or combined to psychoeducation) (Table 4).

Looking thoroughly at the efficacy of interventions on cognition, this review showed that the type of intervention was generally not associated with the efficacy of the interventions on these outcomes, except for executive function and attention (Table 5). There is a significant association between the type of intervention and whether or not the participants improved on executive functions/attention tasks. Moreover, there was no significant association between the type of intervention and objective memory. Interestingly, if we compared the two main types of interventions, repeated practice, and strategic learning, there was a significant difference, with an improvement of objective memory after a strategic learning intervention, but not after repeated practice (Table 5 and S1a). Qualitatively, both studies assessing mindfulness meditation found a significant improvement in subjective memory, [32, 37] whereas both studies with physical training as a unique intervention significantly improved objective memory [39, 41].

**Table 5:**
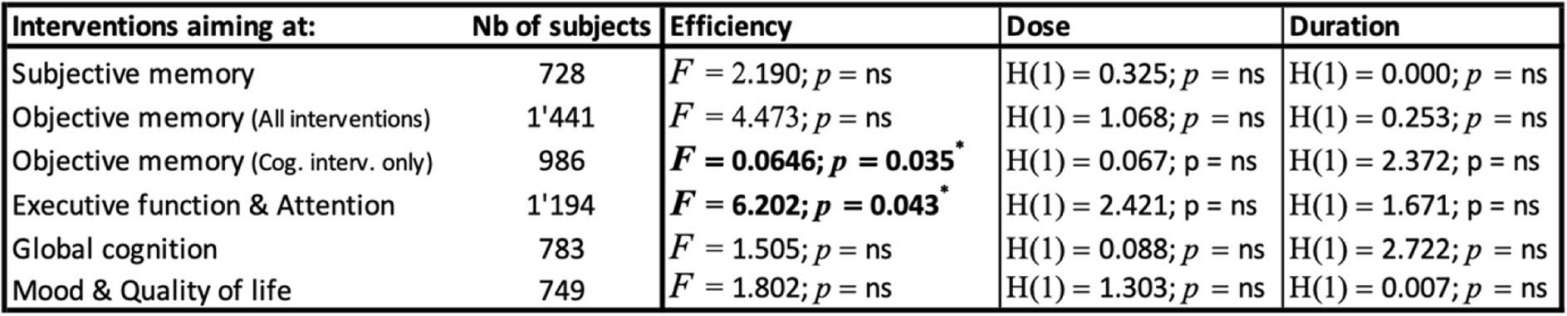
Statistics for outcomes encompassing 5 CMPT studies

Legend: Fisher’s exact tests (*F*) (2-sided) are used to check for efficacy. Kruskal-Wallis tests (*H* (degree of freedom)) are used to investigate whether dose or duration have an impact on intervention outcome. References of the studies assessing subjective memory [31, 32, 34–37, 43–45, 48, 51], objective memory (all) [31, 33–36, 38–47, 49, 50, 52], objective memory (cognitive only) [31, 33–36, 42–47, 49, 50, 52], executive function and attention [31–39, 41, 43, 46, 47, 51, 52], global cognition [31, 33, 38, 39, 41, 43], and mood and quality of life [31, 32, 34, 35, 38, 40, 41, 43, 45, 46, 49, 51, 52]

**Fig. 2:**
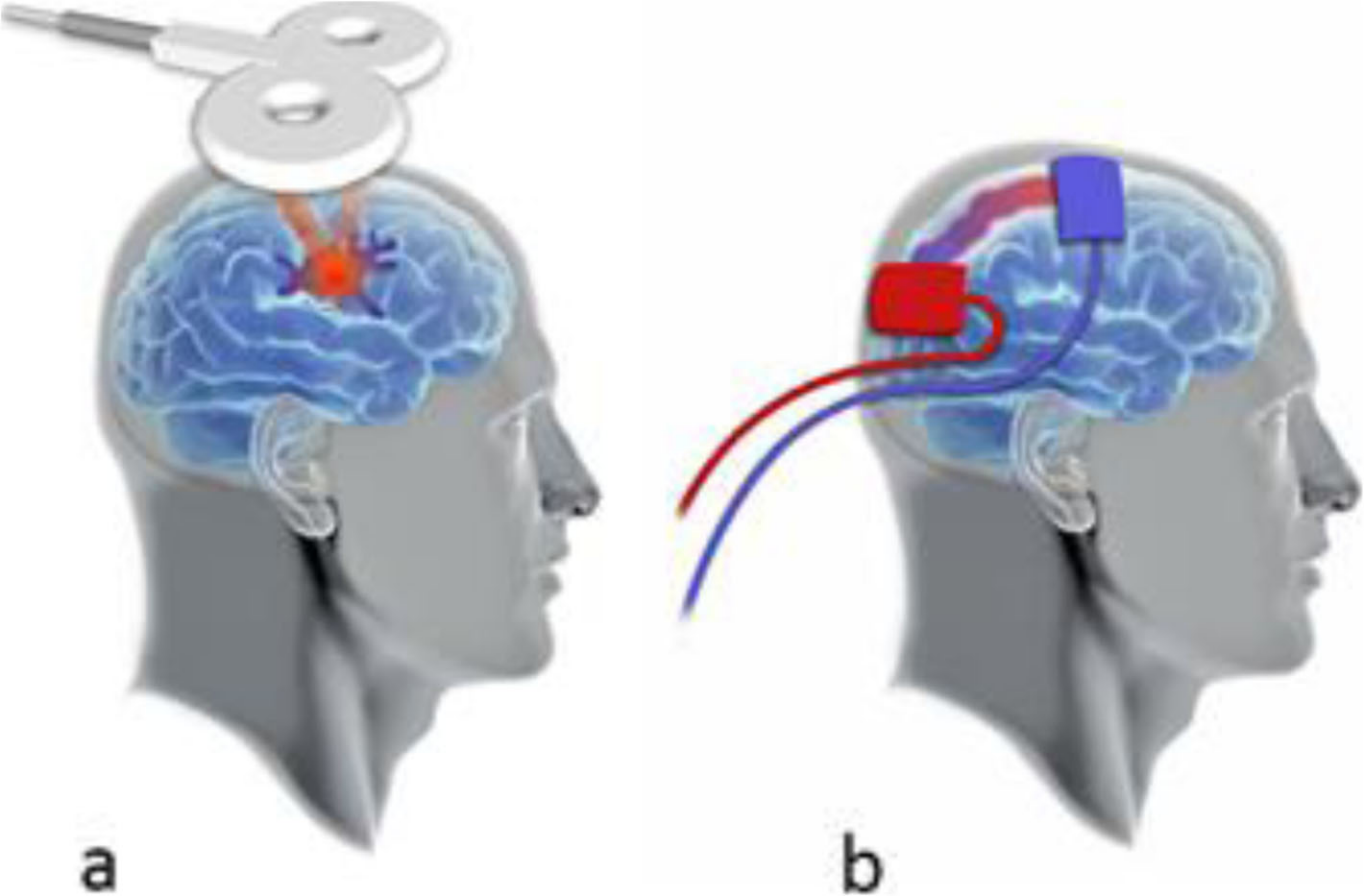
**a** Dose of CMPT intervention for experimental groups. **b** Duration of CMPT intervention for experimental groups. Legend: **a** Minimum (dark green) and maximum (light green) experimental interventions’ dose for each elicited GRADE outcome. Squares indicate the mean dose and mustaches the standard deviation. **b** Idem for duration

Across studies that address these outcomes, there was no association between efficacy of the intervention types and dose or duration of interventions (Table 5, see also Fig. 2a and b for mean dose and duration per outcome).

#### Effect extending to other/more cognitive functions and daily life (global cognition, transfer, and generalization)

We analyzed 8 interventions across 6 RCT studies that addressed global cognition, with a moderate quality of evidence (Table 3) [31, 33, 38, 39, 41, 43]. Moreover, we found 4 RCT studies addressing proximal or distal transfer as an outcome, with a low quality of evidence across studies, [35, 47, 49, 52] and 4 RCT studies addressing generalization of the improvement in daily life, with a moderate quality of evidence (Table 3) [41, 42, 45, 48].

Regarding global cognition, the efficacy was not associated to intervention type, dose or duration (Table 5 and suppl1b). Qualitatively, most of the studies assessing transfer and generalization showed a significant impact of the intervention (3/4 for both outcomes). Also, both studies assessing physical training found a significant improvement in global cognition [39, 41].

#### Effect on non-cognitive domains (mood, quality of life, motivation)

Twelve RCT studies addressed mood or quality of life as an outcome [31, 32, 34, 35, 40, 43, 45–47, 49, 51, 52] while 4 studies addressed motivation as an outcome [31, 32, 47, 50] and the quality of evidence across these studies was low (Table 3).

Intervention type analysis failed to demonstrate correlations between interventions and their efficacy on mood/quality of life. Only three studies found an improvement, including one assessing mindfulness meditation [32]. Additionally, efficacy on these outcomes was not correlated with dose or duration of the intervention (Table 5 and S1c).

Due to the small number of studies addressing motivation as an outcome, we did not process any statistical analysis for efficacy, dose, or duration; nevertheless, it is interesting to note that all studies measuring motivation found a positive result (Table 4).

### Non-invasive brain stimulation

A high number of investigations indicate that interacting with brain activity by means of NIBS can positively affect cognitive performance in patients in the Alzheimer disease continuum, possibly reducing the impact of progressive symptomatic decline [54, 55]. On the other hand, the role of NIBS in maintaining cognitive performance at preclinical stages and in healthy elderly people remains to be confirmed.

The literature research yielded only three original articles [56–58], which were characterized by a high heterogeneity in the study design and in SCD inclusion criteria (for details see Table S2). Overall, even if preliminary, these results showed encouraging evidence on the potential effect of NIBS in reducing memory concern s[59] and in improving long-lasting episodic memory (see Table S2) [60, 61]. Despite the lack of evidence on SCD, literature generated over the last years suggests NIBS as a promising technique to maintain cognitive functioning in the aging population; thus, in the next paragraphs, we will provide an overview about the evidence on multi-session interventions, as they can provide the most relevant insights on the NIBS therapeutic effects in improving or maintaining cognitive health (summarized in Table 4). While some of these studies showed a lack of benefit after multiple NIBS sessions [62–66], the majority showed positive effects in improving episodic [56, 67, 68] and working [57, 58, 69–72] memory in older adults, in some cases with long-lasting effects [57, 58, 69] and associated with significant changes at neural level [67, 72]. Across the available literature, the prefrontal cortex represented the most common stimulation target, [57, 58, 65, 66, 68–73] followed by other frontal [56] and temporo-parietal regions [58, 62, 63, 67, 70].

### Drugs

Studies with acetylcholinesterase inhibitors and memantine yielded mixed results in healthy older subjects ranging from improvement to no changes or even worsened cognitive performance [30]. Single dose and multiple doses studies with stimulants (modafinil and methylphenidate) [74] and drugs acting on dopamine (levodopa, tolcapone, pramipexole) [29] also provided mixed results. The role of old and new antidepressants has mostly been tested in late-life depression (LLD), which occurs in about 30% of the elderly population and is associated with cognitive impairment [75]. However, the extant evidence supporting such a strategy is limited, inconclusive, and difficult to translate to clinical practice. Currently, there is no evidence of a positive effect of cognitive enhancement drugs on the SCD population as most studies involved healthy young individuals or the psychiatric populations, mainly using single doses with no long-term treatment response evaluated.

Herbal extracts, in particular *Panax ginseng*, *Gingko biloba*, and *Bacopa monnieri*, occupy a prominent position in the bestseller list of drugs administered to combat aging. Although used over centuries for cognitive improvement and other indications, there is a complete lack of evidence of the benefit of these herbal products in SCD individuals. Two old small studies on *Bacopa monnieri* in individuals with memory complaints suggest a potential effect on some aspect of memory function or on attention tests that still need to be confirmed [76, 77]. In healthy subjects, *Panax ginseng* showed some evidence of improvement in some aspects of working memory and reaction times [78], but the poor strength of evidence and unreproducible results limit the ability to draw any conclusions [79]. One study showed that this herbal extract might improve attention and reaction times, whereas no effect was observed on other cognitive domains [14]. However, there is no convincing evidence that *Gingko biloba* extracts have a positive effect on any aspect of memory, executive function, and attention in healthy people after acute or longer-term administration [80–82].

## Discussion

Considering that the pathology in neurodegenerative disorders starts decades before the symptoms appear, the main objective of this work was to rigorously review techniques to make an actionable clinical recommendation to enhance cognition in SCD individuals.

### Cognitive, mental, and physical training

The systematic review on CMPT targeting SCD individuals showed positive and clinically relevant findings. Based on GRADE, we found a high quality of evidence that CMPT improved metamemory. There is moderate quality of evidence that CMPT improved executive functions, attention, and global cognition. Moreover, we found moderate quality of evidence that the positive impact on outcomes is transferable to daily life functioning (generalization). Finally, we found low quality of evidence that CMPT improved objective memory, subjective memory, motivation, mood, and quality of life, as well as a transfer to other cognitive functions.

Nevertheless, the heterogeneity in study designs and in CMPT in terms of content, dose, and duration motivated further analysis. Looking thoroughly at the impact of the different interventions, we found that learning strategies were efficient to improve objective memory, whereas repeated practice improved attention and executive function skills. This is highly interesting for clinicians. Indeed, although research separates interventions, it is more appropriate to use different techniques in a clinical setting: both learning strategies and repeated practice, as well as other methods, according to the individuals’ needs (e.g., mindfulness meditation and physical training).

The effect of mindfulness-based intervention in the SCD population was addressed by only 2 RCT studies with qualitative efficacy on subjective memory and metamemory, mood, well-being and quality of life [32, 37]. Those impacts on cognition and psycho-affective factors were consistent with studies on more diverse populations (age, pathology) [83–87]. Since depression is one of the main modifiable factors of cognitive decline, mindfulness is an interesting intervention by itself or combined with other techniques. Taken together, mindfulness-based interventions are potentially efficient trainings to enhance cognitive abilities in users with SCD.

However, studies with RCT designs, larger sample sizes, longer follow-up and active and passive control groups are needed. Importantly, there is a lack of quantification and description of interventions using meditation, which could be improved using methods such as the Rehabilitation Treatment Specification Framework [21].

The fact that repeated training and strategic learning showed an improvement on outcomes that is significantly higher than physical training, does not mean that the latter has no impact on these outcomes. Both studies that imply physical training as a unique intervention showed a significant improvement on objective memory and on global cognition [39, 41].

However, some limitations must be considered. The literature research has been performed only on one database (Pubmed), and this might have limited our findings. Besides, since some papers in the current review have been published before the introduction of the Jessen criteria [88], they included SCD patients with cognitive disorders. We addressed this limitation through GRADE analysis (risk of bias, see supplementary material for details) [88].

### Non-invasive brain stimulation

The overall current evidence suggests that an intervention combining multiple sessions of NIBS and cognitive training may lead to clinically meaningful improvements in cognition and functional independence in the aging population. However, the high heterogeneity across studies in stimulation intensity, duration, and number of sessions, as well as in the cognitive outcomes, prevent comparing the study results, and to identify the parameter set with the highest efficacy potential. So far, NIBS has mainly been used with a one-size-fits-all approach. Nevertheless, starting from the idea that it induces a gradual readjustment of an intact but “functionally” reduced area due to a steady reduction in synaptic strength, every effort that aims at improving cognition must consider the level of cognitive efficacy and neural activity of the stimulated network. Therefore, NIBS potential should be exploited before the significant neuronal loss has occurred [89], with a well-characterized sample, a precise definition of the stimulation dose based on individual anatomy [90, 91]), and adopting a single-subject approach [92, 93]). In addition to this point, the role of individual features, such as demographics (e.g., [56]) and biological variables [70], in modulating NIBS efficacy is yet to be explored.

Besides, properly designed, larger, and longer trials on subjects characterized by a higher risk for dementia (e.g., APOEε4 carriers, preclinical AD, SCD according to well-defined criteria) are needed, to address unresolved issues in the use of NIBS in combination with cognitive rehabilitation to delay or prevent the symptom onset. Overall, the precise NIBS contribution should be evaluated, as an add-on, towards a precision medicine approach implementing all the aspects previously mentioned. Despite the promising results with rTMS administration, the lack of portability, usage complexity, and the cost, represents important challenges in the implementation of this technique in the Brain Health Service. In this sense, tDCS-based neuromodulation seems to have a higher potential, due to the low cost of the instrumentation, little contraindications with a good safety record, high portability, and easy-to-implement with concurrent task execution in an ecological context.

### Drugs

This review on the effect of drugs on SCD cognition and healthy individuals included the main pharmacological cognitive enhancement (CED or smart drugs, acetylcholinesterase inhibitors, Memantine, antidepressant) and herbal extracts (*Panax ginseng*, *Gingko biloba*, and *Bacopa monnieri).* Based on this review, there is no conclusive argument to recommend pharmacological cognitive enhancement or herbal extracts on SCD or worried-well individuals.

Future studies on drugs need to pay attention to interindividual variability of response, refine testing instruments to minimize ceiling effects, and incorporate neuroimaging and genetic biomarkers to optimize treatment response prediction.

The assessment of the benefit of herbal extracts in improving cognition and their risk profile—generally safe—remains challenging due to the presence of various types of preparations, dosage, duration and type of administration, multiple active components that may influence numerous neuronal, metabolic, and hormonal systems involved in neuro-behavioral processes [94]. Further, most studies suffer from poor design and heterogenous methods and provide inconsistent or even contradictory results. In addition, any effect is subtle at best and may be very sensitive to contextual and motivational factors.

## Conclusions

Recent studies on cognitive enhancement techniques in SCD population are showing encouraging results. Even though it is too early to provide recommendations on the effect of drugs and NIBS, specific dedicated CMPT seems to have a positive effect on cognition as well as on related domains and are therefore recommended. Moreover, CMPT, including mindfulness meditation, are an interesting target as they are generally harmless, inexpensive, and easy to implement on both clinical face-to-face setting and using virtual tools. Consequently, they are actionable and accessible, reducing inequality across the population.

## Supplementary Information

**Additional file 1.**

**Additional file 2.**

## Data Availability

Data sharing is not applicable to this article as no datasets were generated or analyzed during the current study.

## Abbreviations

GRADE: Grading of Recommendations Assessment, Development and Evaluation
CMPT: Cognitive, mental, and physical training
PICO: Population, Intervention, Comparison, Outcomes
RCT: Randomized clinical trial
SCD: Subjective cognitive decline
NIBS: Non-invasive brain stimulation
CED: Cognitive enhancement drugs
RCT: Randomized controlled trial
LLD: Late-life depression
tDCS: Transcranial direct current stimulation
rTMS: Repetitive transcranial magnetic stimulation
