# Supplementary material for: Protocols for cognitive enhancement. A user manual for Brain Health Services—part 5 of 6
Source: Alzheimers Res Ther. 2021 Oct 11;13:172. doi: 10.1186/s13195-021-00844-1 (PMC8507160; doi:10.1186/s13195-021-00844-1)
Supplement: Supplementary file 1 — Additional file 1. [file 13195_2021_844_MOESM1_ESM.docx]

**Protocols for cognitive enhancement in persons at risk for dementia. A user manual for Brain Health Services – Part 5 of 5**

**Supplementary material**

**Figure S1: Search queries for mental training**

**
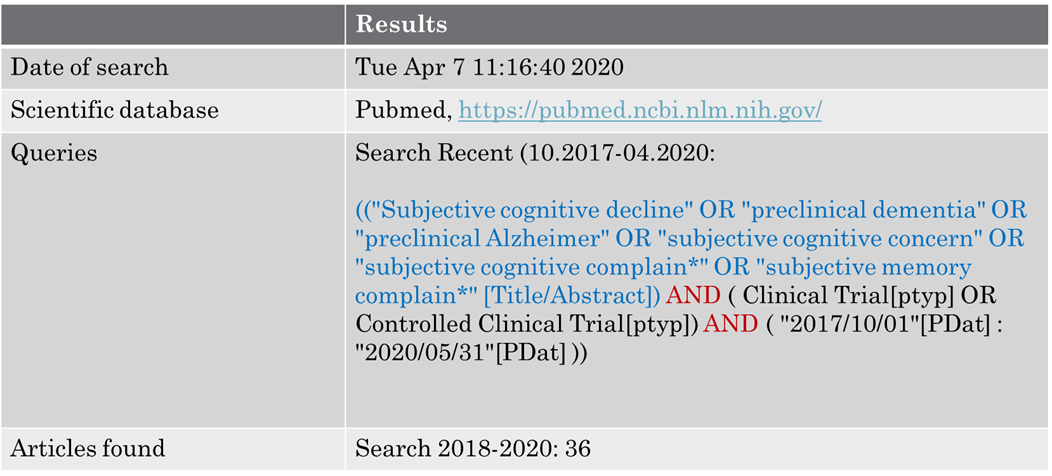
**

**Details of search strategies for mental training**

Regarding the selection process of the articles, both co-first authors (ABG, MB) read the full lot of new queries abstracts separately (36 articles) and proceeded to an inclusion/exclusion process based on the following criterion first: (a) *Inclusion criterion*: randomized controlled trials (RCTs) articles only ; (b) *Exclusion criteria*: articles dealing with drugs or nutrients, not including at least a sub-group of SCD population, dealing with young adults only (students for instance), not aiming at cognitive enhancement, with an incomplete design (not RCT, no intervention, no results or no pre/post measures).

The 2 judges agreed on the inclusion/exclusion of 26 of 36 articles, based on the abstract content. For the 10 remaining articles the two co-first authors scanned all the articles and discussed the reasons for disagreement together, agreeing to include 8 of them, excluding one article that dealt with nutrients and another one that did not present separated results for the SCD group from the MCI and dementia groups.

**Figure S2: Selection of papers**

**
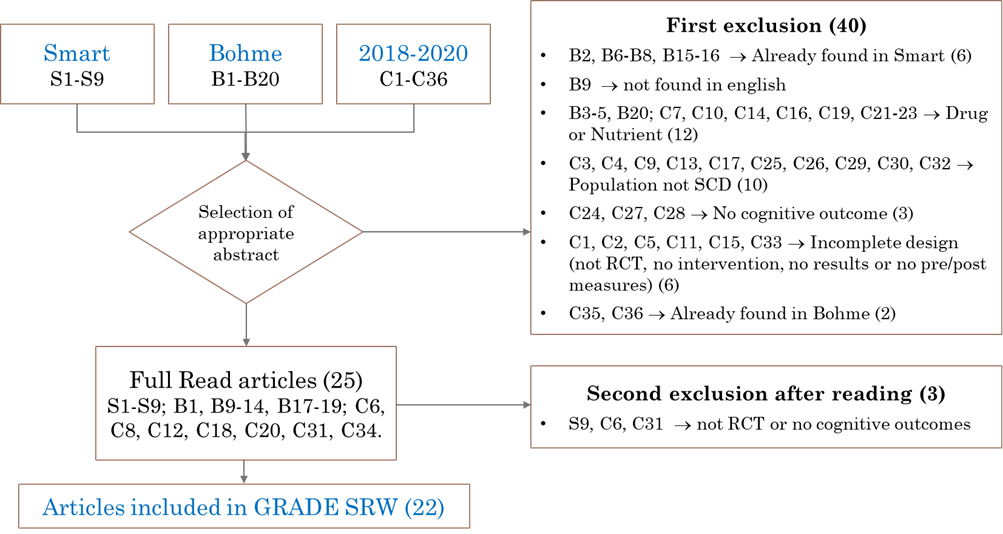
**

**Grade analysis**

GRADE analysis was implemented by two experienced neuropsychologists, following the methodology described in Guyatt and on “Gradepro.com” website.[1, 2]

To apply GRADE methodology:

1. **To raise a question on the population of interest.** The question raised in that article was: “how to enhance cognition in people with subjective cognitive decline by using non-pharmacological interventions”. As other co-authors were asked to address drugs & nutrient or NIBS, we restricted our literature research to studies focused on mental and/or physical training (including meditation as only two studies were considering this kind of mental training).
2. **To choose outcomes that are relevant for SCD’s cognition.** It can be direct outcomes (such as a specific cognitive functions or global cognition), but it can extend to any other outcomes of interest that would have a positive impact on SCD functioning in real life.
3. **To order and rate the selected outcomes by clinical importance.** To provide clinical guidelines, the selected outcomes should be hierarchically organized and rated by degree of importance: critical for decision making (rated as 7 to 9), important but not critical for decision making (rated as 4 to 6), and not relevant for decision making or of lower importance to patients (rated as 1 to 3).

Nine outcomes of interest were identified to assess the efficiency of the intervention in SCD patients. We rated subjective and objective memory, attention/executive functions and metamemory, as well as generalization to daily living functioning as having a critical importance to set clinical guidelines, while we rated the improvement in the other outcomes as important but not critical (global cognition, transfer of knowledge to other related tasks (proximal transfer) or other cognitive functions (distal transfer), mood and quality of life).

1. **To judge the quality of evidence according to a set of criteria**. The quality of evidence was judged on several domains: risk of bias, inconsistency, indirectness, imprecision and publication bias. We based our judgement for the risk of bias on allocation concealment, blinding, free of selective reporting and mean intention to treat, as described in Guyatt et al.[2] Moreover, we added a judgement on the SCD diagnosis of the population as it was not always accurate. Each criteria received a score between 1 and 3 according very clear characteristics and the judgement on the risk of bias was decided according to the mean score (1-1.5= no serious risk of bias; 1.51-2.5=serious risk of bias; 2.51-3=very serious risk of bias).

**Table S3: Risk of bias/limitation of the studies included in the GRADE analysis (green: no serious risk of bias, Yellow= serious risk of bias, Red= very serious risk of bias)**


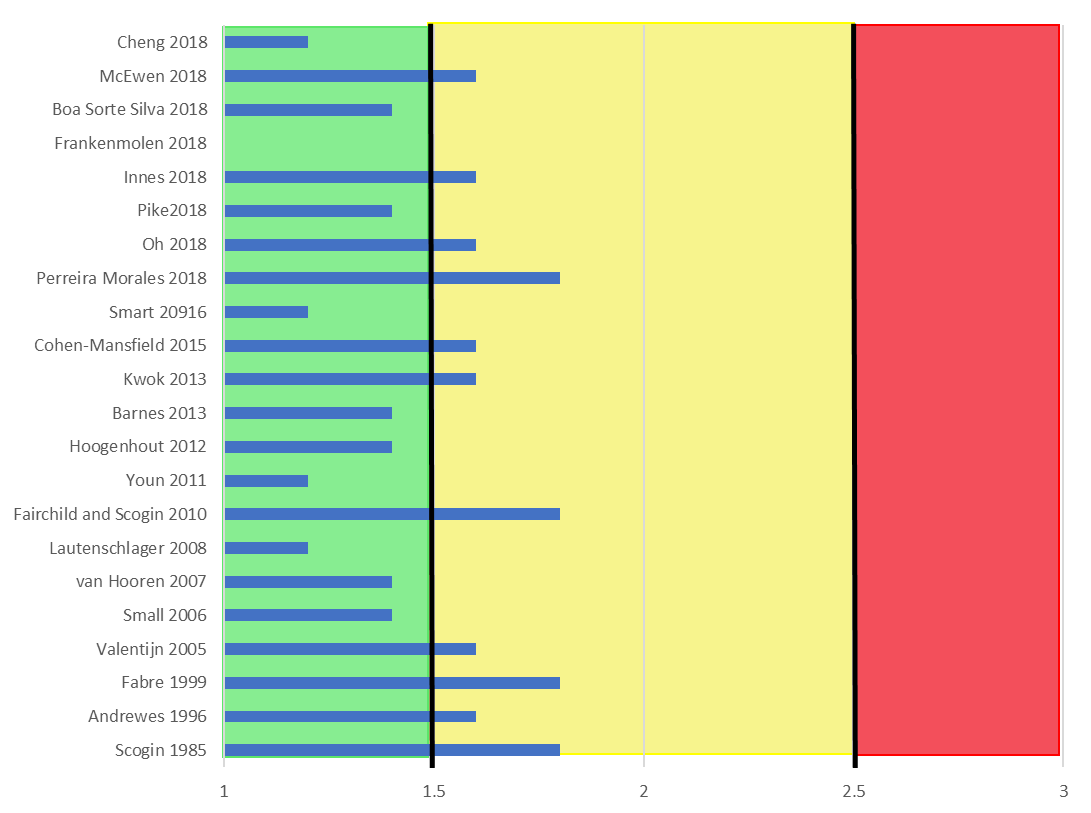


**Table S1a: Fisher test statistics for Executive functions/attention and intervention type.**

| **Cross tabulation : Intervention type * Executive functions** | | | | |
| --- | --- | --- | --- | --- |
|  | |  | | Total |
|  |  | No | Yes |  |
| IntervType1 | RP | 1 | 6 | 7 |
|  | PT | 3 | 0 | 3 |
|  | SL | 3 | 2 | 5 |
| Total | | 7 | 8 | 15 |

Legend : EF=Executive functions, RP= repeated practice, PT=physical training, SL=strategic learning. No=the intervention does not have an impact on Executive functions/attention. Yes= the intervention has an impact on Executive functions/attention

| **Chi-Square Tests** | | | | |
| --- | --- | --- | --- | --- |
|  | Value | df | Asymptotic Significance (2-sided) | Exact Sig. (2-sided) |
| Pearson Chi-Square | 6.735^a^ | 2 | .034 | .033 |
| Likelihood Ratio | 8.256 | 2 | .016 | .043 |
| Fisher's Exact Test | 6.202 |  |  | **.043** |
| N of Valid Cases | 15 |  |  |  |
| a. 6 cells (100.0%) have expected count less than 5. The minimum expected count is 1.40. | | | | |

**Table S1b: Fisher test statistics for objective memory and intervention type.**

| **Cross tabulation : Intervention Type * Objective Memory** | | | | |
| --- | --- | --- | --- | --- |
|  | |  | | Total |
|  |  | No | Yes |  |
| IntervType1 | CT | 4 | 1 | 5 |
|  | PT | 1 | 3 | 4 |
|  | Strategic | 2 | 7 | 9 |
| Total | | 7 | 11 | 18 |

Legend : EF=Executive functions, RP= repeated practice, PT=physical training, SL=strategic learning. No=the intervention does not have an impact on objective memory. Yes= the intervention has an impact on objective memory.

| **Chi-Square Tests** | | | | |
| --- | --- | --- | --- | --- |
|  | Value | df | Asymptotic Significance (2-sided) | Exact Sig. (2-sided) |
| Pearson Chi-Square | 4.932^a^ | 2 | .085 | .111 |
| Likelihood Ratio | 5.020 | 2 | .081 | .135 |
| Fisher's Exact Test | 4.473 |  |  | .111 |
| N of Valid Cases | 18 |  |  |  |
| a. 5 cells (83.3%) have expected count less than 5. The minimum expected count is 1.56. | | | | |

**Table S1c: Fisher test statistics for objective memory and intervention type, when comparing only Repeated practice and strategic learning.**

| **Cross tabulation : Intervention type * Objective Memory** | | | | |
| --- | --- | --- | --- | --- |
|  | |  | | Total |
|  |  | No | Yes |  |
| intervention | RP | 5 | 1 | 6 |
|  | SL | 2 | 8 | 10 |
| Total | | 7 | 9 | 16 |

Legend: EF=Executive functions, RP= repeated practice, PT=physical training, SL=strategic learning. No=the intervention does not have an impact on objective memory. Yes= the intervention has an impact on objective memory.

| **Chi-Square Tests** | | | | | |
| --- | --- | --- | --- | --- | --- |
|  | Value | df | Asymptotic Significance (2-sided) | Exact Sig. (2-sided) |  |
| Pearson Chi-Square | 6.112^a^ | 1 | .013 | .035 |  |
| Continuity Correction^b^ | 3.810 | 1 | .051 |  |  |
| Likelihood Ratio | 6.515 | 1 | .011 | .035 |  |
| Fisher's Exact Test | 6.460 |  |  | .**035** |  |
| N of Valid Cases | 16 |  |  |  |  |
| a. 3 cells (75.0%) have expected count less than 5. The minimum expected count is 2.63. | | | | | |
| b. Computed only for a 2x2 table | | | | | |

**Table S2.** **Description of studies using NIBS in SCD subjects.**

| **Study** | **N° subjects** | **Age (mean±SD)** | **SCD inclusion criteria** | **Intervention** | **Site** | **Protocol** | **Outcomes** | **Results** |
| --- | --- | --- | --- | --- | --- | --- | --- | --- |
| *TMS studies* | | | | | | | | |
| Solé-Padulle, 2004 | 40 | 67±9 | memory complaints (qualitative) + low performance (still in normal range) at memory tests | Offline rTMS Active (n=20)  Sham (n=20) | PFC (double-cone coil) | 1 session, 5 Hz rTMS  10 trains (10 sec) delivered in 5 min | Associative memory (ad hoc developed task) | Improvement of associative memory in active rTMS compared to sham rTMS |
| *tDCS studies* |  |  |  |  |  |  |  |  |
| Stoynova, 2019 | 26 | 69±6 | normal objective cognitive performance + memory complaints (qualitative) | Online  atDCS (n=14)  stDCS (n=12) | Anode: left PFC (F3)  Cathode: contralateral deltoid muscle | 12 sessions with CT,  20 min 2mA | Memory concern and processing speed (PASAT) | Reduction of memory concerns after atDCS-CC training and after 3-month follow-up compared to stDCS group. No effects on processing speed |
| Manenti, 2017 | 22 | 75±6 | normal objective cognitive performance +  EMQ >45.7 | Offline  atDCS (n=11)  stDCS (n=11) | Anode: left lateral PFC (F3),  Cathode: right supraorbital area | 1 session,  15 min 1.5 mA | Objective memory (free recall and recognition) | Beneﬁcial eﬀects after atDCS up to 30 days on recognition compared to stDCS group |
|  |  |  |  |  |  |  |  |  |
| *Offline: stimulation is delivered before/after/without the cognitive training. Online: stimulation is delivered during the cognitive training; CT: cognitive training;*  *SD: Standard Deviation; PASAT: Paced Auditory Serial Addition Task; EMQ: EveryDay Memory Questionnaire; rTMS: repetitive transcranial magnetic stimulation; atDCS: anodal transcranial direct current stimulation; stDCS: sham tDCS; PFC: prefrontal cortex.* | | | | | | | | |

**Table S3**. Description of studies using NIBS in healthy old subjects.

| **Study** | **N° subjects** | **Age (mean±SD)** | **Cognitive inclusion criteria** | **Intervention** | **Site** | **Protocol** | **Outcomes** | **Results** |
| --- | --- | --- | --- | --- | --- | --- | --- | --- |
| *TMS studies* | | | | | | | | |
| Beynel, 2019 | 32 | 69.7±4.8 | Dementia Rating Scale-2 score <8 | Between- subject  Online rTMS (n=16)  Sham rTMS (n=16) | rDLPFC (F4)  figure-8 coil | 4 sessions with CT, 25 pulses at 5Hz (5-sec trains) unclear n of repetitions | working memory (ad-hoc developed task) | rTMS significantly enhanced working memory manipulation abilities |
| Cui, 2020 | 32 | 67.9±5.5 | MoCA-BJ > 21,  no subjective memory complaints | Between- subject  Offline rTMS (n=16) Sham rTMS (n=16) | rDLPFC (F4)  figure-8 coil | 10 sessions, 20-min stimulation, 2000 pulses at 10 Hz per session | Episodic memory (source memory discrimination) | Source memory performance improved after rTMS compared with sham stimulation |
| Kim, 2012 | 16 | 63.13±4.9 | MMSE and CVLT within normal range | Between-subject  Offline rTMS (n=8) Sham rTMS (n=8) | lDLPFC (F3)  figure-8 coil | 5 sessions, unclear stimulation time, 780 pulses at 10 Hz per session | Inhibitory control (Stroop task) | rTMS improved performance in reaction time during incongruent trials (i.e. those with distracting information) |
| Nilakantan, 2019 | 15 | 72.5 (SD unknown), age-range 64-80 | None | Within-subject  Offline rTMS  Sham rTMS | left lateral parietal cortex selected based on high resting-state fMRI connectivity with the hippocampus  figure-8 coil | 5 sessions, 20-min stimulation, 1600 pulses at 20 Hz per session | Episodic memory (object recognition and recollection of object associations) | Stimulation improved recollection to a greater extent than recognition |
| *tDCS studies* | | | | | | | | |
| de Sousa, 2020 | 32 | 69±7 | No memory concerns or objective cognitive impairment | Within-subject Online atDCS  stDCS | Anode: right temporoparietal cortex  Cathode: left supraorbital area | 3 sessions with CT, 20- session stimulation, 1 mA | Episodic memory (Associative object-location memory) | No beneficial effect of atDCS for episodic memory |
| Freidle, 2020 | 123 | atDCS-1 (69.3±2.7)  atDCS-2 (69.8±2.9)  stDCS-1 (69.6±3.0)  stDCS-2 (69.8±2.6) | None | Between-subject atDCS-1: online atDCS (working memory training) (n=32)  atDCS-2: online atDCS (control training) (n=30)  stDCS-1: stDCS (working memory training) (n=33)  stDCS-2: stDCS (control training) (n=28) | Anode: lDLPFC (F3)  Cathode: left supraorbital area | 20 sessions with CT, 25-min stimulation, 2 mA | Mood  (POMS-2 + brief mood question) | No support for mood enhancement due to tDCS |
| Huo, 2018 | 64 | atDCS (66.5±6.2) stDCS (65.7±3.7) | MoCA≥21  Center for Epidemiologic Studies Depression Scale ≤16 | Between subjects Offline atDCS (n=31) stDCS (n=33) | Anode: lDLPFC (F3) Cathode: right deltoid muscle | 10 sessions, 30-min stimulation, 2 mA | Updating shifting and inhibition capacities (2-back, Flanker and switching task) | No differences between in performance due to atDCS or stDCS. |
| Jones, 2015 | 72 | 64.4±5.1 | MMSE>24 | Between subject  Online atDCS-1 (n=18)  Online atDCS-2 (n=18)  Online atDCS-3 (n=18)  stDCS (n=18) | Anode: F4 (atDCS-1); P4 (atDCS-2); F4 or P4 (atDCS-3)  Cathode: contralateral cheek | 10 sessions with CT,10-min stimulation, 1.5 mA | Working memory (ad hoc developed task) + transfer tasks (2-back, Stroop digit span) | Online atDCS groups maintained significant improvement in working memory at 1-year follow-up |
| Kulzow, 2018 | 32 | 68±7 | MMSE>25, CERAD memory within 1SD | Within-subject Online atDCS  stDCS | Anode: temporoparietal cortex (T6) Cathode: left supraorbital area | 3 sessions with CT,  20-min stimulation, 1 mA | Episodic memory (object-location pairings) | atDCS paired with CT did not enhance success in training or performance |
| Nilsson, 2017 | 123 | atDCS-1 (69.3±2.7)  atDCS-2 (69.8±2.9)  stDCS-1 (69.6±3.0)  stDCS-2 (69.8±2.6) | None | atDCS-1: online atDCS (working memory training) (n=32)  atDCS-2: online atDCS (control training) (n=30)  stDCS-1: stDCS (working memory training) (n=33)  stDCS-2: stDCS (control training) (n=28) | Anode: left dlPFC, (F3)  Cathode: contralateral supraorbital area | 20 sessions with CT,  25-min stimulation, 2 mA | Working-memory (3-back task) | Stimulation did not modulate gains from pre- to post-test on latent factors of either trained or untrained tasks in a statistically significant manner |
| Nissim, 2019 | 28 | 74±7.3 | No evidence of cognitive impairment, MoCA>27 | Between subject online atDCS (n=14) stDCS (n=14) | Anode: rDLPFC (F4)  Cathode: lDLPFC (F3) | 10 sessions with CT, 20-min stimulation, 2mA | Working-memory (2-back task) | Target accuracy on 2-Back improved for active vs. sham at post-intervention. atDCS vs. stDCS increased in connectivity between the lDLPFC and rIPL post-intervention |
| Park, 2014 | 40 | atDCS (70.1±3.4) stDCS (69.4±3.1) | None | Between-subject online atDCS (n=20) stDCS (n=20) | Anode: bilateral prefrontal cortex (F3 and F4)  Cathode: non dominant arm | 10 sessions with CT, 30-min stimulation, 2 mA | Working memory (2-back task) | Working memory accuracy significantly improved in atDCS vs. stDCS |
| Perceval, 2020 | 60 | 67.1±6.0 | Normal (age-corrected) ranges at baseline cognitive testing | Between-subject online atDCS (n=30) stDCS (n=30) | Anode: lIFG. Cathode: contralateral supraorbital region | 5 sessions with CT, 20-min stimulation, 1 mA | Episodic memory (associations memory task) | Facilitatory eﬀect of atDCS on immediate learning for the free recall task speciﬁc to older adults with lower baseline learning ability |
| Stephens, 2016 | 90 | atDCS-1 68.6 (SD unknown)  atDCS-2 68.6 (SD unknown)  stDCS 69.1 (SD unknown) | MMSE >22 | Between-subject online atDCS-1 (n=30)  online atDCS-2 (n=30)  stDCS (n=30) | Anode: rDLPFC (F4) Cathode: contralateral cheek | atDCS-1: 5 sessions with CT, 15-min stimulation, 1 mA  atDCS-2: 5 sessions with CT, 15-min stimulation, 2 mA | Working memory (2-back task) and transfer (ecological measures) | atDCS induced signiﬁcantly greater far transfer gains after 1 month |
| *Offline: stimulation is delivered before/after/without the cognitive training. Online: stimulation is delivered online during the cognitive training; CT: cognitive training; rTMS: repetitive transcranial magnetic stimulation; r/l DLPFC: right/left dorsolateral prefrontal cortex; MoCA-Bj: Montreal Cognitive Assessment-Beijing Version; MMSE: Mini Mental State Examination; CVLT: California Verbal Learning Test; atDCS: anodal transcranial direct current stimulation; stDCS sham tDCS; POMS-2: Profile of Mood States Second Edition; CERAD Consortium to Establish a Registry for Alzheimer's Disease; rIPL: right inferior parietal lobule; lIFG: left inferior frontal gyrus* | | | | | | | | |

1. Alonso-Coello, P., et al., *GRADE Evidence to Decision (EtD) frameworks: a systematic and transparent approach to making well informed healthcare choices. 2: Clinical practice guidelines.* BMJ, 2016. **353**: p. i2089.

2. Guyatt, G.H., et al., *GRADE: an emerging consensus on rating quality of evidence and strength of recommendations.* BMJ, 2008. **336**(7650): p. 924-6.
