# Supplementary material for: Protocols for cognitive enhancement. A user manual for Brain Health Services—part 5 of 6
Source: Alzheimers Res Ther. 2021 Oct 11;13:172. doi: 10.1186/s13195-021-00844-1 (PMC8507160; doi:10.1186/s13195-021-00844-1)
Supplement: Supplementary file 2 — Additional file 2. [file 13195_2021_844_MOESM2_ESM.pdf]

Brain Health Services: Organization, structure and challenges for implementation.

A user manual for Brain Health Services – Part 1 of 6

Daniele Altomare*, PhD[1,2]; José Luis Molinuevo*, MD[3]; Craig Ritchie, MD[4]; Federica Ribaldi, MS[1,2,5,6]; Emmanuel Carrera, MD[7]; Bruno Dubois, MD[8]; Frank Jessen, MD[9]; Laura McWhirter, MS[4]; Philip Scheltens, MD[10,11]; Wiesje M. van der Flier, PhD[10,12]; Bruno Vellas, MD[13]; Jean-François Démonet[+], MD[14]; Giovanni B. Frisoni[+], MD[1,2] *on behalf of the European Task Force for Brain Health Services*

*These authors contributed equally to this work (shared first author).

[+]These authors contributed equally to this work (shared last author).

[1]Laboratory of Neuroimaging of Aging (LANVIE), University of Geneva, Geneva, Switzerland.

[2]Memory Clinic, Geneva University Hospitals, Geneva, Switzerland.

[3]Barcelonaβeta Brain Research Center, Pasqual Maragall Foundation, Barcelona, Spain.

[4]Centre for Clinical Brain Sciences, University of Edinburgh, Edinburgh, UK.

[5]Laboratory of Alzheimer's Neuroimaging and Epidemiology (LANE), Saint John of God Clinical Research Centre, Brescia, Italy.

[6]Department of Molecular and Translational Medicine, University of Brescia, Brescia, Italy.

[7]Stroke Center, Department of Neurology, University Hospitals and University of Geneva, Geneva, Switzerland.

[8]Institut de la Mémoire et de la Maladie d'Alzheimer, IM2A, INSERM, Institut du Cerveau et de la Moelle Épinière, UMR-S975, Groupe Hospitalier Pitié-Salpêtrière, AP-HP, Sorbonne Université, Paris, France.

[9]Department of Psychiatry and Psychotherapy, Medical Faculty, University of Cologne, Cologne, Germany.

[10]Alzheimer Center Amsterdam, Department of Neurology, Amsterdam Neuroscience, Vrije Universiteit Amsterdam, Amsterdam UMC, Amsterdam, The Netherlands.

[11]Life Science Partners, Amsterdam, The Netherlands.

[12]Department of Epidemiology and Biostatistics, Vrije Universiteit Amsterdam, Amsterdam UMC, Amsterdam, The Netherlands.

[13]Gérontopole of Toulouse, University Hospital of Toulouse (CHU-Toulouse), Toulouse, France.

[14]Centre Leenaards de la Mémoire, Centre Hospitalier Universitaire Vaudois, Lausanne, Switzerland.

Corresponding author

Daniele Altomare

Memory Clinic, Geneva University Hospitals

Rue Gabrielle-Perret-Gentil 6, 1205 Geneva (Switzerland)

Email: Daniele.Altomare@unige.ch. Tel.: +41 22 372 58 00.

MANUSCRIPT DETAILS

Character count title (with spaces): 134.

Word count abstract: 268.

Word count manuscript: 3998.

Number of references: 41.

Number of figures: 1.

ABSTRACT

Dementia has a devastating impact on the quality of life of patients and families, and comes with a huge cost to society. Dementia prevention is considered a public health priority by the World Health Organization. Delaying the onset of dementia by treating associated risk factors will bring huge individual and societal benefit. Empirical evidence suggests that, in higher-income countries, dementia incidence is decreasing as a result of healthier lifestyles. This observation supports the notion that preventing dementia is possible and that a certain degree of prevention is already in action. Further reduction of dementia incidence through deliberate prevention plans is needed to counteract its growing prevalence due to increasing life expectancy.

An increasing number of individuals with normal cognitive performance seek help in the current memory clinics asking an evaluation of their dementia risk, preventive interventions, or interventions to ameliorate their cognitive performance. Consistent evidence suggests that some of these individuals are indeed at increased risk of dementia. This new health demand asks for a shift of target population, from patients with cognitive impairment to worried but cognitively unimpaired individuals. However, current memory clinics do not have the programs and protocols in place to deal with this new population.

We envision the development of new services, henceforth called Brain Health Services, devoted to respond to demands from cognitively unimpaired individuals concerned about their risk of dementia. The missions of Brain Health Services will be: i) dementia risk profiling, ii) dementia risk communication, iii) dementia risk reduction, and iv) cognitive enhancement. In this paper, we present the organizational and structural challenges associated with the set-up of Brain Health Services.

Keywords

Brain Health Services; dementia; aging; Alzheimer’s disease; prevention; dementia risk; risk communication; risk reduction; cognitive enhancement; personalized medicine.

1. BACKGROUND

Dementia consists of the cognitive decline from a previous level of performance to such an extent that it interferes with independence in everyday activities.[1] It impacts patients and their families and comes with a huge cost to society. Dementia prevention therefore is considered a public health priority by the World Health Organization.[2] Delaying the onset of dementia by treating underlying diseases will bring huge individual and societal benefit. Empirical observations in cohorts born in more recent decades in high-income countries indicate a reduction of the age-specific incidence of dementia,[3–14] suggesting that dementia prevention is possible and already in action. This is likely the unintended result of greater overall wealth and healthier lifestyles, including better control of cardiovascular risk factors. Epidemiological evidence allows to estimate that 40% of dementia cases are due to lifestyle and cardiovascular modifiable risk factors,[15] while the remaining cases are largely explained by genetic (e.g. *APOE* ε4), biological (e.g. amyloid and tau), other unknown risk factors, and their interactions.[16] However, the bad news is that dementia prevalence is steadily increasing worldwide. This is due mainly to population aging in lower- and middle- income countries, and to the increased life span of individuals with dementia in higher income countries. Therefore, a further decrease of dementia incidence is needed to counteract the worldwide trend of increased dementia prevalence. We believe that, today, evidence is sufficient to set up evidence-based, effective, personalized, and equitable dementia prevention plans in persons at risk of dementia.

The current memory clinics have been designed for the needs of patients with overt cognitive and/or behavioral disorders with the aim of reducing the burden of progressive decline (*tertiary prevention*). Nevertheless, a considerable number of cognitively unimpaired individuals believing that they may be at increased risk of dementia is seeking help in memory clinics, accounting for 20-30% of all patients.[17–19] Increasing and consistent evidence indicates that these have a mildly increased risk of dementia as compared to the general population.[20] These individuals present specific concerns, requests, expectations and hopes different from those of the cognitively impaired ones, but they are usually discharged with generic recommendations and reassurance, and no really actionable and meaningful answers.

The development of new and innovative services, henceforth referred to as Brain Health Services (BHSs), is needed to provide specific answers to these individuals' unmet needs. The missions of BHSs consist of: i) dementia risk profiling, ii) dementia risk communication, iii)

dementia risk reduction (*primary* and *secondary prevention*), and iv) cognitive enhancement. BHSs will feature specific knowledge, skills, protocols and technology to meet the challenges posed by this new demand. Some pilot experiences are ongoing at the time of the writing of this article (Q4 2020) in Barcelona, Edinburgh, and Paris; and have provided ideas and tools for this article and the others of this series published in this issue of *Alzheimer's Research & Therapy*.

This is the first of six papers, which are part of a larger initiative of the European Task Force for Brain Health Services, aiming to draft the protocols of operations in the BHSs of the future. Here we describe the organization, structure and challenges for implementing BHSs, while the other papers focus on the four missions (mentioned above) and on the societal challenges.

2. BHS ORGANIZATION

In this section, we present how the novel BHS facilities might be structured at the time of writing of this article (Q4 2020). In Section 4.1, we envision how BHSs may look like in the upcoming years based on research advances and technological innovations.

2.1. What is in a name?

Equally tenable denominations could be proposed, emphasizing different aspects: i) the biomedical domains (e.g. brain, dementia, Alzheimer's disease, memory), ii) the clinical mission (e.g. health, prevention), and iii) the organizational structure (e.g. clinic, service, unit). The end result would be labels such as "brain health clinics", "dementia prevention services", etc.

We propose "Brain Health Services" for the following reasons: i) the concept of "brain health" is more comprehensive than "dementia prevention", opening to cognitive enhancement which, by definition, aims to improve cognitive functions rather than preventing dementia, and is one of people's demands; and ii) the terms "clinic" and "unit" imply that the services would be delivered in structures independent of other services, while we believe that BHSs can be implemented either within the current memory clinics or as distributed and interconnected services (see Section 2.7). Whatever the label, its semantic should match the content of the health offer.

2.2. Users

The target population of BHSs will consist of older adults who wish to check their risk of dementia, preserve cognitive functions, or enhance their cognitive performance. This population includes individuals with subjective cognitive decline (SCD),[21] functional cognitive disorders,[19] and the "worried wells".

Individuals with SCD experience persistent cognitive decline which is not detected by the standard clinical and neuropsychological batteries used to detect mild cognitive impairment and dementia and, although cognitively unimpaired, have an increased risk of dementia as

compared to the general population (incidence of 20.1/1000 person-year vs 14.2/1000 person-year[20]). Functional cognitive disorders consist of a range of overlapping conditions in which cognitive symptoms, usually of attentional nature, present characteristic internal inconsistency as the result of reversible changes in brain function rather than damage or disease.[19] Functional cognitive disorders may present as an isolated syndrome, or in the context of anxiety or depression, or alongside other functional or somatoform symptoms such as chronic pain.[19] Some individuals with functional cognitive disorders may perform in the mildly impaired range on cognitive tests.[19] Where a positive diagnosis of functional cognitive disorders is made, appropriate treatment should include a clear explanation of the diagnosis using supportive written material (for example, "Functional Neurological Disorder (FND): a patient's guide"[22]). Worried wells do not have any specific cognitive complaint, but they claim concern of declining cognition in the future, and strive to preserve it as long as possible or even enhance it. Worried wells frequently report a family history of dementia or Alzheimer's disease.

Clearly, the target population of BHSs is remarkably different from that of current memory clinics. The above case-mix suggests not to refer to them as "patients", but to rather prefer a more neutral term such as "users".

It is worth noting that the definition and identification of the ideal target population might not always be clear-cut, especially when it comes to individuals with borderline or inconclusive cognitive testing or very mild cognitive or executive dysfunctions who might still benefit of the BHS offers.

In the perspective of growing demand and growing offer, in the early years of BHSs, users will be referred by memory clinics. At a later stage, as BHSs catch up, they will consolidate their own user flow consisting of people who spontaneously show up directly to BHSs. The implementation of educational programs (e.g. awareness campaigns on brain health for the general population) and other initiatives[23] might increase the BHSs visibility and reputation.

2.3. Missions

The four main missions of BHS are: i) dementia risk profiling, ii) dementia risk communication, iii) dementia risk reduction (*primary and secondary prevention*), and iv) cognitive enhancement. These topics are exhaustively discussed in the pertinent papers

(Ranson et al., *this issue*; Visser et al., *this issue*; Solomon et al., *this issue*; Brioschi et al., *this issue*), and briefly summarized below.

Education of the general public and health care providers might be a mission of BHSs in academic settings. This will not be addressed in this paper as it will be of interest to a minority of academic BHSs and is beyond the scope of this initiative.

2.3.1. Dementia risk profiling

The very first step of assessment in BHSs will be understanding the user's request. Anecdotal observations indicate that a number of individuals with SCD, functional cognitive disorders, or worried wells look for reassurance. Indeed, malaises such as psychological/psychiatric (e.g. depression, trauma, affective issues) or personal issues (e.g. divorce, violent spouse, unemployment, societal issues) are sometimes presented in disguise as "memory" concerns. A careful history collection, carried out with tact and empathy, can be revealing. In such cases, a "blind" offer of dementia risk assessment would be a clinical misstep. The BHS clinician should here refer the user to the appropriate specialist.

The following step to the implementation of personalized prevention plans is to identify users' risk factors for dementia. The relative risk of modifiable dementia risk factors varies widely between 1.1 for air pollution to 1.9 for hearing loss and depression[15], and dramatically increases for genetic (*APOE* ε4 genotype) and biological (amyloid and tau deposition) risk factors.[16] BHSs must be able to comprehensively assess, combine (e.g. through composite dementia risk scores such as the Cardiovascular Risk Factors, Aging, and Incidence of Dementia (CAIDE) Dementia Risk Score[24]; the Brief Dementia Screening Indicator[25]; and the Australian National University Alzheimer's Disease Risk Index[26,27]), and interpret all these risk factors together with protective factors, and to finally profile and categorize the user's specific risk into strata (e.g. high, moderate, or low risk of developing dementia in the following 5, 10, or 15 years).

Further research is still needed to: estimate the relative risk of each risk factor adjusted for communality with other factors; develop composite risk scores combining modifiable, biological (e.g. amyloid and tau), and genetic (e.g. *APOE*) risk factors; and develop cost-effective screening protocols (Ranson et al., *this issue*).

2.3.2. Dementia risk communication

The communication of the concept of risk to the general public is challenging, especially in the context of untreatable and stigmatized neurodegenerative diseases leading to dementia. Evidence on how to communicate dementia risk is scarce. Nevertheless, the available literature allows to put in place some practical recommendation (see Visser et al., *this issue* for further information). These recommendations are inspired from other fields with more experience on this topic (e.g. oncology[28–30]), and from existing research disclosure protocols of genetic (e.g. *APOE* genotype[31–34]) and biomarker (e.g. amyloid-PET[35–39]) results that proved to have a well-tolerated psychological impact in the short term. Nevertheless, we acknowledge that these disclosure protocols are limited to explaining that genes or biomarkers are risk factors for dementia, but do not actually communicate the risk of developing dementia in the next future.

We underline that the use of standardized communication protocols on an individual level is challenging and might require a certain degree of adaptation and clinical sensitivity. Further research is needed to develop communication protocols delivering quantitative information about individual risk, and scalable tools suitable to users with different socio-demographic and cultural features (including educational background). BHSs will represent the ideal context for this research.

2.3.3. Dementia risk reduction

Risk reduction interventions aim to reduce the likelihood of long-term cognitive decline or dementia onset in at-risk individuals. Among all the randomized trials on multi-domain interventions, only the FINGER study met its primary outcome, showing greater cognitive improvement in participants of the experimental group versus controls.[40] On the contrary, other randomized trials on multi-domain interventions such as MAPT,[41] preDIVA,[42] Look AHEAD,[43] and DO-HEALTH[44] failed to meet their primary outcomes.

Interestingly, subsample analyses of the FINGER and MAPT studies showed that interventions were more effective in patients at increased risk for dementia based on genetic (*APOE* ε4)[45] or biological (amyloid positivity)[41] risk factors. This suggests that personalized multi-domain

interventions, tailored to the individual's specific risk factors (reflecting the risk reduction potential), are likely associated to the highest clinical benefit.

Even though preliminary evidence is now available and allows to provide recommendations for practical implementation of precision dementia risk reduction interventions (Solomon et al., *this issue*), long-term multi-domain randomized controlled trials are needed to provide definitive evidence on their efficacy. The World Wide-FINGERS, the first network for multimodal dementia prevention trials, aims to fill this evidence gap by adapting and optimizing the FINGER operational model for dementia risk reduction in different populations, and geographic and economic settings.[46]

Translation of experimental risk reduction interventions to the clinical setting will not be straightforward. Possible interventions that can be offered to BHS users today or in the next future might cover one of more of the following areas: diet, exercise, cognitive training, and vascular risk monitoring (inspired by current cardiovascular prevention programs) (Solomon et al., *this issue*).

2.3.4. Cognitive enhancement

Cognitive enhancement interventions aim to improve the individual's performance and abilities. These interventions are typically performed over a time span of a few days/weeks. Cognitive enhancement interventions include cognitive, mental and physical training (including mindfulness); non-invasive brain stimulation; and cognitive-enhancement drugs. To date, currently available evidence supports the use of cognitive training, while evidence supporting the efficacy of mindfulness and tDCS interventions might possibly increase in the next few years. Evidence on cognitive-enhancing drugs is poor and inconclusive (Brioschi et al., *this issue*).

2.4. Personnel and expertise

The dementia domain is largely interdisciplinary and spans neurology, geriatrics, psychiatry, cognitive psychology, neuropsychology, nursing, and social sciences. Expertise in psychology and/or neuropsychology is necessary for the initial (and potential follow-up) clinical and

cognitive evaluations, to communicate the risk, and to implement cognitive interventions. Medical expertise (e.g. in neurology, geriatrics, psychiatry) is necessary to define indications for entering the BHS track, carry out exams, interpret biological and genetic risk factors, prioritize risk, set risk reduction interventions, and propose follow-up if needed. Nursing competences might be necessary to collect samples and measures for risk factor assessment (e.g. biological samples, blood pressure). Further expertise, such as nutrition or a physical training, might be useful to cover some specific areas of prevention.

BHSs will wish to recruit personnel based on the required expertise rather than on a priori defined professional categories. For example, although current job descriptions usually suggest that dementia risk communication should be done by a physician, we believe that a psychologist with appropriate training, empathy and communication skills can safely perform this task. Post-graduate courses on the care of persons with cognitive disorders that are active or being launched in Europe will help educate BHS professionals.[47]

2.5. Basic vs advanced BHSs

Not all BHSs will need to cover the whole range of potential health offer. We envision at least two levels, basic and advanced, depending on resources and available facilities. Basic services may consist of i) standardized risk assessment based on lifestyles, vascular and basic genetic risk factors (e.g. *APOE*), and possibly measures reflecting structural brain health (e.g. qualitative or quantitative measures of atrophy and vascular changes), implementing low-level composite dementia risk scores (e.g. the CAIDE Dementia Risk Score); ii) adaptation and use of current practices for dementia risk communication; iii) implementation of standardized non-pharmacological prevention protocols (e.g. FINGER and MAPT interventions) and pharmacological and non-pharmacological control of cardiovascular risk factors; and iv) cognitive enhancement using cognitive training.

An advanced version of BHSs may expand the basic services with one or more of the following: i) molecular imaging biomarkers (e.g. amyloid-PET, tau-PET, MRI with automated image post-processing) and/or CSF biomarkers (e.g. $A\beta_{42}$, phosphorylated tau, neurofilament light), ii) use of structured personalized dementia risk communication protocols taking into account specific user's features (e.g. educational background), iii) implementation of personalized prevention protocols tailored to the user's molecular risk profile including biomarker derived

information, iv) combination of sophisticated and personalized cognitive enhancement techniques (e.g. cognitive training and non-invasive brain stimulation).

2.6. Facilities

The main technological facilities needed in a BHSs are largely the same of a traditional memory clinic and might include MRI, PET, and fully-automated CSF analysis platforms (e.g. Elecsys, Lumipulse).

Other facilities will be specific to BHSs and may include tablets for computerized cognitive training, physical activity monitors, and fitness trackers.

As is the case of current memory clinics, local factors such as availability of technology or expertise, or idiosyncrasies towards a given diagnostic or intervention technology will give individual BHSs their specificity.

2.7. Context for BHS implementation

BHSs can be either hybrid or stand-alone services. In the first case, BHSs can leverage on the current memory clinics' structure and ongoing collaborations (with nuclear medicine, radiology, biochemistry laboratories, etc.). BHS-specific expertise and technology will need to be integrated, since some personnel and facilities are often lacking in memory clinics such as psychotherapists, nutrition experts, physical trainers, and devices for transcranial stimulation. The investment in this case would be relatively modest. In the second case, stand-alone BHSs will need new personnel and facilities and to build collaborations with other services. The investment in this case would obviously be significantly higher. In either case, since stroke centers are already dealing with the implementation of cardiovascular prevention programs and the promotion of awareness-rising campaigns (both key aspects of BHSs), BHSs can partner with them and leverage on their longstanding expertise.

Whether the setup, a tight collaboration between BHSs and memory clinics is strongly encouraged by this working group. Indeed, memory clinics can refer cognitively unimpaired patients to BHSs in order to investigate their request and provide meaningful answers.

Conversely, BHSs can refer cognitively impaired users to memory clinics in order to start proper diagnostic workup and treatment.

3. BHS CHALLENGES

3.1. Equity and societal challenges

One of the main challenges will consist in making BHSs equitable, i.e. accessible to the general population regardless of their economic status. Most interventions potentially offered by BHSs are not reimbursed in any Western country, they may take place in for-profit enterprises where users pay interventions with out-of-pocket money. Indeed, BHSs, at least at their first development stages, will thrive mainly in higher-income countries for the greater social awareness of cognitive diseases. As a consequence, access may be limited to the more affluent and more highly educated members of society. See Milne et al. (*this issue*) for further information on this topic. The affiliation to an existing memory clinic or stroke center might facilitate the coverage of healthcare insurances.

3.2. Individual interventions vs large-scale population interventions

The European Task Force for Brain Health Services is largely made of clinicians and clinical researchers who are by mission focused on individuals rather than on society as a whole. Indeed, even though BHSs can sporadically touch the general population (e.g. by awareness promoting campaigns on brain health), their mission is the implementation of personalized prevention plans tailored to the individual's risk profile. This is the so-called "high-risk approach" that has contributed to dramatically decrease stroke morbidity and mortality over the past decades.[48]

Nevertheless, the authors acknowledge that well-designed and implemented prevention initiatives at the population level might be associated with great societal benefit, if only in the long term. Such interventions require the direct engagement of healthcare systems and payers, and strong evidence supporting the efficacy of interventions.[49] BHSs may contribute to the production of this evidence, while they may or may not be the hubs of prevention initiatives at the population level.

3.3. Sustainability

Depending on the context (see Section 2.7), a BHSs will require variable amount of funding to be financially sustainable. It is likely that business models for BHSs will develop through several stages. Initial resources may come from grants, philanthropy and channeling research income/overheads into the establishment of innovator sites that will by necessity be located in university teaching hospitals. Such settings will not need to invest heavily in up-front capital costs for e.g. MRI scanners. These settings must commit to generating substantial evidence on access and health outcomes to deliver both short- and long- term health economic analysis. These will be locally derived to take to the local health care funders and will be nuanced to reflect the needs/motivations of the purchaser.

The purchasing by the extant health providers has to be the exit strategy for the reactive initial funding. One could argue that a 5-year period of funding for 'pilot or innovator' sites is sufficient to make the argument to transition to centralized funding by e.g. Healthcare Commissioners in the NHS. This will be supported by e.g. NICE guidance and other clinical policy documents that will support individual practitioners in making their business case. Reports from advocacy groups whilst helpful are no replacement for policy documents generated in an unbiased fashion by organizations like NICE. Finally, the patient perspectives on the service can act as a powerful motivator for change. Collecting data on their experience will help the development of services as well as their extension to other regions of the country in question.

Of course, investors in the market of private healthcare may also wish to seize the opportunity of investing in this growing market. The setups of BHSs in already existing structures (e.g. memory clinics or stroke centers) will minimize the amount of the investment.

3.4. Research

In order to promote equity and sustainability, BHSs should integrate their offer with continuing research activity. Sound evidence produced by BHSs research activity might contribute to: i) identifying the trajectories of the underlying pathologies by the follow-up of individuals at a preclinical stage, ii) selecting individuals at high risk for the inclusion in clinical trials aimed at studying the efficacy of disease modifying drugs at a preclinical asymptomatic stage of the

disease, iii) producing strong scientific evidence on the efficacy of interventions (or lack thereof), iv) making structural efforts to access more marginalized communities by design, v) drawing attention of healthcare systems and persuade them to provide coverage, making BHS sustainable and equitable.

4. DISCUSSION

The increasing prevalence of dementia, the awareness of the general population on brain health, recent advancements in technology and knowledge of neurodegenerative diseases, and preliminary evidence of effective risk reduction interventions constitute the rationale behind the development of BHSs. BHSs will focus on a new target population (cognitively unimpaired individuals concerned with the preservation or improvement of their cognitive abilities); have specific missions (dementia risk profiling, dementia risk communication, dementia risk reduction, and cognitive enhancement); face relevant challenges (demonstrating efficacy, equity and sustainability of the services); and require high-level expertise, facilities and personnel. BHSs might rely on the current memory clinics or be independent services.

4.1. The future of BHSs

We envision that BHSs might change in the upcoming years thanks to research advances and novel technologies.

4.1.1. Dementia risk profiling

The clinical validation of blood-based biomarkers of amyloid,[50] tau,[51] and neurodegeneration (e.g. neurofilament light[52]) will radically change the way individual risk is assessed. Indeed, blood-based biomarkers are much cheaper than molecular imaging and much more accessible. We envision a scenario where blood-based biomarkers with high sensitivity for abnormality will be used for large-scale dementia screening, thus reducing the number of users requiring more expensive testing. Polygenic risk scores may also make the transition to clinical fruition in the coming years. The widespread use of calculators (e.g. ADappt[53]) will allow a comprehensive interpretation of multiple risk factors and the quantification of the user's risk. Finally, the use of brain health registries[54,55] and digital tools will facilitate the access of users to BHSs.

4.1.2. Dementia risk communication

Large-scale education programs will result in increased awareness of the general population on brain health. A more educated and aware population has a better predisposition to understand the concept of risk. Nevertheless, further research is needed to develop and implement proper communication strategies on an individual level.

4.1.3. Dementia risk reduction

Aducanumab[56,57] might be the very first disease modifying therapy approved by the FDA for clinical use in patients with prodromal Alzheimer's disease or mild Alzheimer's disease dementia. Several phase 3 clinical trials on anti-amyloid drugs in cognitively healthy individuals are currently ongoing, and their results are expected between 2021 and 2025.[58] If they prove to be effective, disease modifying therapies will be the main weapon to prevent cognitive deterioration in cognitively unimpaired biomarker-positive individuals. However, whether disease modifying therapies will be available or not, more targeted personalized multidomain interventions will be increasingly fine-tuned and implemented in BHSs.[46]

4.1.4. Cognitive enhancement

In the next future, protocols combining cognitive training, mindfulness and non-invasive brain stimulation might be available, although the timelines are even harder to predict than for industry-sponsored pharmacological clinical trials.

The Figure provides an example of how BHSs might operate.

4.2. Conclusion

Despite the many organizational and structural challenges to be faced, we envision that the development of BHSs will play a key role in the fight against the increasing dementia prevalence by embracing the needs of cognitively unimpaired individuals who wish to preserve or improve their cognitive abilities.

LIST OF ABBREVIATIONS

BHS: Brain Health Services. CAIDE: Cardiovascular Risk Factors, Aging, and Incidence of Dementia. MRI: magnetic resonance imaging. SCD: subjective cognitive decline.

DECLARATIONS

Ethics approval and consent to participate: Not applicable.

Consent for publication: Not applicable.

Availability of data and materials: Data sharing is not applicable to this article as no datasets were generated or analyzed during the current study.

Competing interests

JLM is currently a full-time employee of Lundbeck and has previously served as a consultant or at advisory boards for the following for-profit companies, or has given lectures in symposia sponsored by the following for-profit companies: Roche Diagnostics, Genentech, Novartis, Lundbeck, Oryzon, Biogen, Lilly, Janssen, Green Valley, MSD, Eisai, Alector, BioCross, GE Healthcare, ProMIS Neurosciences.

BD has received research funding (paid to the institution) from Merck-Avenir Foundation, Roche and consultancy fees from Biogen, Neurodiem, Green Valley, Cytox, Brainstorm. He is PI of clinical trials with EISAI, Genentech, Novartis, Biogen, Roche.

PS has received consultancy fees (paid to the institution) from AC Immune, Alkermes, Alnylam, Anavex, Biogen, Brainstorm Cell, Cortexyme, Denali, EIP, ImmunoBrain Checkpoint, GemVax, Genentech, Green Valley, Novartis, Novo Noridisk, PeopleBio, Renew LLC, Roche. He is PI of studies with AC Immune, CogRx, FUJI-film/Toyama, IONIS, UCB, Vivoryon. He serves on the board of the Brain Research Center.

WMvdF has received consultancy fees (paid to the institution) from Oxford Health Policy Forum CIC, Roche BV. She has been an invited speaker at Boehringer Ingelheim, Biogen MA Inc, and WebMD Neurology (Medscape). She has performed contract research for Biogen MA

Inc and Boehringer Ingelheim. All funding is paid to her institution. WF is associate editor at *Alzheimer's Research & Therapy*.

JFD has received consultancy fees from Biogen and OM Pharma; unrestricted grants from OM Pharma; and has collaboration agreements with Siemens and MindMaze.

GBF reports grants from Alzheimer Forum Suisse, Académie Suisse des Sciences Médicales, Avid Radiopharmaceuticals, Biogen, GE International, Guerbert, Association Suisse pour la Recherche sur l'Alzheimer, IXICO, Merz Pharma, Nestlé, Novartis, Piramal, Roche, Siemens, Teva Pharmaceutical Industries, Vifor Pharma, and Alzheimer's Association; he has received personal fees from AstraZeneca, Avid Radiopharmaceuticals, Elan Pharmaceuticals, GE International, Lundbeck, Pfizer, and TauRx Therapeutics.

The other coauthors declare that they have no competing interests.

Funding

This paper was the product of a workshop funded by the Swiss National Science Foundation entitled "Dementia Prevention Services" (grant number: IZSEZ0_193593).

GBF received funding by: the EU-EFPIA Innovative Medicines Initiatives 2 Joint Undertaking (IMI 2 JU) "European Prevention of Alzheimer's Dementia consortium" (EPAD, grant agreement number: 115736) and "Amyloid Imaging to Prevent Alzheimer's Disease" (AMYPAD, grant agreement number: 115952); the Swiss National Science Foundation: "Brain connectivity and metacognition in persons with subjective cognitive decline (COSCODE): correlation with clinical features and in vivo neuropathology" (grant number: 320030_182772).

WMvdF holds the Pasman chair.

Authors' contribution

Daniele Altomare and José Luis Molinuevo conceptualized this Paper, drafted the manuscript for intellectual content, and approved the manuscript.

Jean-François Démonet conceptualized this Paper, revised the manuscript for intellectual content, and approved the manuscript.

Craig Ritchie, Bruno Dubois, and Laura McWhirter drafted specific parts of the manuscript, revised the manuscript for intellectual content, and approved the manuscript.

Emmanuel Carrera, Frank Jessen, Philip Scheltens, Wiesje M. van der Flier, and Bruno Vellas revised the manuscript for intellectual content, and approved the manuscript.

Daniele Altomare, Giovanni B. Frisoni, and Federica Ribaldi conceived and organized the workshop whence the Papers of the BHS series in this issue of *Alzheimer's Research & Therapy* originated, conceived the related editorial initiative, revised this manuscript for intellectual content, harmonized the manuscript with the other Papers of the BHS series, and approved the manuscript.

Acknowledgments

European Task Force for Brain Health Services (in alphabetical order): Marc ABRAMOWICZ, Daniele ALTOMARE, Frederik BARKHOF, Marcelo BERTHIER, Melanie BIELER, Kaj BLENNOW, Carol BRAYNE, Andrea BRIOSCHI, Emmanuel CARRERA, Gael CHÉTELAT, Chantal CSAJKA, Jean-François DEMONET, Alessandra DODICH, Bruno DUBOIS, Giovanni B. FRISONI, Valentina GARIBOTTO, Jean GEORGES, Samia HURST, Frank JESSEN, Miia KIVIPELTO, David LLEWELLYN, Laura McWHIRTER, Richard MILNE, Carolina MINGUILLÓN, Carlo MINIUSSI, José Luis MOLINUEVO, Peter M NILSSON, Janice RANSON, Federica RIBALDI, Craig RITCHIE, Philip SCHELTENS, Alina SOLOMON, Wiesje VAN DER FLIER, Cornelia VAN DUIJN, Bruno VELLAS, Leonie VISSER.

REFERENCES

1. APA. *Diagnostic and statistical manual of mental disorders (DSM-5®).* (American Psychiatric Pub, 2013).

2. WHO | Dementia: a public health priority. https://www.who.int/mental_health/publications/dementia_report_2012/en/.

3. Schrijvers, E. M. C. *et al.* Is dementia incidence declining? Trends in dementia incidence since 1990 in the Rotterdam Study. *Neurology* **78**, 1456–1463 (2012).

4. Langa, K. M. Is the risk of Alzheimer's disease and dementia declining? in *Alzheimer's Research and Therapy* vol. 7 (BioMed Central Ltd., 2015).

5. Knopman, D. S. The Enigma of Decreasing Dementia Incidence. *JAMA Netw. open* **3**, e2011199 (2020).

6. Wolters, F. J. *et al.* Twenty-seven-year time trends in dementia incidence in Europe and the United States: The Alzheimer Cohorts Consortium. *Neurology* **95**, e519–e531 (2020).

7. Wu, Y. T. *et al.* Dementia in western Europe: Epidemiological evidence and implications for policy making. *The Lancet Neurology* vol. 15 116–124 (2016).

8. Satizabal, C. L. *et al.* Incidence of Dementia over Three Decades in the Framingham Heart Study. *N. Engl. J. Med.* **374**, 523–532 (2016).

9. Matthews, F. E. *et al.* A two decade dementia incidence comparison from the Cognitive Function and Ageing Studies I and II. *Nat. Commun.* **7**, (2016).

10. Prince, M. *et al.* Recent global trends in the prevalence and incidence of dementia, and survival with dementia. *Alzheimer's Res. Ther.* **8**, (2016).

11. Wu, Y. T. *et al.* The changing prevalence and incidence of dementia over time-current evidence. *Nature Reviews Neurology* vol. 13 327–339 (2017).

12. Derby, C. A., Katz, M. J., Lipton, R. B. & Hall, C. B. Trends in dementia incidence in a birth cohort analysis of the Einstein Aging Study. *JAMA Neurol.* **74**, 1345–1351 (2017).

13. Roehr, S., Pabst, A., Luck, T. & Riedel-Heller, S. G. Is dementia incidence declining in high-income countries? A systematic review and meta-analysis. *Clinical Epidemiology* vol. 10 1233–1247 (2018).

14. Tom, S. E. *et al.* Association of Demographic and Early-Life Socioeconomic Factors by Birth Cohort With Dementia Incidence Among US Adults Born Between 1893 and 1949. *JAMA Netw. open* **3**, e2011094 (2020).

15. Livingston, G. *et al.* Dementia prevention, intervention, and care: 2020 report of the Lancet Commission. *The Lancet* vol. 396 413–446 (2020).

16. Frisoni, G. B. *et al.* Precision prevention of Alzheimer's and other dementias: Anticipating future needs in the control of risk factors and implementation of disease-modifying therapies. *Alzheimer's Dement.* (2020) doi:10.1002/alz.12132.

17. Van Der Flier, W. M. *et al.* Optimizing patient care and research: The Amsterdam dementia cohort. *J. Alzheimer's Dis.* **41**, 313–327 (2014).

18. Hejl, A., Høgh, P. & Waldemar, G. Potentially reversible conditions in 1000 consecutive memory clinic patients. *J. Neurol. Neurosurg. Psychiatry* **73**, 390–394 (2002).

19. McWhirter, L., Ritchie, C., Stone, J. & Carson, A. Functional cognitive disorders: a systematic review. *The Lancet Psychiatry* vol. 7 191–207 (2020).

20. Slot, R. E. R. *et al.* Subjective cognitive decline and rates of incident Alzheimer's disease and non–Alzheimer's disease dementia. *Alzheimer's Dement.* **15**, 465–476 (2019).

21. Jessen, F. *et al.* The characterisation of subjective cognitive decline. *The Lancet Neurology* vol. 19 271–278 (2020).

22. Memory Concentration - neurosymptoms.org. https://www.neurosymptoms.org/memory-concentration/4594358003.

23. Giovannoni, G. *et al.* Brain Diseases - Time matters: a call to prioritize brain health. https://www.oxfordhealthpolicyforum.org/reports/brain-diseases/brain-diseases-report (2019).

24. Kivipelto, M. *et al.* Risk score for the prediction of dementia risk in 20 years among middle aged people: a longitudinal, population-based study. *Lancet Neurol.* **5**, 735–741 (2006).

25. Barnes, D. E. *et al.* Development and validation of a brief dementia screening indicator for primary care. *Alzheimer's Dement.* **10**, 656-665.e1 (2014).

26. Anstey, K. J., Cherbuin, N. & Herath, P. M. Development of a New Method for Assessing Global Risk of Alzheimer's Disease for Use in Population Health Approaches to Prevention. *Prev. Sci.* **14**, 411–421 (2013).

27. Anstey, K. J. *et al.* A self-report risk index to predict occurrence of dementia in three independent cohorts of older adults: The ANU-ADRI. *PLoS One* **9**, (2014).

28. Zipkin, D. A. *et al.* Evidence-based risk communication: A systematic review. *Annals of Internal Medicine* vol. 161 270–280 (2014).

29. Fagerlin, A., Zikmund-Fisher, B. J. & Ubel, P. A. Helping patients decide: Ten steps to better risk communication. *Journal of the National Cancer Institute* vol. 103 1436–1443 (2011).

30. van de Water, L. F. *et al.* Communicating treatment risks and benefits to cancer patients: a systematic review of communication methods. *Quality of Life Research* vol. 29 1747–1766 (2020).

31. Green, R. C. *et al.* Disclosure of APOE Genotype for Risk of Alzheimer's Disease . *N. Engl. J. Med.* **361**, 245–254 (2009).

32. Chao, S. *et al.* Health behavior changes after genetic risk assessment for Alzheimer disease: The REVEAL study. *Alzheimer Dis. Assoc. Disord.* **22**, 94–97 (2008).

33. Bemelmans, S. A. S. A. *et al.* Psychological, behavioral and social effects of disclosing Alzheimer's disease biomarkers to research participants: a systematic review. *Alzheimer's Res. Ther.* **8**, 1–17 (2016).

34. Langlois, C. M. *et al.* Alzheimer's Prevention Initiative Generation Program: Development of an APOE genetic counseling and disclosure process in the context of clinical trials. *Alzheimer's Dement. Transl. Res. Clin. Interv.* **5**, 705–716 (2019).

35. Harkins, K. *et al.* Development of a process to disclose amyloid imaging results to cognitively normal older adult research participants. *Alzheimer's Res. Ther.* **7**, (2015).

36. Burns, J. M. *et al.* Safety of disclosing amyloid status in cognitively normal older adults. *Alzheimer's Dement.* **13**, 1024–1030 (2017).

37. Largent, E. A. *et al.* Cognitively unimpaired adults' reactions to disclosure of amyloid PET scan results. *PLoS One* **15**, (2020).

38. Grill, J. D. *et al.* Short-term Psychological Outcomes of Disclosing Amyloid Imaging Results to Research Participants Who Do Not Have Cognitive Impairment. *JAMA Neurol.* **77**, (2020).

39. De Wilde, A. *et al.* Disclosure of amyloid positron emission tomography results to individuals without dementia: A systematic review. *Alzheimer's Res. Ther.* **10**, (2018).

40. Ngandu, T. *et al.* A 2 year multidomain intervention of diet, exercise, cognitive training, and vascular risk monitoring versus control to prevent cognitive decline in at-risk elderly people (FINGER): A randomised controlled trial. *Lancet* **385**, 2255–2263 (2015).

41. Andrieu, S. *et al.* Effect of long-term omega 3 polyunsaturated fatty acid supplementation with or without multidomain intervention on cognitive function in elderly adults with memory complaints (MAPT): a randomised, placebo-controlled trial. *Lancet Neurol.* **16**, 377–389 (2017).

42. van Charante, E. P. M. *et al.* Effectiveness of a 6-year multidomain vascular care intervention to prevent dementia (preDIVA): a cluster-randomised controlled trial. *Lancet* **388**, 797–805 (2016).

43. Espeland, M. A. *et al.* Long-term Impact of Weight Loss Intervention on Changes in Cognitive Function: Exploratory Analyses From the Action for Health in Diabetes Randomized Controlled Clinical Trial. *J Gerontol A Biol Sci Med Sci* **73**, 484–491 (2018).

44. Bischoff-Ferrari, H. A. *et al.* Effect of Vitamin D Supplementation, Omega-3 Fatty Acid Supplementation, or a Strength-Training Exercise Program on Clinical Outcomes in Older Adults: The DO-HEALTH Randomized Clinical Trial. *JAMA - J. Am. Med. Assoc.*

324, 1855–1868 (2020).

45. Solomon, A. *et al.* Effect of the Apolipoprotein E genotype on cognitive change during a multidomain lifestyle intervention a subgroup analysis of a randomized clinical trial. *JAMA Neurol.* **75**, 462–470 (2018).

46. Kivipelto, M. *et al.* World-Wide FINGERS Network: A global approach to risk reduction and prevention of dementia. *Alzheimer's Dement.* **16**, 1078–1094 (2020).

47. CAS HES-SO en Démences et troubles psychiques de la personne âgée 2020-2021 | HEdS. https://www.hesge.ch/heds/formation-continue/formations-postgrades/certificats-cas/cas-hes-so-en-demences-et-troubles.

48. Elkind, M. S. V. Implications of stroke prevention trials: Treatment of global risk. *Neurology* vol. 65 17–21 (2005).

49. Brainin, M. *et al.* Global prevention of stroke and dementia: the WSO Declaration. *Lancet Neurol.* **19**, 487–488 (2020).

50. Verberk, I. M. W. *et al.* Plasma Amyloid as Prescreener for the Earliest Alzheimer Pathological Changes. *Ann. Neurol.* **84**, 648–658 (2018).

51. Mattsson, N. *et al.* Plasma tau in Alzheimer disease. *Neurology* **87**, 1827–1835 (2016).

52. Mattsson, N., Cullen, N. C., Andreasson, U., Zetterberg, H. & Blennow, K. Association between Longitudinal Plasma Neurofilament Light and Neurodegeneration in Patients with Alzheimer Disease. *JAMA Neurol.* **76**, 791–799 (2019).

53. Van Maurik, I. S. *et al.* Development and usability of ADappt:Web-based tool to support clinicians, patients, and caregivers in the diagnosis of mild cognitive impairment and Alzheimer disease. *J. Med. Internet Res.* **21**, (2019).

54. Hersenonderzoek.nl. https://hersenonderzoek.nl/.

55. Brain Health Registry Switzerland. http://www.bhr-suisse.org/en.

56. Haeberlein, S. B. *et al.* EMERGE and ENGAGE Topline Results: Two Phase 3 Studies to Evaluate Aducanumab in Patients With Early Alzheimer's Disease. https://investors.biogen.com/static-files/ddd45672-9c7e-4c99-8a06-3b557697c06f

(2019).

57. Schneider, L. A resurrection of aducanumab for Alzheimer’s disease. *Lancet Neurol.* **19**, 111–112 (2020).

58. NIA-Funded Active Alzheimer’s and Related Dementias Clinical Trials and Studies. https://www.nia.nih.gov/research/ongoing-AD-trials#section2.

Figure. BHS activities today and in the next future, in comparison with memory clinics.

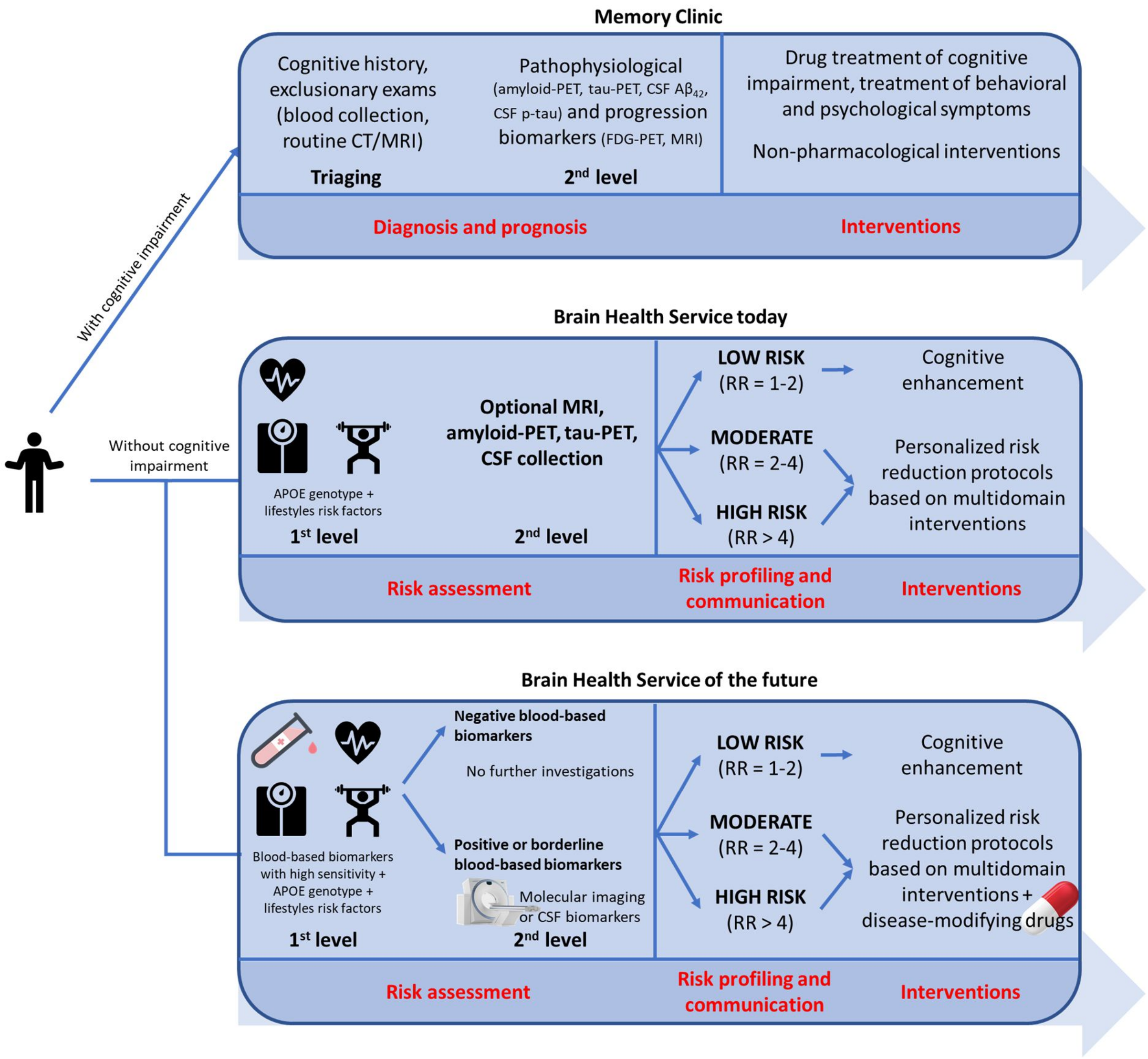

This Figure provides an example of how BHSs might operate when blood-based biomarkers are available. In this scenario, users undergo screening including assessment of *APOE* genotype and lifestyle risk factors as well as high-sensitivity blood-based biomarkers. Those users with positive or borderline blood-based biomarkers might also undergo a second-level assessment with molecular imaging (e.g. amyloid-PET, tau-PET), even if this might be not necessary if blood-based biomarkers prove to be highly accurate (in terms of both high sensitivity and specificity). Taken together, this information allows to profile the user's risk and classify it (e.g. as "low", "moderate", or "high" based on a composite relative risk). Afterward, the risk is communicated to the user. Finally, the intervention is chosen accordingly: users with low risk might start personalized cognitive enhancement interventions, while users with moderate or high risk should undergo personalized risk reduction interventions possibly

including disease-modifying therapies. This is a purely indicative scenario and can vary based on the context of BHS implementation.

CT: computerized tomography. MRI: magnetic resonance imaging. FDG: Fluorodeoxyglucose. PET: positron emission tomography. APOE: Apolipoprotein E. RR: relative risk.

The risk operationalization of “low” (RR = 1-2), “moderate” (RR = 2-4) and “high” (RR > 4) intended to be indicative and is used for illustrative purposes only.

Modifiable risk factors for dementia and dementia risk profiling.

A user manual for Brain Health Services – Part 2 of 6

Janice M. Ranson, PhD[1,2]; Timothy Rittman, PhD[3]; Shabina Hayat, PhD[4]; Carol Brayne, MD, FFPH[4]; Frank Jessen, MD[5]; Kaj Blennow, MD[6]; Cornelia van Duijn, PhD[7]; Frederik Barkhof, MD[8,9]; Eugene Tang, MBChB[10]; Catherine J. Mummery, PhD[11]; Blossom CM Stephan, PhD[12]; Daniele Altomare, PhD[13,14]; Giovanni B. Frisoni, MD[13,14]; Federica Ribaldi, MS[13,14,15,16]; José Luis Molinuevo, MD[17]; Philip Scheltens, MD[18,19]; David J. Llewellyn, PhD[1,2,20] *on behalf of the European Task Force for Brain Health Services*

[1]College of Medicine and Health, University of Exeter, UK.

[2]Deep Dementia Phenotyping Network, Exeter, UK.

[3]Department of Clinical Neurosciences, University of Cambridge.

[4]Department of Public Health and Primary Care, Cambridge Public Health, University of Cambridge, Cambridge, UK.

[5]Department of Psychiatry and Psychotherapy, Medical Faculty, University of Cologne, Cologne, Germany

[6]Department of Psychiatry and Neurochemistry, Institute of Neuroscience & Physiology, the Sahlgrenska Academy at the University of Gothenburg, Mölndal, Sweden; Clinical Neurochemistry Laboratory, Sahlgrenska University Hospital, Mölndal, Sweden.

[7]University of Oxford, Nuffield Department of Population Health, Oxford, UK.

[8]Centre for Medical Image Computing, Department of Medical Physics and Biomedical Engineering, University College London, London, UK.

[9]Department of Radiology and Nuclear Medicine, Amsterdam University Medical Centers, Amsterdam, The Netherlands.

[10]Newcastle University, Population Health Sciences Institute, Level 2, Newcastle Biomedical, Research Building, Campus for Ageing and Vitality, Newcastle upon Tyne, NE4 5PL.

[11]Dementia Research Centre, Institute of Neurology, University College London, and National Hospital for Neurology and Neurosurgery, University College London Hospital, London, UK.

[12]Institute of Mental Health, Division of Psychiatry and Applied Psychology, School of Medicine, Jubilee Campus, Nottingham University, Nottingham, UK, NG7 2TU.

[13]Laboratory of Neuroimaging of Aging (LANVIE), University of Geneva, Geneva, Switzerland.

[14]Memory Clinic, Geneva University Hospitals, Geneva, Switzerland.

[15]Laboratory of Alzheimer's Neuroimaging and Epidemiology (LANE), Saint John of God Clinical Research Centre, Brescia, Italy.

[16]Department of Molecular and Translational Medicine, University of Brescia, Brescia, Italy.

[17]Barcelonaβeta Brain Research Center (BBRC), Pasqual Maragall Foundation, Barcelona, Spain

[18]Alzheimer Center Amsterdam, Department of Neurology, Amsterdam Neuroscience, Vrije Universiteit Amsterdam, Amsterdam UMC, Amsterdam, the Netherlands

[19]Life Science Partners, Amsterdam, The Netherlands

[20]Alan Turing Institute, UK.

Corresponding author

Prof David J. Llewellyn

2.04 College House, St Luke's Campus, University of Exeter Medical School, Exeter, EX1 2LU, UK.

Email: david.llewellyn@exeter.ac.uk

MANUSCRIPT DETAILS

Character count title (with spaces): 110

Word count abstract: 274

Word count manuscript: 3507

Number of references: 93

Number of figures: 2

Number of tables: 2

ABSTRACT

We envisage the development of new Brain Health Services to achieve primary and secondary dementia prevention. These services will complement existing memory clinics by targeting cognitively unimpaired individuals, where the focus is on risk profiling and personalized risk reduction interventions rather than diagnosing and treating late-stage disease. In this review we outline key potentially modifiable risk factors, fluid, genetic and imaging risk factors. We then provide practical guidelines for the assessment of risk factors and risk profiling in Brain Health Services. Users of Brain Health Services should undergo risk profiling tailored to their age, level of risk, and availability of local resources. Initial risk assessment should incorporate a multidomain risk profiling measure. For users aged 39-64 we recommend the Cardiovascular Risk Factors, Aging, and Incidence of Dementia (CAIDE) Dementia Risk Score; whereas for users aged 65 and older we recommend the Brief Dementia Screening Indicator (BDSI) and the Australian National University Alzheimer's Disease Risk Index (ANU-ADRI). The initial assessment should also include potentially modifiable risk factors including sociodemographic, lifestyle and health factors. If resources allow, *apolipoprotein E ε4* status testing and structural magnetic resonance imaging should be conducted. If this initial assessment indicates a low dementia risk, then low intensity interventions can be implemented. If the user has a high dementia risk, additional investigations should be considered if local resources allow. Common variant polygenic risk of late-onset AD can be tested in middle-aged or older adults. Rare variants should only be investigated in users with a family history of early-onset dementia in a first degree relative. Advanced imaging with 18-Fluorodeoxyglucose positron emission tomography (FDG-PET) or amyloid PET may be informative in high risk users to clarify the nature and burden of their underlying pathologies. Cerebrospinal fluid biomarkers are not recommended for this setting, and blood-based biomarkers need further validation before clinical use. As new technologies become available, advances in artificial intelligence are likely to improve our ability to combine diverse data to further enhance risk profiling. Ultimately, Brain Health Services have the potential to reduce the future burden of dementia through risk profiling, risk communication, personalized risk reduction and cognitive enhancement interventions.

Keywords

Alzheimer’s disease; dementia; aging; brain health services; risk factors; risk profiling; prevention; public health

1. BACKGROUND

In absence of disease modifying treatments for Alzheimer's disease (AD) and other dementias, Frisoni and colleagues[1] highlighted the rationale for primary and secondary dementia prevention, and the need for new services aimed at cognitively unimpaired individuals. Primary prevention strategies for individuals with unknown disease markers include modifiable risk factors, lifestyle and multiple domain interventions to reduce disease incidence. Secondary prevention targets high risk cognitively unimpaired individuals with biomarker evidence of disease pathology, to prevent or delay symptom onset.

Current memory clinics are ill-equipped to deal with the number of cognitively unimpaired individuals seeking help in memory clinics who believe they may be at increased risk of dementia (Altomare et al., *this issue*). For this reason, we envision the development of new Brain Health Services (BHSs) with specific missions including dementia risk profiling, risk communication (Visser et al., *this issue*), risk reduction (Solomon et al., *this issue*), and cognitive enhancement (Brioschi et al., *this issue*). Admittedly, BHSs pose specific societal challenges (Milne et al., *this issue*).

This review focuses on the first principle of risk profiling, and is the second part of a Special Issue series of six articles, published in *Alzheimer's Research & Therapy*, which together provide a user manual for BHSs. We provide an overview of modifiable risk factors and their interaction with genetic risk factors, before discussing best practices for the assessment of risk factors in a BHS setting. We then outline multiple domain measures and risk profiling in the context of primary and secondary prevention services. Finally, we provide practical guidelines for BHSs, and consider possible uncertainties, inconsistencies and challenges.

2. RISK FACTORS

2.1. Overview of modifiable risk factors

The concept of prevention being better than cure underpins the growing interest in the role of modifiable risk factors for cognitive impairment and dementia[2]. The 2020 Report of the *Lancet* Commission identified 12 modifiable risk factors, which, with appropriate interventions, could prevent up to 40% of dementia cases worldwide[3]. This may particularly benefit low- and middle-income countries where the prevalence of dementia is thought to be rising faster than in higher income countries[3].

Education is an early life potentially modifiable risk factor linked to late-life dementia risk[3], either by exerting a direct effect on brain structure by, for example, improving vascularization contributing to cognitive reserve, or by shaping healthier behaviors that reduce cardiovascular and cerebrovascular damage[4]. If causality is assumed and low levels of education were eliminated, then it has been estimated this would lead to a 7% reduction in dementia prevalence[3].

Hearing loss, traumatic brain injury (TBI), hypertension, alcohol consumption (>21 units per week), and obesity have been identified as key potentially modifiable midlife dementia risk factors[3]. Poor encoding of sound may affect brain structure and function, impose higher cognitive load and reduce social interaction[5]. Oxidative stress, inflammatory effects and reduced cerebral flow contribute to brain pathology associated with factors such as TBI and hypertension[6]. The percentage reduction in dementia prevalence if these risk factors were eliminated ranges from 8% for hearing loss to 1% for alcohol and obesity[3].

Diabetes, smoking, air pollution, depression, social isolation and physical inactivity have been identified as potentially modifiable late life dementia risk factors[3]. Diabetes[7] and physical inactivity[8] are associated with an adverse vascular profile, which itself is associated with an increased dementia risk[8]. Diabetes increases inflammation and oxidative stress on the brain[9]. Smoking and air pollution enhance reactive oxygen and inflammatory responses [6, 10]. Depression and social isolation are associated with accelerated brain and cardiovascular

ageing[11] and poor health behaviours[12]. The reduction in dementia prevalence associated with elimination of these risk factors ranges from 5% for smoking to 1% for diabetes[3].

As our understanding of modifiable risk factors improves, new risk factors are likely to be added to this list of 'key' risk factors. For example, emerging evidence suggests that diet, cognitive stimulation, vitamin D and pesticide exposure may also be important[13, 14]. Based upon current evidence, the 12 potentially modifiable dementia risk factors identified in the 2020 Report of the *Lancet* Commission should be incorporated into BHS assessments and prioritized in personalized interventions.

2.2. Genetic risk factors and interactions with modifiable risk factors

AD is the most common type of dementia and has a strong genetic component, involving both common and rare genetic variants[15] (see Figure 1). To date, 34 genetic variants have been associated with AD[16]. Specifically, *PSEN1*, *PSEN2* and *APP* mutations cause AD dementia in virtually 100% of carriers (autosomal dominant AD[17]), with a mean age at dementia onset of 35-65 years[18] and symptom duration of about 10 years[19]. Nevertheless, the major driver of AD in the general population is the common apolipoprotein E ε4 variant (*APOE* ε4), which is associated with an elevated risk of developing dementia, i.e. 51-95% in *APOE* ε4/ε4 and 22-90% in *APOE* ε4/-[20, 21], and a mean age at dementia onset of 73-74 years in *APOE* ε4/ε4 and 75-82 in *APOE* ε4/-[22, 23]. Although common variants often have very small effects on a person's AD risk, jointly they may modify the risk and age at onset of Alzheimer's disease and dementia significantly[16].

Our knowledge of the genes implicated in non-AD dementias is less comprehensive. Mutations in microtubule associated protein tau gene and the gene encoding progranulin are specific for frontotemporal dementia. Other variants including the intronic expansion of a hexanucleotide repeat in C9orf72 and SERPINA1 are also implicated, though few common variants have been identified (see Figure 1). For dementia with Lewy bodies, only *APOE*, Glucocerebrosidase and Synuclein Alpha have been replicated. Lastly, for vascular dementia there are no consistent findings for common variants.

It is unknown whether genetic risk modifies the influence of lifestyle on dementia. Four large multidomain trials of dementia prevention have been conducted. However, only the Finnish Geriatric Intervention Study to Prevent Cognitive Impairment and Disability found a significant difference in the primary outcome following a lifestyle, metabolic and vascular intervention[24]. The beneficial effect was only observed in *APOE ε4* carriers[25]. Null group level findings in other trials may therefore mask effects in genetic subgroups. Comparable findings from observational studies are mixed. There was no interaction between lifestyle factors and polygenic risk score in relation to all-cause dementia risk in the UK Biobank[26]. However, there was a significant interaction between a composite of lifestyle and health factors and APOE/polygenic risk in relation to all-cause dementia in the Rotterdam Study[27]. Taken together, these findings provide suggestive rather than conclusive evidence that, contrary to expectation, those with a high genetic risk may be more likely to benefit from targeted dementia prevention interventions.

2.3. Assessing risk factors in the clinic

When assessing cognitively unimpaired users in BHSs it is important to consider their profile of modifiable lifestyle and clinical risk factors in order to inform personalized and targeted dementia prevention interventions. Fluid and neuroimaging biomarkers can be used to differentiate between asymptomatic individuals with and without underlying pathology. This can be used to target people who are particularly likely to benefit from interventions. Assessment of pathology also provides a baseline for disease burden that can be subsequently used to assess rates of progression. Nevertheless, the use of biomarkers in BHSs depends on local facilities and resources and on the context of BHS implementation (Altomare et al., *this issue*).

2.3.1. Assessing potentially modifiable risk factors

The potentially modifiable risk factors to be assessed in BHSs are shown in Table 1, along with examples of methods to assess these factors in a clinical setting.

2.3.2. Assessing fluid (CSF and plasma) risk factors

Many studies have consistently shown that core AD cerebrospinal fluid (CSF) biomarkers amyloid β ($A\beta_{42}$ and $A\beta_{42}/A\beta_{40}$ ratio), total-tau (T-tau), and phosphorylated tau (P-tau) reflect key elements of AD pathophysiology, have high diagnostic value and high concordance with amyloid Positron Emission Tomography (PET)[39]. However, there are currently no disease specific fluid biomarkers for non-AD dementia. Furthermore, a lumbar puncture is often regarded as complicated and invasive and subjects may have side-effects in the form of transient headache. Thus, for a BHS clinical setting, blood biomarkers are likely to be more practical and acceptable to users than CSF biomarkers.

Technical developments have allowed for quantification of brain-specific proteins in blood samples. For amyloidosis, the plasma $A\beta_{42}/A\beta_{40}$ ratio shows high concordance with amyloid PET[40], and can be measured on fully automatized instruments[41]. Blood biomarkers for tau pathology include P-tau181, which shows a marked increase in AD and high concordance with tau PET[42, 43], while levels are normal in other tauopathies, such as frontotemporal dementia. Importantly, plasma P-tau181 is increased in unimpaired elderly having brain amyloidosis, but still a negative tau PET scan[44]. This suggests it may be sensitive to pathological change at an earlier stage. Studies of other tau variants, specifically P-tau217, show encouraging results[45]. Neurofilament light (NFL) is a well-validated neurodegeneration biomarker showing increases in several neurodegenerative disorders, including AD[46], and predicts future rate of cognitive decline[47]. Importantly, plasma NFL increases early in the preclinical phase of AD[48, 49]. While blood biomarkers are very promising they need further real-world validation before they can be recommended for use in BHSs[50, 51].

2.3.3. Assessing genetic risk factors

Combining effects of APOE*4 and common variants allows a precise prediction of the risk and age of onset of AD[16, 52, 53], and pathology in the brain[54]. Age specific risk curves may have clinical utility in BHS, allowing determination of future risk of AD at different stages of the life course[16]. These estimates can be provided using polygenic risk scores based on replicated variants[16] assessed by dedicated AD chips or putative genome wide variants that are marginally associated to the disease that can be assessed by general genetics arrays[52-54]. Although in many

countries testing for rare variant conferring a high risk of AD (see Figure 1) is the domain of clinical genetics, routine testing for such variants in a BHS may be useful for users with a family history of AD. Within a BHS, testing for known major genes that harbor rare variants may be done in collaboration with clinical geneticists. As is the case with many complex disorders, rare variants in genes will be encountered for which the functional effects and the risk of AD is yet unknown in archives such as Omim and ClinVar[55]. However, collaboration between the genomics and clinical community could facilitate genetic counselling in the setting of a BHS[55].

2.3.4. Assessing imaging risk factors

Magnetic resonance imaging (MRI) promises to be a sensitive early biomarker of neurodegeneration given that genetic cohorts of dementia demonstrate structural MRI changes many years before symptom onset[56-58]. The finding of selective early hippocampal atrophy is well established in AD[58, 59], and is an accepted biomarker for clinical trials[60, 61], yet translating this into detecting early AD for clinical use requires further work[62]. However, there are a number of potential methodological developments in artificial intelligence, PET and MRI technology that may lead to more specific and biologically relevant neuroimaging biomarkers[63].

Cerebrovascular risk is a particular focus for neuroimaging studies and impacts on cognition in healthy aging[64]. While silent territorial infarcts are relatively rare, cerebral small vessel disease is extremely common, encompassing white matter hyperintensities, lacunes, widened Virchow-Robin spaces and cerebral microbleeds[65]. White matter hyperintensities are a frequent finding associated with cardiovascular risk factors such as hypertension and smoking. They significantly increase the risk of stroke, dementia and overall mortality[66], especially when lesions become confluent[67]. Stroke itself is a strong independent risk factor for incident all-cause dementia[68]. Lacunes are found more frequently in individuals with atrial fibrillation and present an independent risk factor for cognitive decline. Cerebral microbleeds can be due to cardiovascular risk factors deep in the basal nuclei, while lobar cerebral microbleeds are reflective of amyloid-angiopathy; they only carry a weak risk for dementia on a population level[69].

Current consensus practice for assessing MRI scans is to use visual rating scales, such as the medial temporal lobe atrophy scale[70], the parietal atrophy scale[71], the global cortical atrophy scale[72], the age-related white matter changes[73], and the Fazekas scale for white matter lesions[74, 75]. Measurement of regional cortical thickness can also identify presymptomatic amyloid positive individuals[76]. However, with the advent of artificial intelligence, new neuroimaging tools for diagnosis and prognosis are emerging[77-79] that may provide more sensitive assessments in the near future.

PET has provided a suite of tools for assessing people with cognitive impairment using specific ligands that bind to physiological targets. The most well established in clinical practice is 18-Fluorodeoxyglucose (FDG) PET which has proved useful for predicting cognitive impairment in Parkinson's disease[80]. Ligands for beta-amyloid have found the presence of beta-amyloid increases with age, reaching 65% in health over-80s[81]. However, a positive beta-amyloid PET did not correlate to cognition, so the implications of this finding remains uncertain for predicting risk. It has been shown in genetic forms of AD that amyloid accumulates 15-20 years prior to symptom onset and it is thought to be an early critical factor in disease, although changes in amyloid load do not reliably correlate with cognitive change[56]. Other ligands for tau[82] inflammation[83] or synaptic integrity[84] exist, but remain in the research domain. The cost and availability of PET imaging may limit it's applicability to BHSs, but could have a role in selected high risk individuals.

3. RISK PROFILING

3.1. Multidomain measures and risk profiling

A number of dementia risk prediction models have been developed to determine dementia risk in middle-aged or older adults[85, 86]. The validity of most risk models is unknown, as is the degree to which they can be appropriately used in different populations. Prediction models which have been validated in multiple samples include the Cardiovascular Risk Factors, Aging and Dementia (CAIDE) score[87], the Australian National University Alzheimer's Disease Risk Index (ANU-ADRI)[88, 89] and the Brief Dementia Screening Indicator (BDSI)[90]. Basic characteristics of these models are shown in Table 1. The CAIDE score assesses long-term risk of dementia in middle-aged adults, whereas the ANU-ADRI and the BDSI predict medium-term AD and dementia risk respectively in older adults. The overall accuracy of these risk prediction models is moderate (range 0.64-0.78), indicating that, although they can be improved upon, they can also generate useful predictions. It is notable that 10 of the 12 modifiable risk factors for dementia included in the 2020 Report of the *Lancet* Commission are included in these models[3]. The only modifiable risk factors identified in that report which are not included are hearing loss and air pollution.

The CAIDE score has a moderate level of discriminative accuracy over 20 years follow-up (Area Under the Curve (AUC) = 0.77, 95% CI = 0.71-0.83). The addition of APOE e4 did not substantially increase accuracy (AUC = 0.78, 95% CI = 0.72-0.84). When this model was externally validated it performed similarly in a midlife population (AUC = 0.75)[91] but poorly in late-life populations with shorter follow-up times (AUC range: 0.49–0.57)[89]. When tested in three cohorts the ANU-ADRI was found to have moderate levels of discriminative accuracy for Alzheimer's disease: Rush Memory and Aging Project study AUC = 0.64 (95% CI = 0.60 – 0.68); Kungsholmen Project study AUC = 0.74 (95% CI 0.71–0.77); Cardiovascular Health Cognition study AUC = 0.73 (95% CI = 0.69–0.78). The BDSI was tested in four cohorts including the Cardiovascular Health Study (CHS), The Framingham Heart Study (FHS), the Health and Retirement Study (HRS) and the Sacramento Area Latino Study on Aging (SALSA). The discrimination accuracy of the final model was moderate across cohorts: CHS AUC = 0.68 (95% CI = 0.65-0.72); FHS AUC = 0.77 (95% CI = 0.73-0.82); HRS AUC = 0.76 (95% CI = 0.74-0.77); SALSA AUC = 0.78 (95% CI = 0.72-0.83).

There have also been attempts to develop new models in at-risk subpopulations. For example, the Diabetes-Specific Dementia Risk Score (DSDRS) is a model for type 2 diabetics. The DSDRS was found to have reasonable accuracy in the development (AUC=0.74) and validation (AUC=0.75) cohorts[91]. Disease specific predictive models may be important as generic dementia risk prediction models may not work well in specific subpopulations[92]. Further, not all prediction models for dementia developed in high-income countries are necessarily applicable to low- and middle-income countries[93].

4. DISCUSSSION

4.1. Summary

We now have a reasonable idea what the 'key' potentially modifiable dementia risk factors are in early-, mid- and late- life. It is also likely that further risk factors will be added to this list in the future. Rare variants for early-onset dementia have been identified, and common variants for late-onset dementia, particularly AD, are now known. Further research is needed to investigate possible gene-environment interactions CSF biomarkers are not very practical in the context of BHSs, however blood-based biomarkers may be useful subject to further real-world validation. Structural MRI is becoming established as a clinically useful imaging biomarker of dementia pathologies, and advanced imaging may be a useful supplement to this if available. Existing dementia risk prediction models offer a practical way of risk profiling individual users, though there is room for improvement and they have not yet been optimized for use in BHSs.

4.2. Practical guidelines

The assessment of risk factors and risk profiling in BHSs will require a multidisciplinary team, and a balance between precision and practicality. The initial assessment should include the exclusion of pre-existing dementia. Individual assessment of modifiable risk factors is likely to involve multiple measures and may prove to be time consuming. Some assessments may be completed in advance of the appointment, and a specialist nurse appointment may also enrich the information available. The individual will undergo an assessment tailored to their age, level of risk following an initial assessment, and local resources available (see Figure 2). A follow-up communication of the user's results will be required (Visser et al., *this issue*), followed by the proposal of an individualized plan for risk reduction (Solomon et al., *this issue*) and/or cognitive enhancement interventions (Brioschi et al., *this issue*) and/or clinical trial opportunities.

Initial BHS risk profiling should incorporate an age appropriate multidomain risk profiling measure, assessment of additional risk factors, *APOE* ε4 status if possible, and structural MRI.

To assess long-term dementia risk in middle-aged individuals aged 39-64 we recommend that BHSs use the CAIDE score. The CAIDE should not be used for anyone younger than 39 whose dementia risk will be negligible over 20 years, or in those aged 65 years or older as accuracy is poor in older adults and better alternatives are available. *APOE* ε4 genotyping should be undertaken if possible to permit use of the full CAIDE model, and as multimodal interventions may be more effective in e4 carriers[25]. This will allow for targeted allocation of limited resources when attempting dementia prevention. To assess medium-term dementia risk in individuals aged 65 and older we recommend that BHSs use the BDSI. This can be complemented by the additional administration of the ANU-ADRI which produces a comparable risk prediction for AD specifically. The ANU-ADRI also has the practical advantage of incorporating a larger number of modifiable risk factors such as smoking and physical activity which can inform targeted interventions. Additional risk factor assessment should focus on those factors with the strongest evidence base and greatest opportunity to intervene, particularly those outlined in the 2020 Report of the *Lancet* Commission [3]. These can be divided into early life (education), midlife (hearing loss, TBI, hypertension, alcohol consumption, and obesity), and late-life (diabetes, smoking, air pollution, depression, social isolation and physical inactivity), and should be assessed routinely taking into account the user's age. Some, but not all, of these risk factors are incorporated into existing dementia risk scores (see Table 1). Structural MRI should be used in BHSs to enable the assessment of non-degenerative pathologies (eg inflammation, tumors), cerebrovascular burden (particularly cerebral small vessel disease including white matter hyperintensities and lacunes), and neurodegenerative processes (generalized, medial temporal lobe and hippocampal atrophy).

If initial BHS dementia risk profiling indicates that the user has a low risk of dementia then low intensity interventions can be implemented. If however the initial risk profiling indicates that the user has a high risk of dementia then additional optional investigations should be considered if local facilities and resources allow. Common variant polygenic risk of late-onset AD can be tested in middle-aged or older adults. However rare variants should only be investigated in users with a family history of early-onset dementia (<65 years) in a first degree relative in collaboration with clinical geneticists. Advanced imaging with FDG-PET or amyloid PET may also prove to be informative in high risk patients to clarify their burden of underlying pathology. Plasma biomarkers may prove to be a useful additional optional investigation in high risk users in the near future, though are not currently recommended for use in BHSs before further real-world validation.

4.3. CONCLUSIONS

Risk profiling in BHS involves a core assessment comprised of multidomain risk prediction models in combination with additional risk factors, *APOE* ε4 status if possible, and structural MRI. If resources allow, then additional investigations including more comprehensive genetic testing and advanced neuroimaging can be undertaken in high risk users. Results can then be communicated to users, a personalized risk reduction and cognitive enhancement plan formulated, and clinical trial opportunities identified.

LIST OF ABBREVIATIONS

AD: Alzheimer's disease. ANU-ADRI: Australian National University Alzheimer's Disease Risk Index. *APOE:* apolipoprotein E. AUC: Area Under the Curve. BDSI: Brief Dementia Screening Indicator. BHS: Brain health services. CAIDE: Cardiovascular Risk Factors, Aging and Dementia. CHS: Cardiovascular Health Study. CSF: Cerebrospinal fluid. DSDRS: Diabetes-Specific Dementia Risk Score. FDG: 18-Fluorodeoxyglucose. FHS: Framingham Heart Study. HRS: Health and Retirement Study. MRI: Magnetic resonance imaging. Nfl: Neurofilament light. PET: Positron Emission Tomography. P-tau: Phosphorylated tau. SALSA: Sacramento Area Latino Study on Aging. TBI: Traumatic brain injury.

DECLARATIONS

Ethics approval and consent to participate: Not applicable

Consent for publication: Not applicable

Availability of data and materials: Data sharing is not applicable to this article as no datasets were generated or analyzed during the current study.

Competing interests

GBF reports grants from Alzheimer Forum Suisse, Académie Suisse des Sciences Médicales, Avid Radiopharmaceuticals, Biogen, GE International, Guerbert, Association Suisse pour la Recherche sur l'Alzheimer, IXICO, Merz Pharma, Nestlé, Novartis, Piramal, Roche, Siemens, Teva Pharmaceutical Industries, Vifor Pharma, and Alzheimer's Association; he has received personal fees from AstraZeneca, Avid Radiopharmaceuticals, Elan Pharmaceuticals, GE International, Lundbeck, Pfizer, and TauRx Therapeutics.

KB has served as a consultant, at advisory boards, or at data monitoring committees for Abcam, Axon, Biogen, JOMDD/Shimadzu. Julius Clinical, Lilly, MagQu, Novartis, Roche Diagnostics, and Siemens Healthineers, and is a co-founder of Brain Biomarker Solutions in Gothenburg AB (BBS), which is a part of the GU Ventures Incubator Program.

JLM is currently a full time employee of Lundbeck and has previously served as a consultant or at advisory boards for the following for-profit companies, or has given lectures in symposia sponsored by the following for-profit companies: Roche Diagnostics, Genentech, Novartis, Lundbeck, Oryzon, Biogen, Lilly, Janssen, Green Valley, MSD, Eisai, Alector, BioCross, GE Healthcare, ProMIS Neurosciences.

All other authors declare that they have no competing interests.

Funding

This paper was the product of a workshop funded by the Swiss National Science Foundation entitled "Dementia Prevention Services" (grant number: IZSEZ0_193593).

JMR is supported by Alzheimer's Research UK.

DJL is supported by the National Institute for Health Research (NIHR) Applied Research Collaboration (ARC) South West Peninsula, Alzheimer's Research UK, National Health and Medical Research Council (NHMRC), JP Moulton Foundation, National Institute on Aging/National Institutes of Health (RF1AG055654), Alan Turing Institute/Engineering and Physical Sciences Research Council (EP/N510129/1).

CJM is supported by the National Institute for Health Research University College London Hospitals Biomedical Research Centre.

GBF received funding from the EU-EFPIA Innovative Medicines Initiatives 2 Joint Undertaking (IMI 2 JU) "European Prevention of Alzheimer's Dementia consortium" (EPAD, grant agreement number: 115736) and "Amyloid Imaging to Prevent Alzheimer's Disease" (AMYPAD, grant agreement number: 115952); the Swiss National Science Foundation: "Brain connectivity and metacognition in persons with subjective cognitive decline (COSCODE): correlation with clinical features and in vivo neuropathology" (grant number: 320030_182772).

KB is supported by the Swedish Research Council (#2017-00915), the Swedish Alzheimer Foundation (#AF-742881), Hjärnfonden, Sweden (#FO2017-0243), and the Swedish state under the agreement between the Swedish government and the County Councils, the ALF-agreement (#ALFGBG-715986).

CD is supported by the CoSTREAM project (European Union's Horizon 2020 research and innovation programme under grant agreement number 667375)

ET is funded by the National Institute for Health Research (NIHR) School for Primary Care Research. The views expressed are those of the author(s) and not necessarily those of the NIHR or the Department of Health and Social Care.

Authors' contributions

Janice M. Ranson and David J Llewellyn contributed to drafting and revision of the manuscript for intellectual content.

Timothy Rittman, Shabina Hayat, Carol Brayne, Frank Jessen, Kaj Blennow, Cornelia van Duijn, Frederik Barkhof, Eugene Tang, Blossom Stephan, Catherine Mummery contributed to drafting and revision of the manuscript for intellectual content.

José Luis Molinuevo and Philip Scheltens contributed to the conception of the work.

Daniele Altomare, Giovanni B. Frisoni, and Federica Ribaldi conceived and organized the workshop whence the Papers of the BHS series in this issue of *Alzheimer's Research & Therapy* originated, conceived the related editorial initiative, revised this manuscript for intellectual content, harmonized the manuscript with the other Papers of the BHS series, and approved the manuscript.

Acknowledgments

European Task Force for Brain Health Services (in alphabetical order): Marc ABRAMOWICZ, Daniele ALTOMARE, Frederik BARKHOF, Marcelo BERTHIER, Melanie BIELER, Kaj BLENNOW, Carol BRAYNE, Andrea BRIOSCHI, Emmanuel CARRERA, Gael CHÉTELAT, Chantal CSAJKA, Jean-François DEMONET, Alessandra DODICH, Bruno DUBOIS, Giovanni B. FRISONI, Valentina GARIBOTTO, Jean GEORGES, Samia HURST, Frank JESSEN, Miia KIVIPELTO, David LLEWELLYN, Laura McWHIRTER, Richard MILNE, Carolina MINGUILLÓN, Carlo MINIUSSI, José Luis MOLINUEVO, Peter M NILSSON, Janice RANSON, Federica RIBALDI, Craig RITCHIE, Philip SCHELTENS, Alina

SOLOMON, Wiesje VAN DER FLIER, Cornelia VAN DUIJN, Bruno VELLAS, Leonie VISSER.

REFERENCES

1. Frisoni GB, Molinuevo JL, Altomare D, et al. Precision prevention of Alzheimer's and other dementias: Anticipating future needs in the control of risk factors and implementation of disease-modifying therapies. Alzheimers Dement 2020.

2. Livingston G, Sommerlad A, Orgeta V, et al. Dementia prevention, intervention, and care. Lancet 2017;390:2673-2734.

3. Livingston G, Huntley J, Sommerlad A, et al. Dementia prevention, intervention, and care: 2020 report of the Lancet Commission. Lancet 2020;396:413-446.

4. Arenaza-Urquijo EM, Wirth M, Chételat G. Cognitive reserve and lifestyle: moving towards preclinical Alzheimer's disease. Front Aging Neurosci 2015;7:134.

5. Peelle JE, Troiani V, Grossman M, Wingfield A. Hearing loss in older adults affects neural systems supporting speech comprehension. J Neurosci 2011;31:12638-12643.

6. Hughes TF, Ganguli M. Modifiable Midlife Risk Factors for Late-Life Cognitive Impairment and Dementia. Curr Psychiatry Rev 2009;5:73-92.

7. Leon BM, Maddox TM. Diabetes and cardiovascular disease: Epidemiology, biological mechanisms, treatment recommendations and future research. World J Diabetes 2015;6:1246-1258.

8. Kivimäki M, Singh-Manoux A, Pentti J, et al. Physical inactivity, cardiometabolic disease, and risk of dementia: an individual-participant meta-analysis. BMJ 2019;365:l1495.

9. Shalev D, Arbuckle MR. Metabolism and Memory: Obesity, Diabetes, and Dementia. Biological psychiatry 2017;82:e81-e83.

10. Peters R, Ee N, Peters J, Booth A, Mudway I, Anstey KJ. Air Pollution and Dementia: A Systematic Review. J Alzheimers Dis 2019;70:S145-s163.

11. Cacioppo JT, Hawkley LC, Norman GJ, Berntson GG. Social isolation. Ann N Y Acad Sci 2011;1231:17-22.

12. Kobayashi LC, Steptoe A. Social Isolation, Loneliness, and Health Behaviors at Older Ages: Longitudinal Cohort Study. Ann Behav Med 2018;52:582-593.

13. Deckers K, van Boxtel MP, Schiepers OJ, et al. Target risk factors for dementia prevention: a systematic review and Delphi consensus study on the evidence from observational studies. Int J Geriatr Psychiatry 2015;30:234-246.

14. Killin LOJ, Starr JM, Shiue IJ, Russ TC. Environmental risk factors for dementia: a systematic review. BMC Geriatrics 2016;16:175.

15. Kunkle BW, Grenier-Boley B, Sims R, et al. Genetic meta-analysis of diagnosed Alzheimer's disease identifies new risk loci and implicates Aβ, tau, immunity and lipid processing. Nature genetics 2019;51:414-430.

16. van der Lee SJ, Wolters FJ, Ikram MK, et al. The effect of APOE and other common genetic variants on the onset of Alzheimer's disease and dementia: a community-based cohort study. Lancet neurol 2018;17:434-444.

17. Bateman RJ, Aisen PS, De Strooper B, et al. Autosomal-dominant Alzheimer's disease: a review and proposal for the prevention of Alzheimer's disease. Alzheimer's research & therapy 2011;3:1-1.

18. Rossor MN, Fox NC, Mummery CJ, Schott JM, Warren JD. The diagnosis of young-onset dementia. Lancet neurol 2010;9:793-806.

19. Ryman DC, Acosta-Baena N, Aisen PS, et al. Symptom onset in autosomal dominant Alzheimer disease: a systematic review and meta-analysis. Neurology 2014;83:253-260.

20. Reiman EM, Arboleda-Velasquez JF, Quiroz YT, et al. Exceptionally low likelihood of Alzheimer's dementia in APOE2 homozygotes from a 5,000-person neuropathological study. Nature Communications 2020;11:667.

21. Genin E, Hannequin D, Wallon D, et al. APOE and Alzheimer disease: a major gene with semi-dominant inheritance. Mol Psychiatry 2011;16:903-907.

22. Myers RH, Schaefer EJ, Wilson PW, et al. Apolipoprotein E epsilon4 association with dementia in a population-based study: The Framingham study. Neurology 1996;46:673-677.

23. Slooter AJ, Cruts M, Kalmijn S, et al. Risk estimates of dementia by apolipoprotein E genotypes from a population-based incidence study: the Rotterdam Study. Archives of neurology 1998;55:964-968.

24. Ngandu T, Lehtisalo J, Solomon A, et al. A 2 year multidomain intervention of diet, exercise, cognitive training, and vascular risk monitoring versus control to prevent cognitive decline in at-risk elderly people (FINGER): a randomised controlled trial. Lancet 2015;385:2255-2263.

25. Solomon A, Turunen H, Ngandu T, et al. Effect of the Apolipoprotein E Genotype on Cognitive Change During a Multidomain Lifestyle Intervention: A Subgroup Analysis of a Randomized Clinical Trial. JAMA Neurol 2018;75:462-470.

26. Lourida I, Hannon E, Littlejohns TJ, et al. Association of Lifestyle and Genetic Risk With Incidence of Dementia. JAMA 2019;322:430-437.

27. Licher S, Ahmad S, Karamujić-Čomić H, et al. Genetic predisposition, modifiable-risk-factor profile and long-term dementia risk in the general population. Nature medicine 2019;25:1364-1369.

28. Sharp ES, Gatz M. Relationship between education and dementia: an updated systematic review. Alzheimer Disease & Associated Disorders 2011;25:289-304.

29. Corrigan JD, Bogner J. Initial reliability and validity of the Ohio State University TBI Identification Method. J Head Trauma Rehabil 2007;22:318-329.

30. Williams B, Mancia G, Spiering W, et al. 2018 ESC/ESH Guidelines for the management of arterial hypertension. Eur Heart J 2018;39:3021-3104.

31. McKenna H, Treanor C, O'Reilly D, Donnelly M. Evaluation of the psychometric properties of self-reported measures of alcohol consumption: a COSMIN systematic review. Subst Abuse Treat Prev Policy 2018;13:6.

32. Razi S, Manish G, Keshav G, Sukriti K, Gupta A. Site or Size of Waist Circumference, Which one is More important in Metabolic Syndrome? International Journal of Medicine and Public Health 2016;6:69-72.

33. Lycke M, Lefebvre T, Cool L, et al. Screening Methods for Age-Related Hearing Loss in Older Patients with Cancer: A Review of the Literature. Geriatrics (Basel) 2018;3:48.

34. Cosentino F, Grant PJ, Aboyans V, et al. 2019 ESC Guidelines on diabetes, pre-diabetes, and cardiovascular diseases developed in collaboration with the EASD. Eur Heart J 2020;41:255-323.

35. Levis B, Sun Y, He C, et al. Accuracy of the PHQ-2 Alone and in Combination With the PHQ-9 for Screening to Detect Major Depression: Systematic Review and Meta-analysis. JAMA 2020;323:2290-2300.

36. Lubben JE. Assessing social networks among elderly populations. Family & Community Health 1988;11.

37. Koenig HG, Westlund RE, George LK, Hughes DC, Blazer DG, Hybels C. Abbreviating the Duke Social Support Index for Use in Chronically Ill Elderly Individuals. Psychosomatics 1993;34:61-69.

38. Sylvia LG, Bernstein EE, Hubbard JL, Keating L, Anderson EJ. Practical guide to measuring physical activity. J Acad Nutr Diet 2014;114:199-208.

39. Shaw LM, Arias J, Blennow K, et al. Appropriate use criteria for lumbar puncture and cerebrospinal fluid testing in the diagnosis of Alzheimer's disease. Alzheimers Dement 2018;14:1505-1521.

40. Janelidze S, Stomrud E, Palmqvist S, et al. Plasma beta-amyloid in Alzheimer's disease and vascular disease. Scientific reports 2016;6:26801.

41. Palmqvist S, Janelidze S, Stomrud E, et al. Performance of Fully Automated Plasma Assays as Screening Tests for Alzheimer Disease-Related beta-Amyloid Status. JAMA Neurol 2019.

42. Janelidze S, Mattsson N, Palmqvist S, et al. Plasma P-tau181 in Alzheimer's disease: relationship to other biomarkers, differential diagnosis, neuropathology and longitudinal progression to Alzheimer's dementia. Nature medicine 2020;26:379-386.

43. Thijssen EH, La Joie R, Wolf A, et al. Diagnostic value of plasma phosphorylated tau181 in Alzheimer's disease and frontotemporal lobar degeneration. Nature medicine 2020;26:387-397.

44. Karikari TK, Pascoal TA, Ashton NJ, et al. Blood phosphorylated tau 181 as a biomarker for Alzheimer's disease: a diagnostic performance and prediction modelling study using data from four prospective cohorts. Lancet neurol 2020;19:422-433.

45. Palmqvist S, Janelidze S, Quiroz YT, et al. Discriminative Accuracy of Plasma Phospho-tau217 for Alzheimer Disease vs Other Neurodegenerative Disorders. JAMA 2020.

46. Lewczuk P, Riederer P, O'Bryant SE, et al. Cerebrospinal fluid and blood biomarkers for neurodegenerative dementias: An update of the Consensus of the Task Force on Biological Markers in Psychiatry of the World Federation of Societies of Biological Psychiatry. World J Biol Psychiatry 2018;19:244-328.

47. Mattsson N, Andreasson U, Zetterberg H, Blennow K, Alzheimer's Disease Neuroimaging I. Association of Plasma Neurofilament Light With Neurodegeneration in Patients With Alzheimer Disease. JAMA Neurol 2017.

48. Weston PSJ, Poole T, Ryan NS, et al. Serum neurofilament light in familial Alzheimer disease: A marker of early neurodegeneration. Neurology 2017;89:2167-2175.

49. Preische O, Schultz SA, Apel A, et al. Serum neurofilament dynamics predicts neurodegeneration and clinical progression in presymptomatic Alzheimer's disease. Nature medicine 2019;25:277-283.

50. Chhatwal JP, Schultz AP, Dang Y, et al. Plasma N-terminal tau fragment levels predict future cognitive decline and neurodegeneration in healthy elderly individuals. Nature Communications 2020;11:6024.

51. Cullen NC, Leuzy A, Palmqvist S, et al. Individualized prognosis of cognitive decline and dementia in mild cognitive impairment based on plasma biomarker combinations. Nature Aging 2020.

52. Desikan RS, Fan CC, Wang Y, et al. Genetic assessment of age-associated Alzheimer disease risk: Development and validation of a polygenic hazard score. PLoS Med 2017;14:e1002258.

53. Escott-Price V, Sims R, Bannister C, et al. Common polygenic variation enhances risk prediction for Alzheimer's disease. Brain 2015;138:3673-3684.

54. Tan CH, Bonham LW, Fan CC, et al. Polygenic hazard score, amyloid deposition and Alzheimer's neurodegeneration. Brain 2019;142:460-470.

55. Holstege H, van der Lee SJ, Hulsman M, et al. Characterization of pathogenic SORL1 genetic variants for association with Alzheimer's disease: a clinical interpretation strategy. Eur J Hum Genet 2017;25:973-981.

56. Bateman RJ, Xiong C, Benzinger TLS, et al. Clinical and Biomarker Changes in Dominantly Inherited Alzheimer's Disease. New England Journal of Medicine 2012;367:795-804.

57. Rohrer JD, Nicholas JM, Cash DM, et al. Presymptomatic cognitive and neuroanatomical changes in genetic frontotemporal dementia in the Genetic Frontotemporal dementia Initiative (GENFI) study : a cross-sectional analysis. Lancet neurology 2015;14:253-262.

58. Schott JM, Fox NC, Frost C, et al. Assessing the onset of structural change in familial Alzheimer's disease. Annals of neurology 2003;53:181-188.

59. Fox NC, Freeborough PA, Rossor MN. Visualisation and quantification of rates of atrophy in Alzheimer's disease. The Lancet 1996;348:94-97.

60. Frisoni GB, Fox NC, Jack CR, Scheltens P, Thompson PM. The clinical use of structural MRI in Alzheimer disease. Nat Rev Neurol 2010;6:67-77.

61. Hill DLG, Schwarz AJ, Isaac M, et al. Coalition Against Major Diseases/European Medicines Agency biomarker qualification of hippocampal volume for enrichment of clinical trials in predementia stages of Alzheimer's disease. Alzheimers Dement 2014;10:421-429.e423.

62. Lombardi G, Crescioli G, Cavedo E, et al. Structural magnetic resonance imaging for the early diagnosis of dementia due to Alzheimer's disease in people with mild cognitive impairment. Cochrane Database of Systematic Reviews 2020.

63. Rittman T. Neurological update: neuroimaging in dementia. Journal of Neurology 2020;267:3429-3435.

64. Veldsman M, Tai X-Y, Nichols T, et al. Cerebrovascular risk factors impact frontoparietal network integrity and executive function in healthy ageing. Nature Communications 2020;11:4340.

65. Wardlaw JM, Smith EE, Biessels GJ, et al. Neuroimaging standards for research into small vessel disease and its contribution to ageing and neurodegeneration. The Lancet Neurology 2013;12:822-838.

66. Debette S, Markus HS. The clinical importance of white matter hyperintensities on brain magnetic resonance imaging: systematic review and meta-analysis. BMJ 2010;341:c3666.

67. Inzitari D, Pracucci G, Poggesi A, et al. Changes in white matter as determinant of global functional decline in older independent outpatients: three year follow-up of LADIS (leukoaraiosis and disability) study cohort. Bmj 2009;339:b2477.

68. Kuźma E, Lourida I, Moore SF, Levine DA, Ukoumunne OC, Llewellyn DJ. Stroke and dementia risk: A systematic review and meta-analysis. Alzheimers Dement 2018;14:1416-1426.

69. Bos D, Wolters FJ, Darweesh SKL, et al. Cerebral small vessel disease and the risk of dementia: A systematic review and meta-analysis of population-based evidence. Alzheimers Dement 2018;14:1482-1492.

70. Scheltens P, Leys D, Barkhof F, et al. Atrophy of medial temporal lobes on MRI in "probable" Alzheimer's disease and normal ageing: diagnostic value and neuropsychological correlates. Journal of neurology, neurosurgery, and psychiatry 1992;55:967-972.

71. Koedam EL, Lehmann M, van der Flier WM, et al. Visual assessment of posterior atrophy development of a MRI rating scale. Eur Radiol 2011;21:2618-2625.

72. Pasquier F, Leys D, Weerts JG, Mounier-Vehier F, Barkhof F, Scheltens P. Inter- and intraobserver reproducibility of cerebral atrophy assessment on MRI scans with hemispheric infarcts. Eur Neurol 1996;36:268-272.

73. Wahlund LO, Barkhof F, Fazekas F, et al. A new rating scale for age-related white matter changes applicable to MRI and CT. Stroke 2001;32:1318-1322.

74. Vernooij MW, Pizzini FB, Schmidt R, et al. Dementia imaging in clinical practice: a European-wide survey of 193 centres and conclusions by the ESNR working group. Neuroradiology 2019;61:633-642.

75. Fazekas F, Chawluk JB, Alavi A, Hurtig HI, Zimmerman RA. MR signal abnormalities at 1.5 T in Alzheimer's dementia and normal aging. AJR Am J Roentgenol 1987;149:351-356.

76. Dickerson BC, Bakkour A, Salat DH, et al. The cortical signature of Alzheimer's disease: regionally specific cortical thinning relates to symptom severity in very mild to mild AD dementia and is detectable in asymptomatic amyloid-positive individuals. Cereb Cortex 2009;19:497-510.

77. Giorgio J, Landau SM, Jagust WJ, Tino P, Kourtzi Z, Alzheimer's Disease Neuroimaging I. Modelling prognostic trajectories of cognitive decline due to Alzheimer's disease. Neuroimage Clin 2020;26:102199-102199.

78. Archetti D, Ingala S, Venkatraghavan V, et al. Multi-study validation of data-driven disease progression models to characterize evolution of biomarkers in Alzheimer's disease. NeuroImage: Clinical 2019;24:101954.

79. Fisher CK, Smith AM, Walsh JR. Machine learning for comprehensive forecasting of Alzheimer’s Disease progression. Scientific Reports 2019;9:1-14.

80. Pilotto A, Premi E, Paola Caminiti S, et al. Single-subject SPM FDG-PET patterns predict risk of dementia progression in Parkinson disease. Neurology 2018;90:e1029-e1037.

81. Rowe CC, Ellis KA, Rimajova M, et al. Amyloid imaging results from the Australian Imaging, Biomarkers and Lifestyle (AIBL) study of aging. Neurobiology of aging 2010;31:1275-1283.

82. Lowe VJ, Curran G, Fang P, et al. An autoradiographic evaluation of AV-1451 Tau PET in dementia. Acta Neuropathologica Communications 2016;4:58.

83. Endres CJ, Pomper MG, James M, et al. Initial Evaluation of 11C-DPA-713, a Novel TSPO PET Ligand, in Humans. Journal of Nuclear Medicine 2009;50:1276-1282.

84. Nabulsi NB, Mercier J, Holden D, et al. Synthesis and Preclinical Evaluation of 11C-UCB-J as a PET Tracer for Imaging the Synaptic Vesicle Glycoprotein 2A in the Brain. Journal of Nuclear Medicine: Official Publication, Society of Nuclear Medicine 2016;57:777-784.

85. Hou XH, Feng L, Zhang C, Cao XP, Tan L, Yu JT. Models for predicting risk of dementia: a systematic review. Journal of neurology, neurosurgery, and psychiatry 2019;90:373-379.

86. Tang EY, Harrison SL, Errington L, et al. Current Developments in Dementia Risk Prediction Modelling: An Updated Systematic Review. PloS one 2015;10:e0136181.

87. Kivipelto M, Ngandu T, Laatikainen T, Winblad B, Soininen H, Tuomilehto J. Risk score for the prediction of dementia risk in 20 years among middle aged people: a longitudinal, population-based study. Lancet neurol 2006;5:735-741.

88. Anstey KJ, Cherbuin N, Herath PM. Development of a new method for assessing global risk of Alzheimer's disease for use in population health approaches to prevention. Prevention science : the official journal of the Society for Prevention Research 2013;14:411-421.

89. Anstey KJ, Cherbuin N, Herath PM, et al. A self-report risk index to predict occurrence of dementia in three independent cohorts of older adults: the ANU-ADRI. PloS one 2014;9:e86141.

90. Barnes DE, Beiser AS, Lee A, et al. Development and validation of a brief dementia screening indicator for primary care. Alzheimers Dement 2014;10:656-665.e651.

91. Exalto LG, Biessels GJ, Karter AJ, et al. Risk score for prediction of 10 year dementia risk in individuals with type 2 diabetes: a cohort study. The Lancet Diabetes and Endocrinology 2013.

92. Tang EYH, Price CI, Robinson L, et al. Assessing the Predictive Validity of Simple Dementia Risk Models in Harmonised Stroke Cohorts. Stroke 2020;51 (7):2095-2102.

93. Stephan BCM, Pakpahan E, Siervo M, et al. Prediction of dementia risk in low-income and middle-income countries (the 10/66 Study): an independent external validation of existing models. Lancet Glob Health 2020;8:e524-e535.

Figure 1. Frequent and rare variants in Alzheimer's disease.

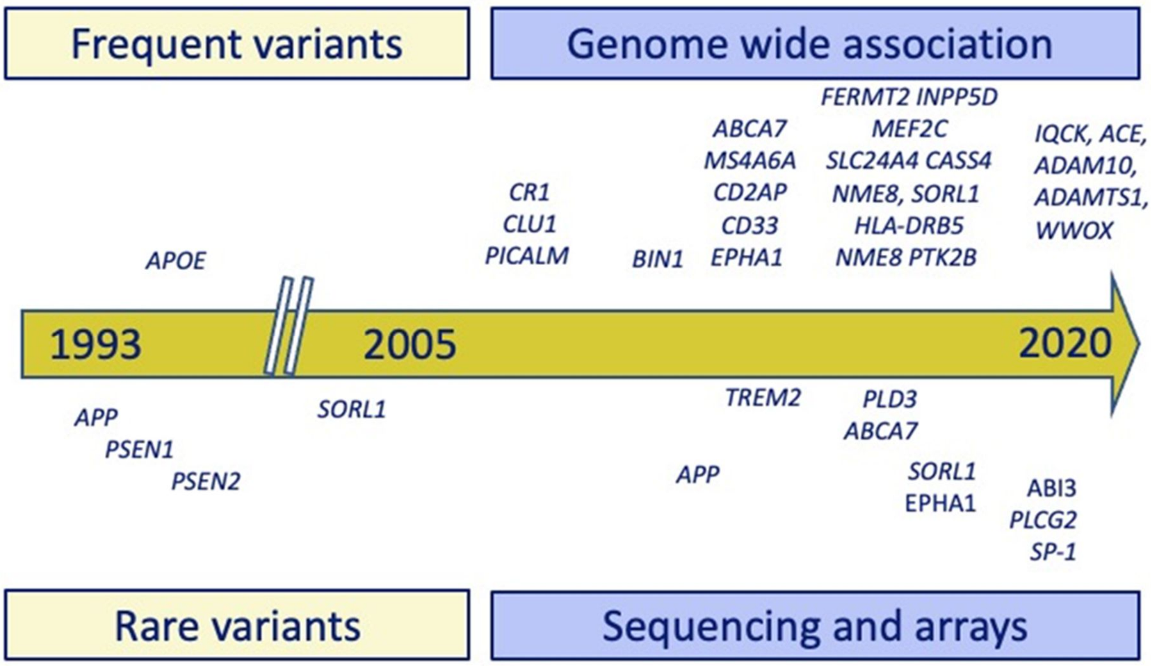

Table 1. Assessment of potentially modifiable risk factors in Brain Health Services.

**Risk factor**	**Assessment methods**
Education	- International Standard Classification of Education (*applicable across educational systems*)[28]<br>- Years of education (*simple to calculate*)[28]
Lifetime traumatic brain injury	- Ohio State University Traumatic Brain Injury Identification Method (*ideal*)[29]<br>- Medical history or informant or self-reported reports (*practical*)
Hypertension	- Ambulatory devices (*ideal*)<br>- Domestic device (*practical*)<br>Defined as in-office measures at 140/90 and lower in ambulatory or home-based assessments[30].
Alcohol consumption	- Quantity-frequency measures with beverage-specific assessment of time frames and binge-drinking episodes[31] (*ideal*)<br>- >21 units per week to define high risk (*more practical*)
Obesity and visceral adipose tissue	- Waist circumference (*ideal*)<br>- Body mass index (*practical*)<br>*Note*: There are different ways to measure waist circumference and different cut-offs depending on ethnicity and world region[32].
Hearing impairment	- Pure tone audiometry[33] (*gold standard*)<br>- Whispered Voice Test (*simple but less reliable*)<br>- Speech-in-noise paradigms (*simple but less reliable*)<br>- Questionnaires (*less reliable*)
Diabetes	- Fasting plasma glucose levels (>=7.0mmol/l) or HbA1c (>=6.5%)<br>- Oral glucose tolerance test to diagnose impaired glucose tolerance[34].
Smoking	- Pack years (number of daily packs multiplied by number of years smoking)<br>- Current smoking status (current versus former/never smoker)
Air pollution	- Further research is needed to establish a practical clinically-relevant measure.
Depression	- Depression screening measures e.g. Patient-Health-Questionnaire (PHQ)[35].
Social isolation	- Short questionnaires, e.g. the Lubben Social Network Scale[36] or the Duke Social Support Index[37].
Physical inactivity	- Accelerometers[38]<br>- Heart rate counters[38]<br>- Smart phone or smart watch apps[38]<br>- Self-reported measures (*more practical for clinical setting)*

Table 2. Comparison of Selected Dementia Risk Models.

	**Cardiovascular Risk Factors, Aging and Dementia (CAIDE) Score**	**Australian National University Alzheimer's Disease Risk Index (ANU-ADRI)**	**Brief Dementia Screening Indicator (BDSI)**
Development sample age range	39-64	Variable (population based)	65+
Development sample size	1,409	903 – 2496	1125 - 13889
Mean length of follow-up, years	21	Variable (population based)	6
Accuracy (area under the curve or C-statistic)**	0.77 – 0.78	0.64-0.74	0.68-0.78
Demographic predictors			
Age	●	●	●
Sex	●	●	
Education*	●	●	●
Functional Impairment			
Difficulty with instrumental activities of daily living			●
Health predictors			
Systolic blood pressure*	●		
Body mass index*	●		●
Total cholesterol	●		
Diabetes*		●	●
Stroke			●
Traumatic brain injury*		●	
Depression*/Depressive symptoms		●	●
Lifestyle predictors			
Smoking*		●	
Physical activity*	●	●	
Social isolation*		●	
Cognitively stimulating activities		●	
Alcohol*		●	
Fish intake		●	
Genetic predictors			
APOE e4 carrier	●		

* Modifiable risk factor identified in the 2020 Report of the *Lancet* Commission[3].

**Range includes the development and validation test results.

Figure 2. Proposed workflow for dementia risk profiling in BHSs.

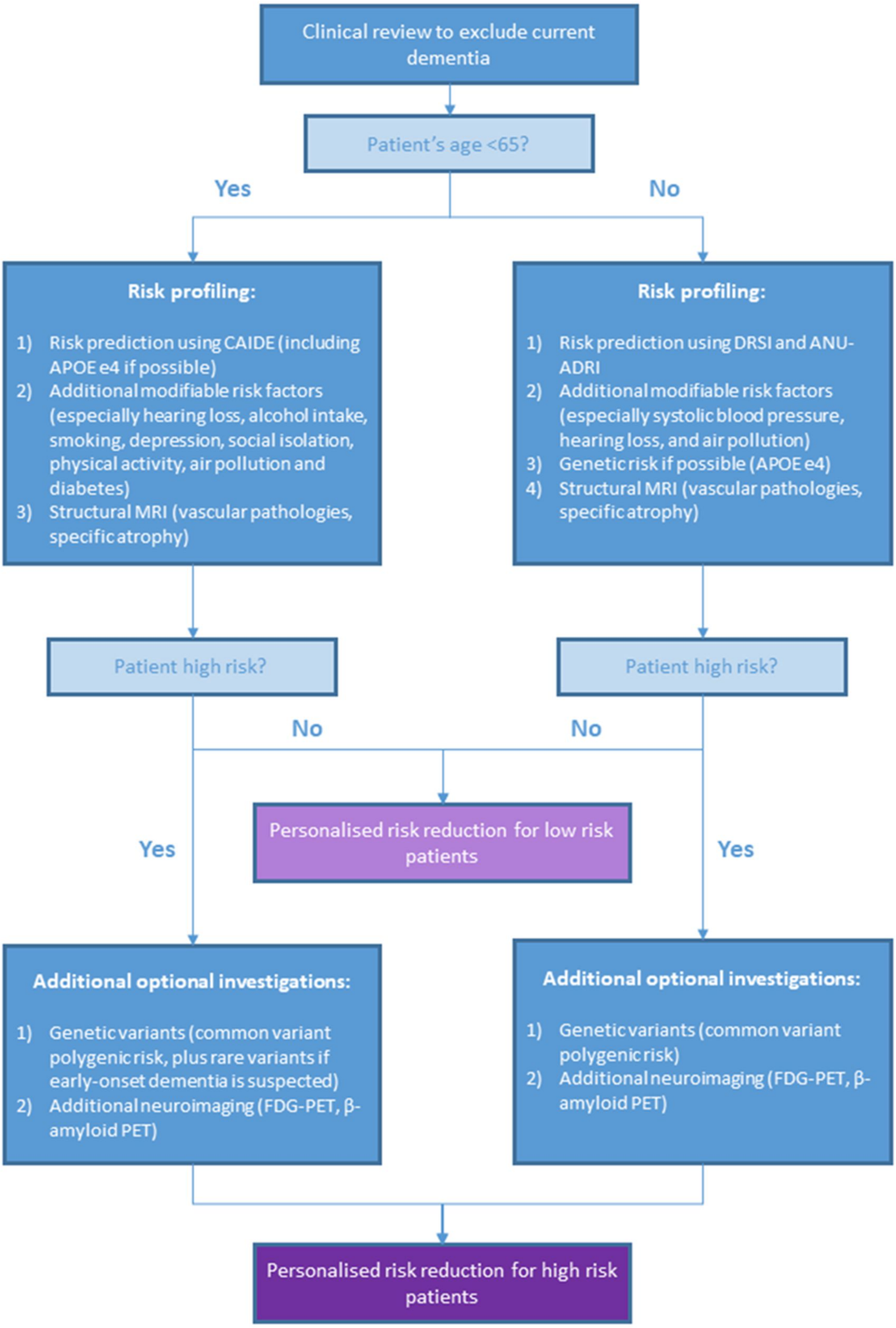

Dementia risk communication.

A user manual for Brain Health Services – Part 3 of 6

Leonie N.C. Visser*, PhD[1,2]; Carolina Minguillon*, PhD[3,4,5]; Gonzalo Sánchez-Benavides, PhD[3,4,5]; Marc Abramowicz, MD, PhD[6]; Daniele Altomare, PhD[7,8]; Karine Fauria, PhD[3,4]; Giovanni B. Frisoni, MD[7,8]; Jean Georges, BA[9]; Federica Ribaldi, MS[6,7,10,11]; Philip Scheltens, MD, PhD[1]; Jetske van der Schaar, MA[1]; Marissa Zwan, PhD[1]; Wiesje M. van der Flier+, PhD[1,12]; José Luis Molinuevo+, MD, PhD[3] *on behalf of the European Task Force for Brain Health Services*

*These authors contributed equally to this work (shared first author).

+These authors contributed equally to this work (shared last author).

[1]Alzheimer Center Amsterdam, Department of Neurology, Amsterdam Neuroscience, Vrije Universiteit Amsterdam, Amsterdam UMC, Amsterdam, The Netherlands.

[2]Center for Alzheimer Research, Division of Clinical Geriatrics, Department of Neurobiology, Care Sciences and Society, Karolinska Institutet, Solna, Sweden.

[3]Barcelonaβeta Brain Research Center (BBRC), Pasqual Maragall Foundation, Barcelona, Spain.

[4]IMIM (Hospital del Mar Medical Research Institute), Barcelona, Spain.

[5]Centro de Investigación Biomédica en Red de Fragilidad y Envejecimiento Saludable (CIBERFES), Madrid, Spain.

[6]Division of Genetic Medicine, Department of Diagnostics, Geneva University Hospitals and University of Geneva, Switzerland.

[7]Laboratory of Neuroimaging of Aging (LANVIE), University of Geneva, Geneva, Switzerland.

[8]Memory Clinic, Geneva University Hospitals, Geneva, Switzerland.

[9]Alzheimer Europe, Luxembourg.

[10]Laboratory of Alzheimer's Neuroimaging and Epidemiology (LANE), Saint John of God Clinical Research Centre, Brescia, Italy.

[11]Department of Molecular and Translational Medicine, University of Brescia, Brescia, Italy.

[12]Department of Epidemiology and Data Science, Vrije Universiteit Amsterdam, Amsterdam UMC, Amsterdam, The Netherlands.

Corresponding author(s)

Leonie (N.C.) Visser, PhD

Karolinska Institutet | Dep. of Neurobiology, Care Sciences and Society (NVS) | Division of Clinical Geriatrics | Center for Alzheimer Research

Karolinska vägen 37 A, QA32, 171 64 Solna, Sweden

Email: leonie.visser@ki.se.

Carolina Minguillon, PhD

Barcelonaβeta Brain Research Center

Wellington, 30. 08005 Barcelona, Spain

Email: cminguillon@barcelonabeta.org.

MANUSCRIPT DETAILS

Character count title (with spaces): 81.

Word count abstract: 319.

Word count manuscript: 4089.

Number of references: 78.

Number of figures: 1.

Number of tables: 1.

Number of boxes: 2.

Supplementary Material: A and B.

ABSTRACT

Growing evidence suggests dementia incidence can be reduced through prevention programs targeting risk factors. To accelerate implementation of such prevention programs, a new generation of brain health services (BHS) is envisioned, involving risk profiling, risk communication, risk reduction and cognitive enhancement. The purpose of risk communication is to enable individuals at risk to make informed decisions and take action to protect themselves, and is thus a crucial step in tailored dementia prevention strategies. However, communication about dementia risk is complex and challenging.

In this paper, we provide an overview of: i) perspectives on communicating dementia risk from an ethical, clinical and societal viewpoint, ii) insights gained from practice in memory clinics, iii) available evidence on the impact of disclosing *APOE* and Alzheimer's disease biomarker test results gathered from clinical trials and observational studies, iv) the value of established registries in light of BHS, and v) practical recommendations regarding effective strategies for communication about dementia risk.

In addition, we identify challenges, *i.e.*, the current lack of evidence on *what* to tell on an individual level -the actual risk-, and on *how* to optimally communicate about dementia risk, especially concerning worried yet cognitively unimpaired individuals. Ideally, dementia risk communication strategies should maximize the desired impact of risk information on individuals' understanding of their health/disease status and risk perception, and minimize potential harms. More research is thus warranted on the impact of dementia risk communication, to i) evaluate the merits of different approaches to risk communication on outcomes in the cognitive, affective and behavioral domains, ii) develop an evidence-based, harmonized dementia risk communication protocol, and iii) develop e-tools to support and promote adherence to this protocol in BHSs.

Based on the research reviewed, we recommend that dementia risk communication should be precise, include the use of absolute risks, visual displays and time frames, be based on a process of shared decision making, and address the inherent uncertainty that comes with any probability.

Keywords

Brain Health Services; dementia; aging; Alzheimer’s disease; prevention; risk communication

1. BACKGROUND

Encouraging evidence suggests dementia incidence can be reduced by means of precision prevention programs targeting risk factors [3, 4]. This entails tailoring risk reduction to the clinical, biological, genetic, and psychosocial characteristics of each patient. To accelerate implementation of such a precision approach in dementia prevention, a new generation of Brain Health Services (BHSs) can be envisioned (Altomare et al., *this issue*), guided by risk profiling (Ranson et al., *this issue*) and risk communication (the present paper), with the general goal of personalized risk reduction (Solomon et al., *this issue*) and cognitive enhancement (Brioschi et al., *this issue*), in an ethical and equitable context (Milne et al., *this issue*).

The recently proposed diagnostic framework for Alzheimer's disease (AD) sets the biologically defined disease (by amyloid-β and tau) apart from the clinical staging [5]. This underlines that it is pivotal to define which risk one is referring to, since 'risk' may refer to an individual's likelihood of getting a disease such AD, which does not immediately imply a clinical outcome (here, cognitive impairment or dementia). Yet, 'risk' may also refer to the likelihood of developing the bad, clinical outcome (*i.e.* dementia). Here, we focus mainly on the dementia risk.

The World Health Organization defines risk communication as 'an exchange of real-time information, advice and opinions between experts and people facing threats to their health, economic or social well-being'[6]. The purpose of risk communication is to enable individuals to make informed decisions and take action to protect themselves. Because of the influence that perceptions of individualized risk are expected to have on people's health- and disease-related behavior, the provision of risk information is an essential aspect of any health prevention effort [7]. Thus, risk communication is a crucial step in tailored prevention strategies of dementia incidence.

However, communicating risk is complex and therefore challenging for clinicians [8, 9]. First, the concept of risk is difficult for patients to comprehend and for physicians to explain [9-14]. Second, risk disclosure is an intervention by itself, because of its potential impact on psychological and mental health of the individual[15]. Still, knowing your individual potential for prevention and being able to take action through personalized, multi-domain, risk reduction prevention programs are considered important benefits [16, 17].

How to communicate dementia risk depends on the context. Within the context of trial enrollment, disclosure of biomarker evidence and hence dementia risk can be warranted and risk disclosure is then embedded in the protocol [14]. In the clinical context, increasing numbers of individuals without cognitive impairment are seeking care [18, 19], and express a need for information, guidance and practical advice, although they vary in specific information needs and preferences [20-23]. The envisioned BHSs are aimed at providing meaningful answers to this growing demand. These individuals experience subjective cognitive decline (SCD)[24] or functional cognitive disorders[19], or are just concerned about cognitive decline and/or their brain health and want to preserve their cognitive performance as long as possible. These individuals represent the target population of BHSs (Altomare et al., *this issue*), and could be subjected to prediction modeling to inform their individualized dementia risk, although these models are not perfect and risk communication remains challenging [25].

To this background, communication strategies should maximize the desired impact of risk information on individuals' understanding of their health/disease status and dementia risk perception, and minimize potential harms. This paper aims to provide an overview of different perspectives, available evidence, and practice recommendations regarding optimal strategies for communicating dementia risk, and identify next steps in the development of an evidence-based risk communication protocol.

2. PERSPECTIVES ON COMMUNICATING DEMENTIA RISK

2.1. Ethical perspective

Individuals have a moral and legal right to know or not know their dementia risk [26]. Whether someone wants to know and how it impacts them is very personal. As one researcher stated: "Depending on the person's temperament, it could ruin their life or it could help them along" [27]. Among potential personal benefits are a reduction in feelings of uncertainty and anxiety, enhanced preparedness for the future and improved quality of life [28-31]. On the other hand, facing a high probability to develop dementia can have negative psychological effects, including stress, depression or even suicidal ideation, and affect sense of self, future and perception of memory [28, 32]. In addition, sharing personal risk information with others could lead to stigmatization as well as social, professional and legal discrimination. While (inter)national agreements protect genetic privacy and prohibit discrimination, these may not apply to biomarker-based risk, nor address protections for long-term care insurance[33]. Nonetheless, from an ethical perspective it is questionable whether these are reasons *not* to communicate dementia risk, especially when individuals prefer to know.

Another important argument in the context of provision of dementia risk information is actionability. While some argue that there may be a lack of actionability in the absence of a disease-modifying treatment for AD [15, 34], others point out that learning their likelihood of developing dementia empowers individuals to shift priorities or try to reduce risk through (other) preventive actions, for example controlling modifiable risk factors such as hypertension [3], or adopting a healthier lifestyle [28, 35]. Knowing ones' risk could thus meaningfully contribute to a morally worthwhile set of options. It is important to educate, prepare and counsel individuals on all that is known and still uncertain[36], so they can make informed decisions about knowing their dementia risk and utilize their right to self-determination, while they still can.

2.2 Clinician perspective

When facing a risk, proper decision making by the individual about preventive/protective action requires proper understanding and conceptualization of this risk. Clinicians must explain the medical problem (*i.e.*, dementia), the magnitude of the risk and its implications in a way that is readily understandable by lay people. Here, we can learn from cancer genetics, with a longer history in risk communication.

In Mendelian, autosomal dominant conditions with full penetrance, the risk of developing the disease corresponds to the risk of inheriting the mutation, *i.e.*, 50% for each child of an affected patient, which is easily understood as the outcome of flipping a coin. This applies to some Mendelian forms of AD (e.g., *Presenilin* mutations). Conveying a risk becomes slightly more complex when penetrance is incomplete (e.g., *BRCA* mutations), say 70%, producing a disease risk of 35%. Here, geneticists will convey a finer analogy, like picking one out of a hundred marbles from a bag, 65 being green (no disease) and 35 red (disease). Next are moderate-penetrance genes like *CHEK2*, causing a significant but limited increase in breast cancer risk. This compares with some moderately penetrant Alzheimer's genes, including *APOE-ε4*. In families with many affected, other factors than the *CHEK2* mutation are also at play. Hence, pre-symptomatic testing in unaffected at-risk relatives might carry false alarm if the mutation is found to be present (because the associated cancer risk is limited), and false reassurance if the mutation is found to be absent (because the residual risk remains increased over population risk)[37]. The analogy of picking one out of a hundred marbles with different colors could also be used to explain the risk of developing dementia associated with moderately penetrant Alzheimer's genes, including these false cases.

In hereditary breast and ovarian cancer, pre-symptomatic genetic testing allows for targeted prevention[38]. However, depending on the *magnitude* of the risk, one might weigh potential benefits and harms of preventive options differently. For example, considering preventive mastectomy with a *BRCA1* mutation versus *CHEK2*. This highlights how effective risk communication is essential, especially when weighing pros and cons of preventive options. With the advent of effective pre-symptomatic strategies in AD, we should learn from the experience learned in genetic counseling in cancer.

2.3 Societal/public perspective

AD is a major healthcare concern for many people [39]. Having been asked to choose the one disease they were most afraid of from a list of seven, respondents in a public opinion survey most frequently identified cancer, followed by AD (about one quarter). Interestingly, there was a considerable public interest in pre-symptomatic diagnostic testing. Asked individuals whether they want to take a medical test which would tell them whether they would develop AD, a plurality in all countries responded that they would be 'very likely' or 'somewhat likely' to get such test, ranging from 51% in Germany to 78% in Poland. However, there's also evidence that people's preferences for knowing their AD status or dementia risk decrease when they have had the possibility to think about the consequences of receiving such information [40].

In light of the changing definitions of AD, which could lead to differences in understanding and miscommunication[41], special attention should be paid to raise awareness in the general public about the spectrum of AD from the asymptomatic to the dementia stage. Similarly, prevention messages should be included in campaigns targeting the general public and at-risk populations.

3. EVIDENCE ON COMMUNICATING DEMENTIA RISK

3.1. Memory clinic setting

People present at memory clinics with symptoms or problems in daily live. This is different from the envisioned BHSs mainly aimed at asymptomatic individuals. Yet, memory clinic experience, particularly with regards to MCI is informative for BHSs[42]. The recently published practice guideline by the American Academy of Neurology (AAN) states that an accurate diagnosis of MCI is important, especially to discuss the prognostic possibilities, *i.e.* risk of dementia [43]. Nonetheless, communicating the MCI label remains challenging for clinicians [8], and few provide specific or personalized information on the dementia risk to MCI patients [9].

One of the reasons for being reluctant to sharing prognostic information is the apparent lack of individualized risk information for MCI patients, leaving clinicians at best to provide patients with a 'fifty-fifty probability'. Recent evidence however illustrates that in MCI, prediction models with good accuracy, calibration and generalization allow an individualized prognosis based on biomarker evidence [44]. Even when these models still warrant prospective clinical validation, their clinical applicability is at the horizon. On a group level, biomarkers are also predictive of incident dementia in cognitively unimpaired individuals who present with SCD at the memory clinic, although it should be noted that at least half of biomarker positive individuals with SCD does not progress to dementia within 5 years[45]. For this population, individualized risk models are not yet ready for implementation, since their external validation is suboptimal [25]. In short, it is not easy to convert biomarker findings to an actual specific and personalized dementia risk. Clinicians' reluctance in sharing this information is therefore appropriate. To this background, considering the BHS target group of cognitively unimpaired individuals, it is questionable whether we currently have the data available to derive reliable dementia risk estimates. Risk communication can only be as good as the models it is based on, and clearly more work needs to be done.

3.2. Research setting

To identify individuals at higher risk of developing Alzheimer's disease (AD) or dementia, *APOLIPOPROTEIN (APOE)* and amyloid Positron Emission Tomography (PET) testing are being incorporated in clinical trials and prospective studies as screening tools (e.g. Alzheimer's Prevention Initiative Generation Program[46]; Anti-Amyloid Treatment in Asymptomatic Alzheimer's Disease (A4) Study[47]; AMYPAD Diagnostic and Patient Management Study[48]). Although the disclosure of *APOE-ε4* carriership and amyloid PET positivity have distinct implications, trial protocols have adopted similar recommendations regarding the disclosure of *APOE* and amyloid PET test results (Supplement A in Supplementary Material).

A number of studies have been carried out to assess the safety of disclosing AD biomarker test results to cognitively unimpaired individuals in the context of trials (Table 1). Most research has shown that disclosure of Alzheimer biomarker results does not lead to short-term negative psychological consequences (Table 1). However, it may not always be possible to extrapolate results from highly motivated individuals taking part in research to a more general population. Furthermore, none of the existing disclosure protocols have dealt with actual risk communication, which would imply the provision of the precise magnitude of the risk of developing dementia, considering a specific time-frame (e.g. risk within a number of years). Moreover, additional research is needed to examine the broader implications, both beneficial and harmful, of living with risk, and the impact over longer periods of time.

Suggested placement Table 1 (added at the end of this document).

3.3. Brain research registries

To foster enrollment in clinical trials, brain research registries may help. Here, we present three examples of registries, aiming to catalyze enrolment in studies, making the recruitment process more efficient. These registries closely align with the high interest in brain research and brain health in the general community.

3.3.1. The "BBDPS Study: a study on risk factor disclosure" and its associated registry

In 2018, the Barcelonaβeta Brain Research Center (BBRC) implemented the Barcelonaβeta Dementia Prevention Study (BBDPS), with its associated registry aiming at recruiting individuals with SCD or mild cognitive impairment (MCI) from the general population (detailed in Supplement B in Supplementary Material). People were invited to register if they "were feeling changes in their memory or cognitive status". The study's schematic is shown in Figure B1 in Supplementary Material. As of 30th June 2020, 306 persons have undergone the BBDPS baseline visit. From the ones with disclosed diagnosis (n=223), 17% were MCI and 57% SCD, meaning that nearly 75% of *a priori* selected individuals were corresponding to the population targeted. In total, 54% of these participants comply with eligibility criteria with ongoing studies at BBRC and 69% were actually enrolled. These numbers show the high efficiency of the registries' recruitment strategy, making the rate of screening failures much lower than standard rates, and demonstrate the value of the BBDPS-registry in light of BHSs since we offer ongoing (prevention) studies at BBRC to more than 50% of the pre-dementia individuals who are registered.

In BBDPS, we investigated the emotional impact of disclosing personal risk estimates by analysing depressive (Center for Epidemiologic Studies Depression Scale; CES-D[49]) and anxiety symptoms (State-Trait Anxiety Inventory; STAI[50]), as well as test-related distress (adapted from [51, 52]) in 128 BBDPS participants with SCD, with complete data up to the 6-month post-disclosure follow-up visit. From these, we first formed two extreme groups (n=69 with low risk as those with <4% risk of developing dementia in the next 5 years, n=20 with high risk as those with >10% risk). Group characteristics, including the mean dementia risk, are shown in Table B1 in Supplementary Material. As shown in Figure 1, no groups differences (low *vs* high risk) were found in depressive (A) and anxiety (B) symptoms or in test-related distress (C) across the study's time-points, indicating that disclosing 5-year dementia risk to cognitively unimpaired participants in a research setting is safe. We will further assess the longer-term safety in the whole BBDPS sample.

Suggested placement Figure 1 (added at the end of this document and as a separate file).

3.3.2. Brain Health Registry Switzerland

The Brain Health Registry Switzerland (www.bhr-suisse.org) aims to facilitate access to research programs to persons who wish to contribute to research on AD and memory-related diseases. The registry is open to all persons aged 50 and over. Once participants sign the informed consent, they are in the registry and can be contacted by researchers from one of the participating Swiss memory clinics (currently Geneva, Lausanne, Fribourg, St. Gallen or Lugano) and offered the opportunity to participate in a study. The website offers generic advice on lifestyles for a healthy brain, but it does not provide registrants with information on their personal dementia risk.

3.3.3. The Dutch Brain Research Registry

The Dutch Brain Research Registry (*Hersenonderzoek.nl* in Dutch)[53] was set up in 2017 with the aim to accelerate recruitment of participants for current and future clinical brain disease studies in the Netherlands. To date, over 20.000 participants signed up (58±11 years old, 78% female). Using their personal online portal, registrants provide demographic information, medical history, family history of dementia, medication and substance use and lifestyle information. Prescreening of registrants for studies/trials is solely based on this self-reported information. For this reason, the Dutch Brain Research Registry chose not to provide registrants with any information on their personal risk for developing AD and/or dementia.

Between January and June 2019, we organized five focus groups (each 3 to 8 participants, total n=28) to explore registrant experiences, including motivations for registration. We found that in addition to altruistic reasons (contribute to science/society) and family-related reasons (brain disease runs in family), registrants often reported that receiving information about their brain health and gaining insight in improving or maintaining their own brain health were important reasons for registration. These findings indicate that many of the registrants of a low-threshold, online research registry like the Dutch Brain Research Registry are representative of the target population for the envisioned BHSs.

4. PRACTICAL RECOMMENDATIONS ON RISK COMMUNICATION

4.1 How to communicate risk

Box 1 displays nine practical recommendations on how to communicate about dementia risk, as synthesized from available guidelines and evidence in the oncology field [11, 54, 55]. The online tool ADappt (www.ADappt.health) encompasses a dementia risk calculation tool, including a communication sheet taking into account these recommendations [56].

BOX 1. Practice recommendations for communicating risk in BHSs.

1. **Use plain language**, i.e. present focused, well-structured, and logically sequenced information, and reduce or eliminate clinical and statistical jargon.
2. **Avoid exclusive use of qualitative risk descriptors**, e.g. 'a high risk' or 'many people'.
3. **Present precise and defined risk information**, such as percentages e.g. '65 percent of individuals similar to you', or frequencies e.g. '65 out of 100 individuals like you'.
4. **Be aware that order and framing affect risk perception, therefore use mixed framing**, e.g. '35 out of 100 individuals will develop dementia in 3 years time (negative framing) and 65 out of 100 individuals will not develop dementia in 3 years time (positive framing)'.
5. **Use visuals in addition to numerical risks**, e.g. bar charts or icon arrays.

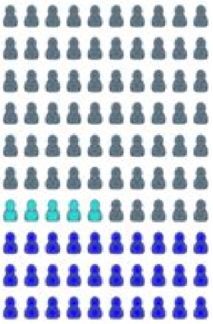

6. **Use an incremental risk format highlighting how an intervention changes risk**, e.g. by displaying the risk with and without intervention in the same icon array.
7. **Include and repeatedly draw attention to the time interval over which a risk occurs**, e.g. 'this graph displays the 5-year risk and this graph the lifetime risk of developing dementia'.
8. **Present absolute risks** (instead of relative), e.g. 'the lifetime dementia risk for people without hypertension is 25% compared to 35% for people with hypertension'.
9. **Do not use 'number needed to treat' since it reduces understanding,** e.g. 'if 33 individuals would be treated, 1 would not develop dementia because of the treatment'.

4.2 Managing expectations: communicating uncertainty & shared decision making

Risk profiling is about probabilistic statements regarding the individual's future, and therefore uncertainty is inherent [57]. Even when biomarker testing confirms a disease diagnosis, it often does not provide certainty about disease severity or its course [58]. Moreover, high-quality data are sometimes not available and even when they are, these data can have multiple interpretations or be contradictory, and risk models are never perfect [25]. Hence, communication about uncertainty is an integral aspect of risk disclosure, and managing expectations beforehand is important to inform individuals that this process will not necessarily result in a reduction of uncertainty [59]. An approach for uncertainty communication has been proposed [1], which comprises three steps presented in Box 2.

BOX 2. Recommendations on communicating uncertainty and shared decision making.

Communicating uncertainty – 3 steps [1]:

1. Normalizing uncertainty, by acknowledging individuals' wish for more certainty while explaining that uncertainty is unfortunately inherent to the situation;
2. Addressing the individual’s emotions regarding uncertainty by acknowledging that it is unpleasant not to know things;
3. Stimulating individuals to focus on living in the here and now instead of dwelling on the uncertainty, thereby helping them to cope with uncertainty.

Shared decision making – 4 steps [2]:

1. Informing the individual that a preference-sensitive decision is to be made;
2. Explaining the options including pros and cons;
3. Discussing what is important for the individual in his/her situation;
4. Discussing the individual’s preferred role in decision making, and make a decision.

Thus, risk communication is not simply about disclosing test results (*i.e.*, one-way information provision), but also about what and when individuals and their relatives want to know (*i.e.*, a two-way information exchange). Especially since individuals might weigh the potential harms and benefits of knowing their dementia risk differently, depending on their values, needs and situation [22]. A process of shared decision-making (SDM; Box 2)[2] offers a way to

incorporate evidence and the health care professional's expertise, as well as the individual's preferences into decision making about pursuing biomarker testing for AD and/or knowing their dementia risk [60, 61]. In general, the SDM approach has been shown to encourage health-promoting behaviours, reduce inappropriate or unnecessary use of care, and improve patient and clinician satisfaction [62, 63].

5. DISCUSSION

We reviewed and synthesized current evidence to formulate practice recommendations for communicating dementia risk. Based on evidence in the field of oncology, risk communication should be based on a process of shared decision making, taking into account that risk implies, by definition, uncertainty. Best practice recommendations for risk communication include the use of absolute risks, visual displays and time frames.

In memory clinics, patients present with signs and symptoms. Still, even in that context, there is room for improvement in risk communication [64]. Some experience is also gained in the trial setting, where protocols for disclosure of *APOE* and amyloid test results and dementia risk have been developed, and studies show that biomarker disclosure is not harmful in the short term [29, 65, 66]. But often this disclosure only infers the explanation that these biomarker findings *can be viewed as a risk factor* for dementia. They do not specify the time frame or the precise magnitude of the risk, nor do they relate this risk to the general risk of dementia (without knowledge on biomarkers) or the risk based on the individual's full risk profile (e.g., incorporating cardiovascular factors). Yet, an increasing number of individuals want to know their risk and specifically its meaning [22, 67, 68].

In other fields, such as oncology, quite a lot of research has been done on the optimal way to convey risks. A first attempt to integrate these recommendations in risk communication based on biomarker-findings in patients presenting at a memory clinic with MCI is www.ADappt.health [56]. However, risk communication is not only about *how* to communicate, but also about *what* to tell. The step to executing this type of risk communication in cognitively unimpaired individuals, the target population of BHSs, is quite large. Although findings at group level clearly endorse the predictive value of biomarker findings, these same findings also illustrate large heterogeneity in disease course, and therefore translation to the individual level is limited. It takes a long time from biomarker abnormality to onset of dementia, and this explains why large cohorts with long duration of follow-up are needed.

5.1. Challenges

Evidence about the effectivity of specific risk communication strategies mainly stems from data collected in other medical settings, such as oncology. Some important differences warrant

caution when translating that evidence to the field of AD and dementia. In oncology, patients with a given cancer diagnosis are presented with evidence-based treatment options and accompanying known probabilities of survival and other harms and benefits. In comparison, in AD, we are talking about evidence of disease, based on biomarkers, to predict likelihood of developing dementia in the future. These differences emphasize the need for more specific research on risk communication in the context of AD and the implementation of a precision approach to dementia prevention.

In general, risk information is difficult to convey, and for at-risk individuals to understand [69, 70]. It may be even more difficult in dementia, as no fixed events define its onset [71]. As an additional barrier, the cognitive impairment that is inherent to a neurodegenerative disease such as AD could hamper communication and understanding. A particular challenge at this stage is that a lot of people in the general public use the terms AD and dementia interchangeably and are not aware of the new definition of AD as the underlying disease which may cause dementia at a later stage [41, 72]. There is a lot of (linguistic) confusion -also among professionals- making it harder to define optimal strategies to clearly communicate risk.

5.2 Future research directions

More evidence is warranted about the impact of dementia risk communication, to be able to adequately inform, make (shared) decisions, and manage expectations. More specifically, we need to systematically evaluate the relative merits of different approaches/strategies to risk communication on outcomes in three domains: (i) the cognitive one, reflecting peoples' risk perception and understanding, such as accuracy in answering questions related to probabilistic information; (ii) the affective domain, concerning the emotional impact and individuals' preferences for or satisfaction with the communication strategy, and; (iii) the behavioral domain, people's intentions to change behavior, or their actual actions, such as lifestyle modifications.

Furthermore, research should be directed at identifying those factors that influence individuals' reactions to getting to know their dementia risk, since individual differences between individuals may moderate the relation between risk communication and relevant outcomes. Hence, a one-size-fits-all approach does not work. This is especially relevant in light of educational background and cultural differences and minority groups. Insight into moderator

factors and individual differences could stimulate the adoption of a truly personalized, tailored approach to dementia prevention, taking into account the individual's characteristics and personal needs, preferences, values and situation.

Next, based on that evidence, we should develop an evidence-based dementia risk communication protocol. Although evidence about the impact of different strategies on relevant outcomes could form a strong base, input from health care professionals and the target population will be necessary to align perspectives and gain acceptance among stakeholders, which support the implementation of risk communication best-practices. Finally, (e-)tools are warranted to support professionals in communication and promote adherence to the communication protocol.

5.3. Conclusion

With the growing knowledge of AD and availability of biomarkers on the one hand, and the increasing focus on prevention strategies on the other hand, there is a growing demand to know one's risk of dementia in very early stages. This demand will even further increase with the upcoming of blood-based biomarkers and disease-modifying treatments. Communicating about the risk of developing dementia is thus crucial, yet challenging, because of the current lack of evidence on *what* to tell on an individual level (*i.e.*, the actual risk), and on *how* to optimally communicate about risk in a way that maximizes the desired impact of this information, and minimize its harms. Available evidence suggests that risk communication should be precise and include the use of absolute risks, visual displays and time frames, be based on a process of shared decision making and address the uncertainty inherent to any probability. Next steps required for the development of an evidence-based BHS protocol for dementia risk communication include the systematic evaluation of the relative merits of different strategies to risk communication on affective, cognitive and behavioral outcomes, with a special focus on individual differences.

LIST OF ABBREVIATIONS

AAN: American Academy of Neurology. AD: Alzheimer's disease. APOE: APOLIPOPROTEIN. A4: Anti-Amyloid Treatment in Asymptomatic Alzheimer's Disease Study. BBDPS: Barcelonaβeta Dementia Prevention Study. BHS: Brain Health Services. CES-D: Center for Epidemiologic Studies Depression Scale. MCI: mild cognitive impairment. PET: Positron Emission Tomography. SCD: subjective cognitive decline. SDM: shared decision-making. STAI: State-Trait Anxiety Inventory.

DECLARATIONS

Ethics approvals and consent to participate

The BBDPS was approved by the Independent Ethics Committee "Parc de Salut Mar", Barcelona, and registered at Clinicaltrials.gov (Identifier: NCT03847038). All participating subjects and signed the study's informed consent form that had also been approved by the Independent Ethics Committee "Parc de Salut Mar", Barcelona.

Consent for publication: Not applicable

Availability of data and materials

The data that support the findings of the BBDPS of are available from the corresponding author Carolina Minguillon, upon reasonable request.

Competing interests

GBF reports grants from Alzheimer Forum Suisse, Académie Suisse des Sciences Médicales, Avid Radiopharmaceuticals, Biogen, GE International, Guerbert, Association Suisse pour la Recherche sur l'Alzheimer, IXICO, Merz Pharma, Nestlé, Novartis, Piramal, Roche, Siemens, Teva Pharmaceutical Industries, Vifor Pharma, and Alzheimer's Association; he has received personal fees from AstraZeneca, Avid Radiopharmaceuticals, Elan Pharmaceuticals, GE International, Lundbeck, Pfizer, and TauRx Therapeutics.

PS has received consultancy fees (paid to the institution) from AC Immune, Alkermes, Alnylam, Anavex, Biogen, Brainstorm Cell, Cortexyme, Denali, EIP, ImmunoBrain Checkpoint, GemVax, Genentech, Green Valley, Novartis, Novo Noridisk, PeopleBio, Renew LLC, Roche. He is PI of studies with AC Immune, CogRx, FUJI-film/Toyama, IONIS, UCB, Vivoryon. He serves on the board of the Brain Research Center.

WF has received consultancy fees (paid to the institution) from Oxford Health Policy Forum CIC, Roche BV. She has been an invited speaker at Boehringer Ingelheim, Biogen MA Inc, and WebMD Neurology (Medscape). She has performed contract research for Biogen MA Inc and Boehringer Ingelheim. All funding is paid to her institution. WF is associate editor at Alzheimer's, Research & Therapy.

JLM is currently a full time employee of Lundbeck and has previously served as a consultant or at advisory boards for the following for-profit companies, or has given lectures in symposia sponsored by the following for-profit companies: Roche Diagnostics, Genentech, Novartis, Lundbeck, Oryzon, Biogen, Lilly, Janssen, Green Valley, MSD, Eisai, Alector, BioCross, GE Healthcare, ProMIS Neurosciences.

The other coauthors declare that they have no competing interests.

Funding

This paper was the product of a workshop funded by the Swiss National Science Foundation entitled "Dementia Prevention Services" (grant number: IZSEZ0_193593).

Additional funding was obtained from EURO-FINGERS, an EU Joint Programme - Neurodegenerative Disease Research (JPND) project. The EURO-FINGERS project is supported through the following funding organisations under the aegis of JPND - www.jpnd.eu: Finland, Academy of Finland; Germany, Federal Ministry of Education and Research; Spain, National Institute of Health Carlos III; Luxembourg, National Research Fund; Hungary, National Research, Development and Innovation Office; The Netherlands, Netherlands Organisation for Health Research and Development (ZonMW-Memorabel #733051102); Sweden, Swedish Research Council.

LNCV is funded by an Alzheimer Nederland fellowship (WE.15-2019-05).

The Barcelonaβeta Dementia Prevention Study and its registry have received funding from the Barcelona City Council (20190454), the Health Department of the Catalan Government, the Catalan Agency for Health Quality and Evaluation, and Biogen.

Hersenonderzoek.nl is supported by ZonMw-Memorabel (#73305095003), Gieskes-Strijbis Foundation, Alzheimer Nederland and Hersenstichting.

GBF received funding by: the EU-EFPIA Innovative Medicines Initiatives 2 Joint Undertaking (IMI 2 JU) "European Prevention of Alzheimer's Dementia consortium" (EPAD, grant agreement number: 115736) and "Amyloid Imaging to Prevent Alzheimer's Disease" (AMYPAD, grant agreement number: 115952); the Swiss National Science Foundation: "Brain connectivity and metacognition in persons with subjective cognitive decline (COSCODE): correlation with clinical features and in vivo neuropathology" (grant number: 320030_182772).

Authors' contributions

Leonie N.C. Visser, Carolina Minguillon, Wiesje M. van der Flier, and José Luis Molinuevo conceptualized this Paper, drafted the manuscript for intellectual content, and approved the manuscript.

Gonzalo Sánchez-Benavides, Marc Abramowicz, Karine Fauria, Jean Georges, Philip Scheltens, Jetske van der Schaar, and Marissa Zwan drafted specific parts of the manuscript, revised the manuscript for intellectual content, and approved the manuscript.

Daniele Altomare, Giovanni B. Frisoni, and Federica Ribaldi conceived and organized the workshop whence the Papers of the BHS series in this issue of *Alzheimer's Research & Therapy* originated, conceived the related editorial initiative, revised this manuscript for intellectual content, harmonized the manuscript with the other Papers of the BHS series, and approved the manuscript.

Acknowledgements

European Task Force for Brain Health Services (in alphabetical order): Marc ABRAMOWICZ, Daniele ALTOMARE, Frederik BARKHOF, Marcelo BERTHIER, Melanie BIELER, Kaj BLENNOW, Carol BRAYNE, Andrea BRIOSCHI, Emmanuel CARRERA, Gael CHÉTELAT, Chantal CSAJKA, Jean-François DEMONET, Alessandra DODICH, Bruno

DUBOIS, Giovanni B. FRISONI, Valentina GARIBOTTO, Jean GEORGES, Samia HURST, Frank JESSEN, Miia KIVIPELTO, David LLEWELLYN, Laura McWHIRTER, Richard MILNE, Carolina MINGUILLÓN, Carlo MINIUSSI, José Luis MOLINUEVO, Peter M NILSSON, Janice RANSON, Federica RIBALDI, Craig RITCHIE, Philip SCHELTENS, Alina SOLOMON, Wiesje VAN DER FLIER, Cornelia VAN DUIJN, Bruno VELLAS, Leonie VISSER.

The authors would like to express their gratitude to BBDPS participants.

REFERENCES

1. Smith AK, White DB, Arnold RM. Uncertainty--the other side of prognosis. *N Engl J Med* 2013, 368:2448-2450.

2. Stiggelbout AM, Pieterse AH, De Haes JC. Shared decision making: Concepts, evidence, and practice. *Patient Educ Couns* 2015, 98:1172-1179.

3. Livingston G, Huntley J, Sommerlad A, Ames D, Ballard C, Banerjee S, Brayne C, Burns A, Cohen-Mansfield J, Cooper C, et al. Dementia prevention, intervention, and care: 2020 report of the Lancet Commission. *Lancet* 2020.

4. Frisoni GB, Molinuevo JL, Altomare D, Carrera E, Barkhof F, Berkhof J, Delrieu J, Dubois B, Kivipelto M, Nordberg A, et al. Precision prevention of Alzheimer's and other dementias: Anticipating future needs in the control of risk factors and implementation of disease-modifying therapies. *Alzheimers Dement* 2020, 16:1457-1468.

5. Jack CR, Jr., Bennett DA, Blennow K, Carrillo MC, Dunn B, Haeberlein SB, Holtzman DM, Jagust W, Jessen F, Karlawish J, et al. NIA-AA Research Framework: Toward a biological definition of Alzheimer's disease. *Alzheimers Dement* 2018, 14:535-562.

6. World Health Organization: General information on risk communication. [Retrieved from: [https://www.who.int/risk-communication/background/en/]].on 13 December 2020].

7. Rothman AJ, Kiviniemi MT. Treating people with information: an analysis and review of approaches to communicating health risk information. *J Natl Cancer Inst Monogr* 1999:44-51.

8. Kunneman M, Smets EMA, Bouwman FH, Schoonenboom NSM, Zwan MD, Pel-Littel R, van der Flier WM. Clinicians' views on conversations and shared decision making in diagnostic testing for Alzheimer's disease: The ABIDE project. *Alzheimers Dement (N Y)* 2017, 3:305-313.

9. Visser LNC, van Maurik IS, Bouwman FH, Staekenborg S, Vreeswijk R, Hempenius L, de Beer MH, Roks G, Boelaarts L, Kleijer M, et al. Clinicians' communication with patients receiving a MCI diagnosis: The ABIDE project. *PLoS One* 2020, 15:e0227282.

10. Bell NR, Dickinson JA, Grad R, Singh H, Kasperavicius D, Thombs BD. Understanding and communicating risk: Measures of outcome and the magnitude of benefits and harms. *Can Fam Physician* 2018, 64:181-185.

11. Zipkin DA, Umscheid CA, Keating NL, Allen E, Aung K, Beyth R, Kaatz S, Mann DM, Sussman JB, Korenstein D, et al. Evidence-based risk communication: a systematic review. *Ann Intern Med* 2014, 161:270-280.

12. Gigerenzer G, Gaissmaier W, Kurz-Milcke E, Schwartz LM, Woloshin S. Helping Doctors and Patients Make Sense of Health Statistics. *Psychol Sci Public Interest* 2007, 8:53-96.

13. Peters E, Hibbard J, Slovic P, Dieckmann N. Numeracy skill and the communication, comprehension, and use of risk-benefit information. *Health Aff (Millwood)* 2007, 26:741-748.

14. Peters KR, Lynn Beattie B, Feldman HH, Illes J. A conceptual framework and ethics analysis for prevention trials of Alzheimer Disease. *Prog Neurobiol* 2013, 110:114-123.

15. Schermer MHN, Richard E. On the reconceptualization of Alzheimer's disease. *Bioethics* 2019, 33:138-145.

16. Kostoff RN. Actionable Causes of Alzheimer's disease. *Journal of Scientometic Research* 2017, 6:51-53.

17. Imtiaz B, Tolppanen AM, Kivipelto M, Soininen H. Future directions in Alzheimer's disease from risk factors to prevention. *Biochem Pharmacol* 2014, 88:661-670.

18. van der Flier WM, Scheltens P. Amsterdam Dementia Cohort: Performing Research to Optimize Care. *J Alzheimers Dis* 2018, 62:1091-1111.

19. McWhirter L, Ritchie C, Stone J, Carson A. Functional cognitive disorders: a systematic review. *Lancet Psychiatry* 2020, 7:191-207.

20. Fruijtier AD, Visser LNC, van Maurik IS, Zwan MD, Bouwman FH, van der Flier WM, Smets EMA. ABIDE Delphi study: topics to discuss in diagnostic consultations in memory clinics. *Alzheimers Res Ther* 2019, 11:77.

21. Visser LNC, Kunneman M, Murugesu L, van Maurik I, Zwan M, Bouwman FH, Schuur J, Wind HA, Blaauw MSJ, Kragt JJ, et al. Clinician-patient communication during the diagnostic workup: The ABIDE project. *Alzheimer's & Dementia: Diagnosis, Assessment & Disease Monitoring* 2019, 11:520-528.

22. Kunneman M, Pel-Littel R, Bouwman FH, Gillissen F, Schoonenboom NSM, Claus JJ, van der Flier WM, Smets EMA. Patients' and caregivers' views on conversations and shared decision making in diagnostic testing for Alzheimer's disease: The ABIDE project. *Alzheimers Dement (N Y)* 2017, 3:314-322.

23. Tochel C, Smith M, Baldwin H, Gustavsson A, Ly A, Bexelius C, Nelson M, Bintener C, Fantoni E, Garre-Olmo J, et al. What outcomes are important to patients with mild cognitive impairment or Alzheimer's disease, their caregivers, and health-care professionals? A systematic review. *Alzheimers Dement (Amst)* 2019, 11:231-247.

24. Jessen F, Amariglio RE, Buckley RF, van der Flier WM, Han Y, Molinuevo JL, Rabin L, Rentz DM, Rodriguez-Gomez O, Saykin AJ, et al. The characterisation of subjective cognitive decline. *Lancet Neurol* 2020, 19:271-278.

25. van Maurik IS, Slot RER, Verfaillie SCJ, Zwan MD, Bouwman FH, Prins ND, Teunissen CE, Scheltens P, Barkhof F, Wattjes MP, et al. Personalized risk for clinical progression in cognitively normal subjects-the ABIDE project. *Alzheimers Res Ther* 2019, 11:33.

26. Smedinga M, Tromp K, Schermer MHN, Richard E. Ethical Arguments Concerning the Use of Alzheimer's Disease Biomarkers in Individuals with No or Mild Cognitive Impairment: A Systematic Review and Framework for Discussion. *J Alzheimers Dis* 2018, 66:1309-1322.

27. Arias JJ, Cummings J, Grant AR, Ford PJ. Stakeholders' Perspectives on Preclinical Testing for Alzheimer's Disease. *J Clin Ethics* 2015, 26:297-305.

28. Bemelmans SA, Tromp K, Bunnik EM, Milne RJ, Badger S, Brayne C, Schermer MH, Richard E. Psychological, behavioral and social effects of disclosing Alzheimer's disease biomarkers to research participants: a systematic review. *Alzheimers Res Ther* 2016, 8:46.

29. Largent EA, Harkins K, van Dyck CH, Hachey S, Sankar P, Karlawish J. Cognitively unimpaired adults' reactions to disclosure of amyloid PET scan results. *PLoS One* 2020, 15:e0229137.

30. Christensen KD, Karlawish J, Roberts JS, Uhlmann WR, Harkins K, Wood EM, Obisesan TO, Le LQ, Cupples LA, Zoltick ES, et al. Disclosing genetic risk for Alzheimer's dementia to individuals with mild cognitive impairment. *Alzheimers Dement (N Y)* 2020, 6:e12002.

31. Ott BR, Pelosi MA, Tremont G, Snyder PJ. A Survey of Knowledge and Views Concerning Genetic and Amyloid PET Status Disclosure. *Alzheimers Dement (N Y)* 2016, 2:23-29.

32. Karlawish J. Addressing the ethical, policy, and social challenges of preclinical Alzheimer disease. *Neurology* 2011, 77:1487-1493.

33. Stites SD, Milne R, Karlawish J. Advances in Alzheimer's imaging are changing the experience of Alzheimer's disease. *Alzheimers Dement (Amst)* 2018, 10:285-300.

34. Bunnik EM, Richard E, Milne R, Schermer MHN. On the personal utility of Alzheimer's disease-related biomarker testing in the research context. *J Med Ethics* 2018, 44:830-834.

35. Seiffert DJ, McCarthy Veach P, LeRoy B, Guan W, Zierhut H. Beyond medical actionability: Public perceptions of important actions in response to hypothetical genetic testing results. *J Genet Couns* 2019, 28:355-366.

36. Vanderschaeghe G, Dierickx K, Vandenberghe R. Review of the Ethical Issues of a Biomarker-Based Diagnoses in the Early Stage of Alzheimer's Disease. *J Bioeth Inq* 2018, 15:219-230.

37. Lee AJ, Cunningham AP, Tischkowitz M, Simard J, Pharoah PD, Easton DF, Antoniou AC. Incorporating truncating variants in PALB2, CHEK2, and ATM into the BOADICEA breast cancer risk model. *Genet Med* 2016, 18:1190-1198.

38. Hartmann LC, Lindor NM. The Role of Risk-Reducing Surgery in Hereditary Breast and Ovarian Cancer. *N Engl J Med* 2016, 374:454-468.

39. AlzheimerEurope. The Value of Knowing. Findings of Alzheimer Europe's five country survey on public perceptions of Alzheimer's disease and views on the value of diagnosis. Luxembourg: Alzheimer Europe; 2011.

40. Robinson SM, Canavan M, O'Keeffe ST. Preferences of older people for early diagnosis and disclosure of Alzheimer's disease (AD) before and after considering potential risks and benefits. *Arch Gerontol Geriatr* 2014, 59:607-612.

41. Smedinga M, Bunnik EM, Richard E, Schermer MHN. The Framing of "Alzheimer's Disease": Differences Between Scientific and Lay Literature and Their Ethical Implications. *Gerontologist* 2020.

42. Petersen RC. Mild cognitive impairment as a diagnostic entity. *J Intern Med* 2004, 256:183-194.

43. Petersen RC, Lopez O, Armstrong MJ, Getchius TSD, Ganguli M, Gloss D, Gronseth GS, Marson D, Pringsheim T, Day GS, et al. Practice guideline update summary: Mild cognitive impairment: Report of the Guideline Development, Dissemination, and Implementation Subcommittee of the American Academy of Neurology. *Neurology* 2018, 90:126-135.

44. van Maurik IS, Vos SJ, Bos I, Bouwman FH, Teunissen CE, Scheltens P, Barkhof F, Frolich L, Kornhuber J, Wiltfang J, et al. Biomarker-based prognosis for people with mild cognitive impairment (ABIDE): a modelling study. *Lancet Neurol* 2019, 18:1034-1044.

45. Ebenau JL, Timmers T, Wesselman LMP, Verberk IMW, Verfaillie SCJ, Slot RER, van Harten AC, Teunissen CE, Barkhof F, van den Bosch KA, et al. ATN classification and clinical progression in subjective cognitive decline: The SCIENCe project. *Neurology* 2020, 95:e46-e58.

46. Lopez Lopez C, Tariot PN, Caputo A, Langbaum JB, Liu F, Riviere ME, Langlois C, Rouzade-Dominguez ML, Zalesak M, Hendrix S, et al. The Alzheimer's Prevention Initiative Generation Program: Study design of two randomized controlled trials for individuals at risk for clinical onset of Alzheimer's disease. *Alzheimers Dement (N Y)* 2019, 5:216-227.

47. Sperling RA, Rentz DM, Johnson KA, Karlawish J, Donohue M, Salmon DP, Aisen P. The A4 study: stopping AD before symptoms begin? *Sci Transl Med* 2014, 6:228fs213.

48. Frisoni GB, Barkhof F, Altomare D, Berkhof J, Boccardi M, Canzoneri E, Collij L, Drzezga A, Farrar G, Garibotto V, et al. AMYPAD Diagnostic and Patient Management Study: Rationale and design. *Alzheimers Dement* 2019, 15:388-399.

49. Radloff LS. The CES-D Scale: a self-report depression scale for research in the general population. . *Appl Psych Meas* 1977:385-401.

50. Spielberger CD, Gorsuch RL, Lushene R, Vagg PR, Jacobs GA. *Manual for the State-Trait Anxiety Inventory.* . Palo Alto, CA: Consulting Psychologists Press; 1983.

51. Burns JM, Johnson DK, Liebmann EP, Bothwell RJ, Morris JK, Vidoni ED. Safety of disclosing amyloid status in cognitively normal older adults. *Alzheimers Dement* 2017, 13:1024-1030.

52. Green RC, Roberts JS, Cupples LA, Relkin NR, Whitehouse PJ, Brown T, Eckert SL, Butson M, Sadovnick AD, Quaid KA, et al. Disclosure of APOE genotype for risk of Alzheimer's disease. *N Engl J Med* 2009, 361:245-254.

53. Zwan Mea. Dutch Brain Research Registry for study participant recruitment: design and first results. *Alzheimer's & Dementia: Translational Research & Clinical Interventions* [accepted for publication].

54. Fagerlin A, Zikmund-Fisher BJ, Ubel PA. Helping patients decide: ten steps to better risk communication. *J Natl Cancer Inst* 2011, 103:1436-1443.

55. van de Water LF, van Kleef JJ, Dijksterhuis WPM, Henselmans I, van den Boorn HG, Vaarzon Morel NM, Schut KF, Daams JG, Smets EMA, van Laarhoven HWM. Communicating treatment risks and benefits to cancer patients: a systematic review of communication methods. *Qual Life Res* 2020, 29:1747-1766.

56. van Maurik IS, Visser LN, Pel-Littel RE, van Buchem MM, Zwan MD, Kunneman M, Pelkmans W, Bouwman FH, Minkman M, Schoonenboom N, et al. Development and Usability of ADappt: Web-Based Tool to Support Clinicians, Patients, and Caregivers in the Diagnosis of Mild Cognitive Impairment and Alzheimer Disease. *JMIR Form Res* 2019, 3:e13417.

57. Koenig BA, Silverberg HL. Understanding probabilistic risk in predisposition genetic testing for Alzheimer disease. *Genet Test* 1999, 3:55-63.

58. Visser LNC, Pelt SAR, Kunneman M, Bouwman FH, Claus JJ, Kalisvaart KJ, Hempenius L, de Beer MH, Roks G, Boelaarts L, et al. Communicating uncertainties when disclosing diagnostic test results for (Alzheimer's) dementia in the memory clinic: The ABIDE project. *Health Expect* 2019, 23:52-62.

59. Campbell S, Manthorpe J, Samsi K, Abley C, Robinson L, Watts S, Bond J, Keady J. Living with uncertainty: Mapping the transition from pre-diagnosis to a diagnosis of dementia. *J Aging Stud* 2016, 37:40-47.

60. van der Flier WM, Kunneman M, Bouwman FH, Petersen RC, Smets EMA. Diagnostic dilemmas in Alzheimer's disease: Room for shared decision making. *Alzheimers Dement (N Y)* 2017, 3:301-304.

61. Grad R, Legare F, Bell NR, Dickinson JA, Singh H, Moore AE, Kasperavicius D, Kretschmer KL. Shared decision making in preventive health care: What it is; what it is not. *Can Fam Physician* 2017, 63:682-684.

62. Berger ZD, Brito JP, Ospina NS, Kannan S, Hinson JS, Hess EP, Haskell H, Montori VM, Newman-Toker DE. Patient centred diagnosis: sharing diagnostic decisions with patients in clinical practice. *BMJ* 2017, 359:j4218.

63. Stiggelbout AM, van der Weijden T, De Wit MP, Frosch D, Legare F, Montori VM, Trevena L, Elwyn G. Shared decision making: really putting patients at the centre of healthcare. *BMJ* 2012, 344:e256.

64. Visser LNC, Kunneman M, Murugesu L, van Maurik I, Zwan M, Bouwman FH, Schuur J, Wind HA, Blaauw MSJ, Kragt JJ, et al. Clinician-patient communication during the diagnostic workup: The ABIDE project. *Alzheimers Dement (Amst)* 2019, 11:520-528.

65. Grill JD, Raman R, Ernstrom K, Sultzer DL, Burns JM, Donohue MC, Johnson KA, Aisen PS, Sperling RA, Karlawish J, Team AS. Short-term Psychological Outcomes of Disclosing Amyloid Imaging Results to Research Participants Who Do Not Have Cognitive Impairment. *JAMA Neurol* 2020.

66. de Wilde A, van Buchem MM, Otten RHJ, Bouwman F, Stephens A, Barkhof F, Scheltens P, van der Flier WM. Disclosure of amyloid positron emission tomography results to individuals without dementia: a systematic review. *Alzheimers Res Ther* 2018, 10:72.

67. Millard FB, Kennedy RL, Baune BT. Dementia: opportunities for risk reduction and early detection in general practice. *Aust J Prim Health* 2011, 17:89-94.

68. Robinson L, Dickinson C, Magklara E, Newton L, Prato L, Bamford C. Proactive approaches to identifying dementia and dementia risk; a qualitative study of public attitudes and preferences. *BMJ Open* 2018, 8:e018677.

69. Bogardus ST, Jr., Holmboe E, Jekel JF. Perils, pitfalls, and possibilities in talking about medical risk. *JAMA* 1999, 281:1037-1041.

70. Lipkus IM. Numeric, verbal, and visual formats of conveying health risks: suggested best practices and future recommendations. *Med Decis Making* 2007, 27:696-713.

71. Petersen RC, Doody R, Kurz A, Mohs RC, Morris JC, Rabins PV, Ritchie K, Rossor M, Thal L, Winblad B. Current concepts in mild cognitive impairment. *Arch Neurol* 2001, 58:1985-1992.

72. Frisoni GB, Ritchie C, Carrera E, Nilsson P, Ousset PJ, Molinuevo JL, Dubois B, Scheltens P, Minoshima S. Re-aligning scientific and lay narratives of Alzheimer's disease. *Lancet Neurol* 2019, 18:918-919.

73. Harkins K, Sankar P, Sperling R, Grill JD, Green RC, Johnson KA, Healy M, Karlawish J. Development of a process to disclose amyloid imaging results to cognitively normal older adult research participants. *Alzheimers Res Ther* 2015, 7:26.

74. Langlois CM, Bradbury A, Wood EM, Roberts JS, Kim SYH, Riviere ME, Liu F, Reiman EM, Tariot PN, Karlawish J, Langbaum JB. Alzheimer's Prevention Initiative Generation Program: Development of an APOE genetic counseling and disclosure process in the context of clinical trials. *Alzheimers Dement (N Y)* 2019, 5:705-716.

75. Jessen F, Amariglio RE, van Boxtel M, Breteler M, Ceccaldi M, Chetelat G, Dubois B, Dufouil C, Ellis KA, van der Flier WM, et al. A conceptual framework for research on subjective cognitive decline in preclinical Alzheimer's disease. *Alzheimers Dement* 2014, 10:844-852.

76. Molinuevo JL, Rabin LA, Amariglio R, Buckley R, Dubois B, Ellis KA, Ewers M, Hampel H, Kloppel S, Rami L, et al. Implementation of subjective cognitive decline criteria in research studies. *Alzheimers Dement* 2017, 13:296-311.

77. Li J, Ogrodnik M, Devine S, Auerbach S, Wolf PA, Au R. Practical risk score for 5-, 10-, and 20-year prediction of dementia in elderly persons: Framingham Heart Study. *Alzheimers Dement* 2018, 14:35-42.

78. Chao S, Roberts JS, Marteau TM, Silliman R, Cupples LA, Green RC. Health behavior changes after genetic risk assessment for Alzheimer disease: The REVEAL Study. *Alzheimer Dis Assoc Disord* 2008, 22:94-97.

Figure 1. Impact of dementia risk disclosure on 69 low-risk versus 20 high-risk individuals with SCD from the Barcelonaβeta Dementia Prevention Study.

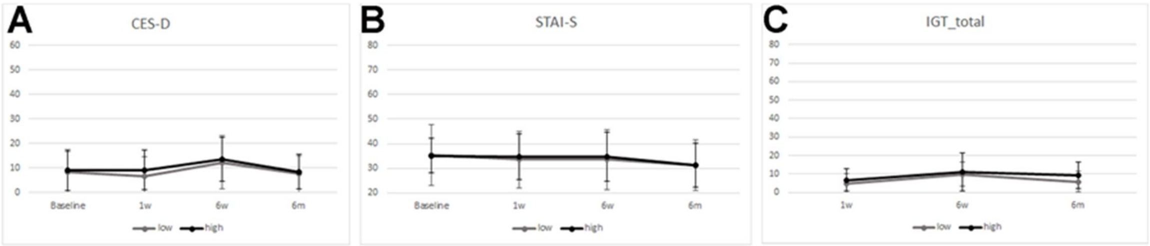

(*Figure 1 is also added as a separate file*)

Data at baseline, 1 week, 6 weeks and 6 months post-disclosure on A: depressive symptoms (CES-D) and B: anxiety levels (STAI-State). Graph C displays test-related distress (IGT-AD scale) at 1-week, 6-week and 6-month post-disclosure. Means and standard deviations are shown. Y-axis shows full scale range.

Table 1. An overview of studies investigating the impact of disclosure of APOE and Amyloid PET test results to research participants.

Publication	Project/study name	Type of study	Disclosure of	Main finding(s)
Green et al 2009 [52]	The Risk Evaluation and Education for Alzheimer's Disease (REVEAL) Study	RCT	*APOE*	No differences between the two groups (disclosure *vs* no disclosure) in changes in time-averaged measures of anxiety, depression or test-related distress (measured at 6 weeks, 6 months and 1 year).<br><br>The *ε4*-negative subgroup had a significantly lower level of test-related distress than did the *ε4*-positive one.
Chao et al 2008 [78]	REVEAL	RCT	*APOE*	Participants who learned they were ε4 positive were significantly more likely than ε4 negative participants to report AD-specific health behavioral change 1 year after disclosure.
Bemelmans et al 2016 [28]	N/A	Systematic review	*APOE*	In cognitively unimpaired research participants with a first-degree relative with AD, disclosure of *APOE-ε4* positivity does not lead to elevated anxiety and depression levels.<br><br>It does increase test-related distress.<br><br>It results in behavioral changes concerning insurance and health.
Langlois et al 2019 [74]	Alzheimer's Prevention Initiative Generation Program	RCT	*APOE*	Standard protocol for disclosure is reported.

				Analyses have not been published yet.
Harkins et al 2015 [73]	Anti-Amyloid Treatment in Asymptomatic Alzheimer's Disease (A4) Study.	Modified Delphi study to develop consensus on best practices	Amyloid PET	Standard protocol for disclosure is reported.
Burns et al 2017 [51]	University of Kansas Alzheimer's Prevention through Exercise [APEX]	RCT	Amyloid PET	Depressive symptoms were stable throughout the visits and not different between groups (elevated vs non-elevated amyloid).<br><br>Anxiety symptoms peaked at a low level on the day of disclosure in the "elevated" group but were not sustained at 6 weeks or 6 months.<br><br>Individuals with elevated amyloid had slightly higher total levels of test-related distress compared with the non-elevated amyloid group at 6 weeks and 6 months post-disclosure.
Largent et al 2020 [29]	Study of Knowledge and Reactions to Amyloid Testing (SOKRATES) recruiting participants from the A4 and Longitudinal Evaluation of Amyloid Risk and Neurodegeneration (LEARN) trials	Observational study	Amyloid PET	Participants generally understood that an "elevated" amyloid PET scan result means increased but presently unquantifiable risk of developing AD dementia.<br><br>Participants who received an "elevated" result often wanted more information regarding the result.<br><br>An "elevated" result sparked negative emotions that decreased but did not entirely dissipate with time, but did not lead to extreme distress.

				Support the safety of disclosing amyloid imaging results to cognitively unimpaired persons following pre-test assessments of knowledge and psychological well-being.<br><br>Participants who received an “elevated” result reported contemplating and making changes to health behaviors and future plans to a greater extent.<br><br>Participants with elevated brain amyloid viewed the amyloid PET scan result as a serious, sensitive piece of health information.<br><br>Irrespective of their brain amyloid status, participants were mindful that their amyloid PET scan result had implications for themselves and also for others.
Grill et al 2020 [65]	A4 study and LEARN trials	Observational study	Amyloid PET	Participants in the elevated amyloid group, compared with participants who learned that they had a not elevated amyloid result, were not more likely to experience short-term increases in depression, anxiety, or suicidality
Wilde et al 2018 [66]	N/A	Systematic review	Amyloid PET	The sparse data available suggest that disclosure of amyloid PET results has a low risk of psychological harm in the context of clinical trials, whereas both participants and professionals seem to support disclosure.<br><br>More research is needed about the psychological impact of PET disclosure, and the predictive value of results at an individual level.<br><br>Communication materials and strategies to support disclosure of amyloid PET results should be further developed and prospectively evaluated.

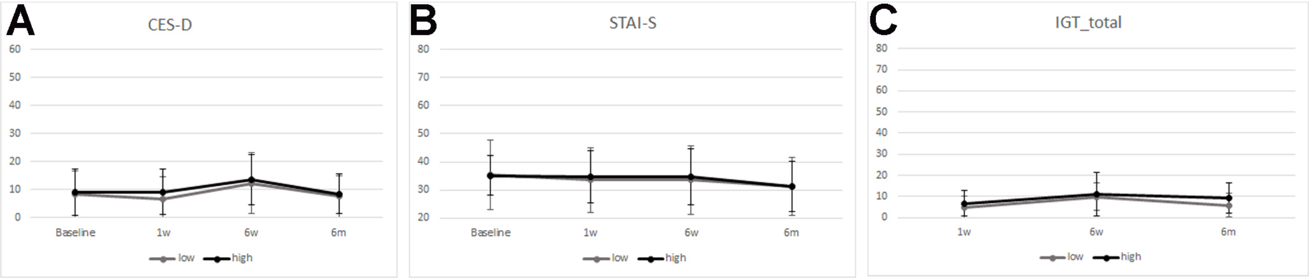
A
CES-D
60
50
40
30
20
10
0
Baseline
1w
6w
6m
low
high
B
STAI-S
80
70
60
50
40
30
20
Baseline
1w
6w
6m
low
high
C
IGT_total
80
70
60
50
40
30
20
10
0
1w
6w
6m
low
high

Dementia risk communication.

A user manual for Brain Health Services – Part 3 of 6

Leonie N.C. Visser*, PhD[1,2]; Carolina Minguillon*, PhD[3,4,5]; Gonzalo Sánchez-Benavides, PhD[3,4,5]; Marc Abramowicz, MD, PhD[6]; Daniele Altomare, PhD[7,8]; Karine Fauria, PhD[3,4]; Giovanni B Frisoni, MD[7,8]; Jean Georges, BA[9]; Federica Ribaldi, MS[6,7,10,11]; Philip Scheltens, MD, PhD[1]; Jetske van der Schaar, MA[1]; Marissa Zwan, PhD[1]; Wiesje M. van der Flier[+], PhD[1,12]; José Luis Molinuevo[+], MD, PhD[3] *on behalf of the European Task Force for Brain Health Services*

*These authors contributed equally to this work (shared first author).

[+]These authors contributed equally to this work (shared last author).

[1]Alzheimer Center Amsterdam, Department of Neurology, Amsterdam Neuroscience, Vrije Universiteit Amsterdam, Amsterdam UMC, Amsterdam, The Netherlands.

[2]Center for Alzheimer Research, Division of Clinical Geriatrics, Department of Neurobiology, Care Sciences and Society, Karolinska Institutet, Solna, Sweden.

[3]Barcelonaβeta Brain Research Center (BBRC), Pasqual Maragall Foundation, Barcelona, Spain.

[4]IMIM (Hospital del Mar Medical Research Institute), Barcelona, Spain.

[5]Centro de Investigación Biomédica en Red de Fragilidad y Envejecimiento Saludable (CIBERFES), Madrid, Spain.

[6]Division of Genetic Medicine, Department of Diagnostics, Geneva University Hospitals and University of Geneva, Switzerland.

[7]Laboratory of Neuroimaging of Aging (LANVIE), University of Geneva, Geneva, Switzerland.

[8]Memory Clinic, Geneva University Hospitals, Geneva, Switzerland.

[9]Alzheimer Europe, Luxembourg.

[10]Laboratory of Alzheimer's Neuroimaging and Epidemiology (LANE), Saint John of God Clinical Research Centre, Brescia, Italy.

[11]Department of Molecular and Translational Medicine, University of Brescia, Brescia, Italy.

[12]Department of Epidemiology and Data Science, Vrije Universiteit Amsterdam, Amsterdam UMC, Amsterdam, The Netherlands.

SUPPLEMENTARY MATERIAL

Supplement A. Summary of recommendations on how to disclose test results for dementia risk assessment in context of clinical trials.

Supplement B. The Barcelonaβeta Dementia Prevention Study and its associated registry.

Figure B1. Schematic representation of the Barcelonaβeta Dementia Prevention Study.

Table B1. Sample characteristics of the Barcelonaβeta Dementia Prevention Study participants at baseline visit.

Supplement A – Summary of recommendations on how to disclose test results for dementia risk assessment in context of clinical trials

We synthesized the following recommendations for disclosure of APOE- and amyloid PET status in the context of clinical trials (mainly extracted from [51, 73, 74]):

1. Before screening, delivering educational brochures for potential participants where the main points of the study, meaning of the test and understanding of the test results are covered in plain, understandable language.
2. At screening, assessing the emotional status (to assess eligibility to receiving the result) and the understanding of the study/test/consequences of the result using the same language as in the study's brochure.
3. In the case of PET, the scan should be performed in a separate day from the educational/screening session.
4. At disclosure, performed on a separate day from blood draw/scanning, review the educational contents and their understanding, assess emotional status before proceeding with disclosure. For PET results disclosure, positivity is explained as having a higher risk, without specification of the actual risk.
5. Establish a minimum number of follow-up visits (telephonic, in- person) to assess the possible emotional impact of disclosure and have additional monitoring and follow-up for participants showing distress.

Supplement B – The Barcelonaβeta Dementia Prevention Study and its associated registry

The Barcelonaβeta Brain Research Center (BBRC) implemented the Barcelonaβeta Dementia Prevention Study (BBDPS) in 2018 including an associated registry aiming at recruiting SCD and MCI individuals.

BBDPS was presented at a press conference (May 16th, 2018). The study and its registry were launched and general population were invited to register in the study web page if they "were feeling changes in their memory or cognitive status". The web-based selection system is designed as to allow the establishment of a registry containing the a priori eligible persons (*a priori* IN) interested in participating in the study, using an algorithm designed to include participants that meet certain criteria (i.e. age (60-80 y.o); >3 SCDplus features [75, 76]; availability). Those not fulfilling the criteria are annotated as *a priori* OUT. To examine the efficiency of the registry in getting the target population, we implemented a simple validation analysis: 232 persons were randomly chosen from the registry, (168 *a priori* IN and 64 *a priori* OUT). A neuropsychologist called each of them to evaluate their cognitive status, applying the same SCDplus questionnaire that was designed for the web (among other questions). Using the same questionnaire, but being able to further examine each of the participants' answers by a professional (since the individual is able to verbally justify their answers), enabled to take into account a clinical opinion for the outcome of the SCDplus questionnaire. Thus, an *a priori* eligible status could change after the phone examinations to OUT and, conversely, an *a priori* OUT status could change to IN after the telephonic interview. Out of 168 *a priori* IN, 137 (80%) continued as IN. Nevertheless, a dozen of those still considered as IN have been excluded from the *a priori* IN registry after further examination. This is mainly because the further analysis involved certain clinical criteria (e.g. illnesses non-compatible with the study) and MRI eligibility. Out of 64 *a priori* OUT, 55 (85%) continued as OUT, while the remaining nine were re-considered as IN, after the phone call and professional evaluation.

Figure B1. Schematic representation of the BBDPS.

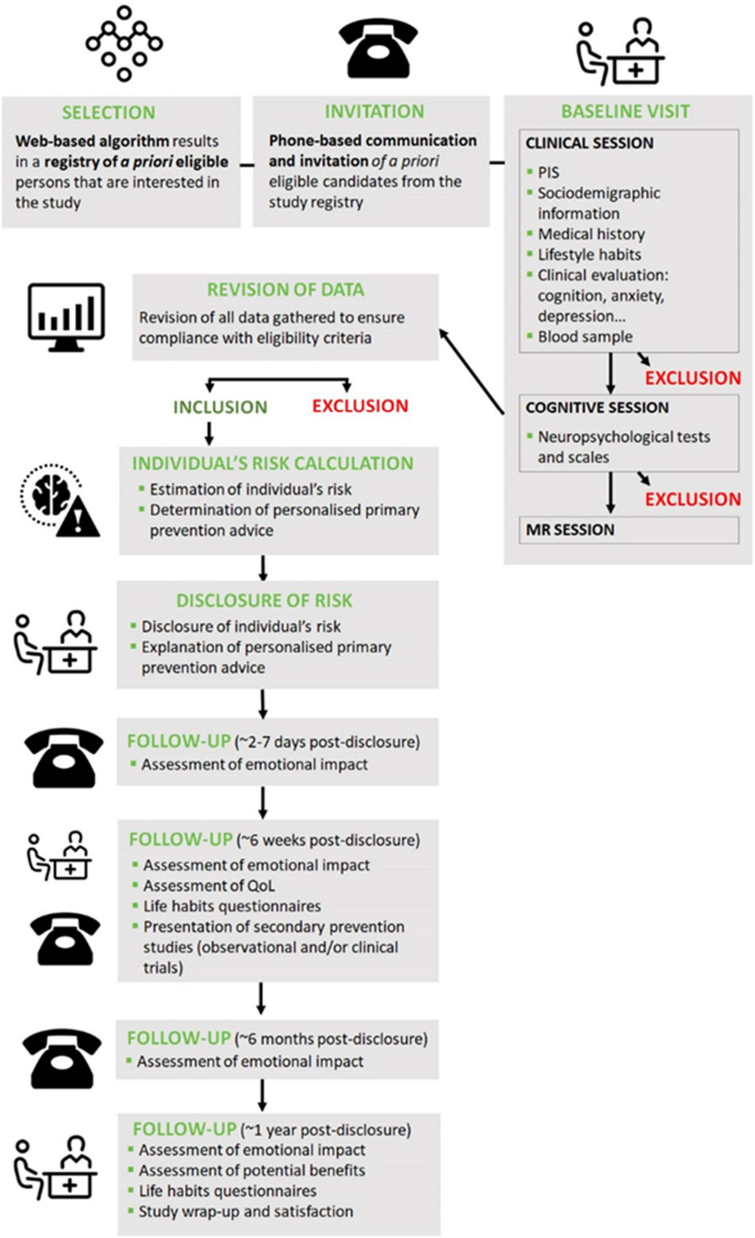

A priori IN individuals gathered from the study's registry, were contacted on the phone to schedule an in-person baseline visit and sent by email the study's brochure, in which the study, the main risk factors for dementia, how their specific risk would be calculated and how to interpret this risk estimates were explained in plain language. At baseline, the neurologist in charge of the visit explained the study: She/he would use the brochure to go through all this information and an *ad hoc* questionnaire was design to assess the participant's understanding. Once informed consent was granted, the variables (e.g. clinical, sociodemographic, lifestyle, cognitive, dementia blood workup) that feed the risk algorithm [77] as well as the personalized

prevention plans and guidelines obtained, were gathered. These initial data obtained also serve for evaluating compliance with inclusion criteria as well as the diagnostic classification of the subject in a subgroup of MCI or SCD. At baseline, the first emotional evaluation of the participant is also performed (and excluded if exceeded established cut-offs). This baseline visit included the following sessions: Clinical session, Cognitive session, Magnetic resonance (MR) session. All data gathered at baseline was reviewed by BBRC researchers to assess the final inclusion of a study participant. Individual risk estimates were calculated and personalized prevention plans selected depending on the participant's specific risk profile and their diagnostic classification. At an in-person disclosure visit, after assessing the emotional status of the participant, the individual's risk estimates were disclosed in function of their diagnostic classification followed by an explanation of personalized primary prevention advice. Individuals within the MCI diagnostic category, may be offered other research studies and trials they may benefit from, that are undergoing at BBRC or collaborating institutions. After disclosure, a telephonic follow-up was performed between 2 to 7 days to evaluate emotional impact. Further telephonic (or in-person) follow-up was performed ≈6 weeks post-disclosure to evaluate again any possible emotional impact. In addition, depending on their specific risk profile, secondary prevention strategies were discussed and subjects may be invited to participate in ongoing studies (observational and/or clinical trials). Please note that this follow-up visit may also be in-person. Approximately 6 months post-disclosure a telephonic follow-up was performed to evaluate emotional impact. Finally, in a last in-person visit, one year (+/-1 month) post-disclosure, the possible emotional impact was again assessed as well as the possible benefits derived from the instalment of the personalized prevention plan.

Table B1. Sample characteristics of the BBDPS participants at baseline visit.

	low risk	high risk	p-value
n	69	20	
Age, mean (std)	64.1 (2.6)	72.7 (3.2)	<0.001*
Education, mean (std)	15.3 (3.9)	12.9 (4.8)	0.065
sex, female, count (%)	38 (55.1%)	10 (50%)	0.689
% of risk, mean (std)	3.5% (0.4)	13.7% (5.8)	<0.001*
CES-D, mean (std)	8.5 (8.3)	9.1 (8.2)	0.796
STAI-T, mean (std)	35.5 (10.5)	35.0 (9.4)	0.949
STAI-S, mean (std)	35.3 (12.5)	35.2 (7.1)	0.837

Notes. CES-D, Center for Epidemiologic Studies Depression Scale; STAI, State-Trait Anxiety Inventory; STAI-S, STAI state; STAI-T, STAI trait; std, standard deviation.

Protocols for precision dementia risk reduction.

A user manual for Brain Health Services – Part 4 of 6

Alina Solomon*, MD[1,2,3]; Ruth Stephen*, PhD[1]; Daniele Altomare, PhD[6,7]; Emmanuel Carrera, MD; Giovanni B Frisoni, MD[6,7]; Jenni Kulmala PhD[4]; José Luis Molinuevo, MD[8]; Peter Nilsson, MD[9]; Tiia Ngandu, MD[2,4]; Federica Ribaldi, MS[6,7,10,11]; Bruno Vellas, MD[12]; Philip Scheltens, MD[13]; Miia Kivipelto, MD[1,2,3,4,5] *on behalf of the European Task Force for Brain Health Services*

*These authors contributed equally to this work (shared first author).

[1]Neurology, Institute of Clinical Medicine, University of Eastern Finland, Kuopio, Finland.

[2]Division of Clinical Geriatrics, NVS, Karolinska Institutet, Stockholm, Sweden.

[3]Ageing Epidemiology Research Unit, School of Public Health, Imperial College London, UK.

[4]Department of Public Health Solutions, Public Health Promotion Unit, Finnish Institute for Health and Welfare, Helsinki, Finland.

[5]Institute of Public Health and Clinical Nutrition, University of Eastern Finland, Kuopio, Finland.

[6]Laboratory of Neuroimaging of Aging (LANVIE), University of Geneva, Geneva, Switzerland.

[7]Memory Clinic, Geneva University Hospitals, Geneva, Switzerland.

[8]Barcelonaβeta Brain Research Center (BBRC), Pasqual Maragall Foundation, Barcelona, Spain.

[9]Department of Clinical Sciences, Lund University, Skåne University Hospital, Malmö, Sweden.

[10]Laboratory of Alzheimer's Neuroimaging and Epidemiology (LANE), Saint John of God Clinical Research Centre, Brescia, Italy.

[11]Department of Molecular and Translational Medicine, University of Brescia, Brescia, Italy.

[12]Gérontopole of Toulouse, University Hospital of Toulouse (CHU-Toulouse), Toulouse, France.

[13]Alzheimer Center Amsterdam, Department of Neurology, Amsterdam Neuroscience, Vrije Universiteit Amsterdam, Amsterdam UMC, Amsterdam, The Netherlands.

Corresponding author

Alina Solomon

University of Eastern Finland, Kuopio, Finland

Email: alina.solomon@uef.fi. Tel.: 14686905822; Fax: 14686905954

MANUSCRIPT DETAILS

Character count title (with spaces): 101.

Word count abstract: 332.

Word count manuscript: 4630.

Number of references: 72.

Number of boxes: 1.

Number of tables: 4.

Number of figures: 1.

ABSTRACT

Although prevention of dementia and late-life cognitive decline is a major public health priority, there are currently no generally established prevention strategies or operational models for implementing such strategies into practice.

This article reviews available evidence from multidomain/complex dementia prevention trials targeting several risk factors and disease mechanisms simultaneously, in individuals without dementia at baseline. Based on the findings, we formulate recommendations for implementing precision risk reduction strategies into new services called Brain Health Services.

A literature search was conducted using medical databases (MEDLINE via PubMed and SCOPUS) to select relevant studies: non-pharmacological multidomain interventions (i.e. combining two or more intervention domains); target population including individuals without dementia; and primary outcomes including cognitive/functional performance changes, and/or incident cognitive impairment or dementia. Further literature searches covered the following topics: sub-group analyses assessing potential modifiers for the intervention effect on cognition in the multidomain prevention trials; dementia risk scores used as surrogate outcomes in multidomain prevention trials; dementia risk scores in relation to brain pathology markers; and cardiovascular risk scores in relation to dementia.

Multidomain intervention studies conducted so far appear to have mixed results and substantial variability in target populations, format and intensity of interventions, choice of control conditions, and outcome measures. Most trials were conducted in high-income countries. Differences in design between the larger, longer-term trials that met vs did not meet their primary outcomes suggest that multidomain intervention effectiveness may be dependent on a precision prevention approach, i.e., successfully identifying the at-risk groups who are most likely to benefit. One such successful trial has already developed an operational model for implementing the intervention into practice.

Evidence on the efficacy of risk reduction interventions is promising, but not yet conclusive. More long-term multidomain randomized controlled trials are needed to fill the current evidence gaps, especially concerning low- and middle-income countries and integration of dementia prevention with existing cerebrovascular prevention programs. A precision risk reduction approach may be most effective for dementia prevention. Such an approach could be implemented in Brain Health Services.

Keywords

Brain Health Services; dementia; aging; Alzheimer's disease; prevention; dementia risk; risk reduction

1. BACKGROUND

Although prevention of dementia and late-life cognitive decline is a major public health priority, there are currently no generally established prevention strategies or operational models for implementing such strategies into practice [1]. During the past 20 years, epidemiological studies have pointed out several modifiable risk factors for dementia, including cardiovascular, metabolic and lifestyle-related factors (e.g. hypertension, hyperlipidemia, diabetes, obesity, physical inactivity, unhealthy dietary habits, smoking, excessive alcohol consumption, social isolation) [2]. In 2019, the World Health Organization (WHO) published the first guidelines for risk reduction of cognitive decline and dementia [3]. The guidelines were developed to provide evidence-based recommendations on interventions aiming to delay or prevent the onset of cognitive decline and dementia. The reviewed evidence covered interventions including physical activity, tobacco cessation, nutrition, cognitive training, social activity, interventions for alcohol use disorders, and management of weight, hypertension, diabetes, dyslipidemia, depression, and hearing loss [3].

According to the WHO, these risk reduction guidelines are targeted primarily at health care providers working at a first or second level facility or at district level, including basic outpatient and inpatient services. While the WHO has pointed out several key considerations for implementation, it is not yet fully clear exactly how the recommendations should be tailored to specific populations, as well as different healthcare system contexts. Due to the complex multifactorial etiology of dementia, and variations in risk factors between different individuals and populations, a “one size fits all” approach to prevention is not going to work. The current risk reduction guidelines are also based on interventions targeting single risk factors. However, overall dementia risk is most often the result of a combination of risk and protective factors that may have different contributions in different individuals or at different life stages. Thus, a precision risk reduction approach is most likely to be effective, i.e., tailoring the right interventions for the right people and at the right time. Operational models for risk reduction interventions would also have to take into account the local or national specifics of both public health policies and healthcare systems.

Many multifactorial dementia risk scores have already been developed for early identification of at-risk individuals who may also benefit most from preventive interventions [4]. Although such risk scores could in principle facilitate precision risk reduction by e.g. highlighting an individual’s specific combination of risk factors and facilitating more tailored interventions, the majority of such risk scores are not yet sufficiently validated, and/or have not been tested in actual prevention trials. In addition, dementia shares many risk factors with other chronic diseases such as cardiovascular conditions (CVD), diabetes, or stroke. Validated risk scores for such conditions are already used as

part of established prevention programs [5]. However, it is not clear to what extent vascular/diabetes risk scores could be useful in the context of dementia prevention and facilitate the integration of dementia prevention within other established prevention programs.

This article reviews available evidence from complex/multidomain dementia prevention trials targeting several risk factors and disease mechanisms simultaneously, in individuals without dementia at baseline. A key aspect of the evidence review concerns the use of dementia and CVD risk scores in such prevention trials. Based on the findings, we formulate some practical recommendations for implementing precision risk reduction strategies (see the Box) into new services called Brain Health Services (BHSs). Currently, dementia prevention falls under the domain of memory clinics. However, the current memory clinics have been designed for the needs of patients with overt cognitive and/or behavioral disorders and are ill-equipped to deal with a population of cognitive unimpaired individuals and their growing demand for dementia prevention and cognitive enhancement interventions (Altomare et al., *this issue*). We envision the development of new BHSs, with specific missions including dementia risk profiling (Ranson et al., *this issue*), dementia risk communication (Visser et al., *this issue*), dementia risk reduction (*the present paper*), and cognitive enhancement (Brioschi et al., *this issue*); and with specific societal challenges (Milne et al., *this issue*). This will be the fourth part of a Special Issue series of six articles, published in *Alzheimer's Research & Therapy*, which together provide a user manual for BHSs.

2. MULTIDOMAIN INTERVENTIONS

2.1. Effects of multidomain interventions on cognition and related outcomes

A literature search was conducted using medical databases (MEDLINE via PubMed and SCOPUS), and keywords such as “multidomain”, “intervention”, “dementia”, “cognition”, “cognitive decline” “risk reduction”. The following criteria were used to select relevant studies: non-pharmacological multidomain interventions (defined as combining two or more intervention domains); target population including individuals without dementia at baseline; and primary outcomes including cognitive/functional performance, and/or incident mild cognitive impairment (MCI) or dementia. The 14 identified studies are summarized in Table 1.

Most of the trials were conducted in high-income countries. There was substantial variability in the target populations, format and intensity of the interventions, choice of control conditions, and outcome measures. Recruited participants were aged between 40-80 years and varied from relatively unselected primary care populations to general populations with risk factors for dementia, and patients with MCI. The sample size ranged from 56 to 3526 participants, and duration of the intervention from 8 weeks to 10 years (1 year or longer in 9 out of 14 trials). The interventions included intensive lifestyle programs offering various combinations of diet advice, dietary supplements, physical exercise advice and/or training programs, cognitive training, and management of vascular/metabolic risk factors. Intervention groups were compared to standard care, placebo, general information/health advice, or sham exercises.

Overall, the results appear to be mixed. Smaller (N<160 participants) and/or shorter trials (up to 24 weeks) seemed more likely to report intervention benefits on overall cognition and some specific domains (e.g., spatial working memory, executive functioning). Of the 5 larger (N>1000 participants) and longer-term trials (at least 2 years), only Finnish Geriatric Intervention Study to Prevent Cognitive Impairment and Disability (FINGER) reported significant intervention benefits on the primary and secondary cognitive outcomes [20]. Results from these 5 trials are difficult to compare directly due to substantial differences in e.g., target populations, format and intensity of the interventions, and outcome measures. However, several characteristics specific for the FINGER intervention model have been emphasized as potential reasons behind its cognitive benefits [21]: (i) selection of an at-risk older population (60-77 years) based on the validated Cardiovascular Risk Factors, Aging and Dementia (CAIDE) Risk Score [22]; (ii) multidomain intervention covering five domains, i.e. diet, exercise, cognitive training, social activities, and monitoring of vascular/metabolic

risk; and (iii) more intensive intervention, e.g. inclusion of an exercise program at the gym in addition to advice on physically active lifestyle, and inclusion of both individual and group sessions to ensure sufficient support and motivation for healthy lifestyle changes.

2.2. Risk stratification in multidomain intervention trials

A cursory look at the mixed findings shown in Table 1 may tempt clinicians into thinking that the multidomain intervention concept is not as promising as initially hypothesized. However, differences in design between larger, longer-term trials that met vs did not meet their primary outcomes suggest that multidomain intervention effectiveness may be highly dependent on a precision prevention approach, i.e., successfully identifying the at-risk groups who are most likely to benefit. To further investigate this, another literature search was conducted focusing on sub-group analyses assessing potential modifiers for the intervention effect on cognition in the multidomain prevention trials listed in Table 1. Identified sub-group analyses were based primarily on the FINGER, Multidomain Alzheimer Preventive Trial (MAPT) and Prevention of Dementia by Intensive Vascular Care (preDIVA) trials. Several of these analyses were pre-specified in the trial protocols, while others were conducted post-hoc. Results are summarized in Table 2.

In the FINGER trial, where participants were selected using the CAIDE Dementia Risk Score including age, sex, education, hypertension, hypercholesterolemia, obesity, and physical inactivity, the intervention seemed to be beneficial for cognition irrespective of further stratification by sociodemographic, cognitive or cardiovascular factors [33]. Although participants with a higher LIfestyle for BRAin health (LIBRA) index at baseline had overall less cognitive improvement over time, this effect was not different between intervention and control groups [25]. The LIBRA index is based on 12 modifiable risk factors [34], that partly overlap with those included in the CAIDE score, which may explain this result.

Interestingly, significant benefits on cognition were reported among participants in the MAPT trial with a CAIDE score ≥6 points (the same cut-off used in FINGER) [30]. Other analyses stratified by frailty status found no differences in intervention effect on cognition between frail and non-frail MAPT participants [28].

The LIBRA index did not identify high-risk individuals in whom the preDIVA intervention was beneficial [32]. However, preDIVA trial participants with untreated hypertension and who were adherent to the intervention had significantly lower risk of dementia compared with the control group

[31]. This is perhaps not surprising considering that the preDIVA intervention placed more weight on the cardiovascular risk management component compared with the lifestyle components. Participants without a history of cardiovascular disease who were adherent to the preDIVA intervention also had a significantly lower risk of dementia compared to control group.

The impact of genetic factors on the intervention effects on cognition was investigated only in the FINGER trial. No significant difference in intervention-related cognitive benefits was observed between *APOE* ε4 allele carriers vs non-carriers. However, analyses stratified by *APOE* ε4 carrier status showed a significant intervention-related cognitive benefit among the group of ε4 carriers [24]. In addition, a more pronounced cognitive benefit was reported in participants with shorter leukocyte telomere length at baseline, i.e. higher-risk individuals [26].

Brain imaging markers were also considered as potential intervention effect modifiers in the FINGER and MAPT trials. The MAPT intervention was reported to be associated with beneficial effects on cognition in individuals with amyloid positivity on positron emission tomography (PET) scans [29]. However, the FINGER intervention had more cognitive benefits in participants with higher brain volumes and cortical thickness at baseline [27]. It has been suggested that, while amyloid PET detects the early stages of amyloid deposition, morphological changes on MRI generally occur later in the Alzheimer's disease (AD) continuum [35]. In this context, the MAPT and FINGER findings emphasize that the best window of opportunity for precision risk reduction may be among individuals who have an increased dementia risk, but not yet substantial brain pathology and/or substantial cognitive/functional impairment. In other words, earlier and better targeted multidomain interventions may be most effective.

2.3. Estimating dementia risk reduction in early multidomain interventions

The AD continuum is characterized by a long period (up to decades) between the start of brain pathology and dementia onset [36]. In early interventions targeting at-risk individuals without substantial impairment, and with clinical trial durations that only very rarely exceed two-three years, dementia is not a feasible trial outcome. In the absence of direct data on the impact of multidomain interventions on reduction in dementia incidence, other ways to estimate risk reduction are needed. Multifactorial risk scores that provide standardized, evidence-based estimates for the risk of dementia may be particularly useful for this purpose and may also facilitate continuous monitoring of the intervention effects in practice by both clinicians and at-risk individuals.

Dementia risk scores have only recently started to be used in the context of prevention trials. For example, the FINGER trial used the CAIDE score for the recruitment of at-risk participants [20]. Several of the larger, longer-term multidomain intervention trials with cognition or dementia as primary outcomes are now also testing dementia risk scores as potential surrogate outcomes for estimating intervention effects on dementia risk reduction.

Table 3 summarizes dementia risk scores used as outcome measures in multidomain prevention trials, including those where cognitive performance or dementia are not the primary outcome. Two smaller and shorter-term trials with younger individuals, Body Brain Life [18] and the In-MINDD feasibility trial [37], have used a dementia risk score as the primary outcome. In the larger and longer-term trials, dementia risk scores have been used as outcomes in post-hoc analyses.

Overall, results indicate significant intervention benefits on the tested dementia risk scores, supporting the potential use of these scores for estimating dementia risk reduction. However, estimates from such analyses are currently difficult to interpret or compare between different risk scores, and would have to be verified against direct data on dementia incidence following the intervention. A potential solution for this could be extended follow-ups of trial participants after the intervention is completed, e.g., via healthcare registries if not otherwise feasible.

2.4. Dementia risk scores and brain pathology markers

Although many dementia risk scores have been developed for predicting subsequent dementia or cognitive decline, only two have so far been tested in relation to brain pathology (e.g. cerebrospinal fluid (CSF) or neuroimaging biomarkers, or brain pathology at autopsy). Detailed knowledge on the performance of a dementia risk score in predicting specific types of brain pathology (e.g., AD-related, or cerebrovascular) is essential to facilitate choosing the most suitable score for the purposes of a specific intervention, e.g., identification of at-risk individuals who are most likely to benefit, or monitoring of intervention effects on dementia risk reduction.

A summary of the reported relations between dementia risk scores and brain pathology markers is shown in Table 4. The CAIDE score is so far the most extensively tested in relation to biomarkers, including CSF and neuroimaging markers (structural MRI and amyloid PET), and post-mortem brain pathology. The Australian National University Alzheimer's Disease Risk Index (ANU-ADRI) score has been tested in relation to MRI markers.

Although neuropathology markers can be used directly as predictors of dementia risk, currently available markers (CSF and neuroimaging) are more difficult to assess outside highly specialized memory clinic settings, and their use is not always recommended in a population of cognitively unimpaired individuals for ethical or health economics reasons [47]. Validating simpler and easier to use dementia risk scores in relation to neuropathology markers would thus offer more cost-effective solutions for early identification of at-risk individuals in a broader range of clinical settings, where risk reduction interventions can also be started earlier, before the onset of substantial impairment requiring referral for more invasive and costly diagnostic procedures.

Another key aspect to consider when choosing a dementia risk score for precision risk reduction is to what extent it captures risk versus prevention potential, i.e., room for improvement with intervention. Risk scores such as CAIDE, ANU-ADRI or LIBRA include modifiable risk factors, thus indicating not only the risk profile, but also the intervention components that are needed to modify an individual's risk profile. It is currently unclear to what extent neuropathology markers could be used to estimate prevention potential, although they could be very useful as secondary outcomes in multidomain interventions that combine non-pharmacological approaches with disease-modifying drugs.

3. DEMENTIA VS CARDIOVASCULAR RISK REDUCTION

The 2019 WHO guidelines for risk reduction of cognitive decline and dementia also covered evidence on interventions targeted at reducing cardiovascular risk factors (e.g., hypertension, dyslipidemia, and diabetes) both pharmacologically and non-pharmacologically. The potential for integrating these recommendations into existing cardiovascular prevention programs was also emphasized. Although validated CVD risk scores have long been an established part of cardiovascular prevention, the testing of CVD risk scores in the context of dementia prevention has only recently started.

For example, the Framingham CVD risk score includes age, sex, systolic blood pressure, treatment for hypertension, HDL-cholesterol, total cholesterol, smoking, and diabetes. The Framingham stroke risk score combines age, systolic blood pressure, treatment for hypertension, diabetes, smoking, prior CVD (myocardial infarction, angina pectoris, coronary insufficiency, intermittent claudication, or congestive heart failure), atrial fibrillation, and left-ventricular hypertrophy. Both versions of the Framingham risk score at midlife have been reported to predict cognitive decline and dementia [48]. Additionally, the Framingham CVD risk score has been reported to predict vascular dementia [49] and clinical progression in patients with AD dementia, particularly in those with genetic and atherosclerotic risk factors [50]. However, the Framingham CVD risk score was not associated with structural brain measures on MRI [51].

The Framingham CVD risk score, and two dementia risk scores (CAIDE and Washington Heights-Inwood Columbia Aging Project, WHICAP) were investigated in relation to cognitive performance in different ethnic groups [52]. All three scores were significantly associated with cognition in both Hispanic/Latino and non-Hispanic/Latino populations.

Life's Simple 7 (LS7), defined by the American Heart Association as the 7 risk factors modifiable through lifestyle changes that can help achieve ideal cardiovascular health [53], has also been proposed as a potential tool for dementia risk reduction. The LS7 risk score includes four behavioral (smoking, diet, physical activity, body mass index) and three biological (fasting glucose, cholesterol, and blood pressure) factors. Lower LS7 score indicating poorer CVD health has been associated with higher risk of dementia in a long-term (25 years) observational study, while adherence to the LS7 ideal cardiovascular health recommendations in midlife has been linked to lower dementia risk [54]. Another CVD risk score including age, systolic blood pressure, total cholesterol, high-density lipoprotein, smoking, body mass index, and diabetes has been suggested as a useful tool for identifying individuals at risk for cognitive decline and dementia [55].

The global vascular risk score (GVRS) was developed to test whether the addition of behavioral and anthropometric risk factors to traditional vascular risk factors can improve prediction of clinical vascular events (e.g., stroke and myocardial infarction). The score combines age, sex, ethnicity, waist, alcohol consumption, smoking, physical activity, blood pressure, antihypertensive medication, peripheral vascular disease, blood glucose, and cholesterol. The GVRS has been associated with cognition, e.g. decline in global cognition, episodic memory, and processing speed over time, although this association seemed to be more pronounced in APOE ε4 non-carriers [56]. The GVRS has suggested as a feasible tool for use in primary care settings [57].

All abovementioned studies have been observational. So far only one study has investigated CVD risk scores in the context of clinical trials for dementia prevention, reporting that multidomain interventions designed for dementia risk reduction significantly improved CVD risk scores such as FINRISK and SCORE [39].

Although CVD risk scores seem promising as potential tools for dementia risk reduction, their testing and validation for this purpose is still far from the standards available in the field of cardiovascular prevention. An important issue is the longer- vs shorter-term prediction of dementia risk. Studies on dementia risk scores have clearly shown that risk profiles in midlife can be very different from risk profiles at older ages, and especially in older individuals who are already closer to dementia onset [58]. The time between onset of brain pathology and onset of clinical symptoms is also the time when "silent disease" can affect a variety of vascular, metabolic and lifestyle factors, i.e., reverse causality. This is the most likely reason why shorter-term observational studies (<5 years) in older populations often report associations between factors such as low blood pressure, low BMI, or low cholesterol and increased likelihood of dementia [58,59]. Such findings likely indicate markers on an ongoing dementia-related disease, and not actual risk factors. It is currently unclear if and to what extent CVD risk scores can be applied in older populations. Their associations with different types of brain pathology is also not yet determined.

4. EVIDENCE FROM A COMPLEX SINGLE-DOMAIN INTERVENTION

The new generation of non-pharmacological trials aiming to prevent cognitive decline and dementia includes not only multidomain intervention models, but also single-domain complex interventions. The LipiDiDiet trial included the highest level of single-domain intervention complexity, i.e. a medical food product including a multinutrient combination, Fortasyn Connect, composed of omega-3 fatty acids docosahexaenoic acid (DHA) and eicosapentaenoic acid (EPA), choline, uridine monophosphate, vitamins B6, B12, C, E, folic acid, phospholipids, and selenium [60]. The components of Fortasyn Connect have been studied to act as precursors and cofactors for neuronal membrane phospholipid synthesis [60] and are important to facilitate membrane formation which then impacts synaptic functioning and Aβ production. This type of medical food product has an interesting position between pharmacological and non-pharmacological interventions, due to potential disease-modifying effects. Impact on cognition has been studied in individuals with mild [61,62] and mild-to-moderate AD dementia [63]. Based on findings suggesting potential benefit in earlier, but not later disease stages, the LipiDiDiet trial tested Fortasyn Connect in individuals with prodromal AD in a multinational randomized controlled trial [64]. This was the first trial to use the International Working Group 1 (IWG-1) criteria for prodromal AD [65] to recruit 311 participants, shortly after the IWG-1 criteria were first published. The core trial was 2 years, followed by several optional 1-year extensions (in total 6 years, which is so far the longest completed prodromal AD trial). Both 24-month and 26-month results are now published, creating an interesting and promising overall picture.

In the 24-month core trial, the intervention did not have a significant effect on the primary cognitive outcome, but significant benefits were observed on key secondary outcomes such as change in Clinical Dementia Rating sum of boxes (CDR-SB) scores, and rate of hippocampal atrophy [64]. After 36 months, the benefits on CDR-SB and brain volumes were maintained and accentuated, and there were also evident benefits on the primary and other secondary cognitive outcomes [66]. Additional sub-group analyses by baseline MMSE score indicated benefits particularly in individuals with higher MMSE scores. Thus, this intervention seems to have the potential to alter disease trajectories, especially when started early, before substantial impairment has already occurred, and with longer-term treatment [66].

5. DISCUSSION

Dementia prevention is still relatively new compared with e.g., cardiovascular prevention, and much work is still left to be done to reach the standards of evidence and level of organization for pragmatic CVD risk reduction programs. Emerging evidence from recent multidomain prevention trials indicates that optimal preventive effects may be obtained through a precision risk reduction approach, i.e. targeting an individual's overall risk profile instead of separate risk factors, and tailoring the right interventions to the right people at the right time. Randomized controlled trials testing early dementia risk reduction interventions have an inherent design complexity that CVD trials do not have to deal with, particularly in terms of outcome definitions. While CVD outcomes targeted by preventive interventions tend to be acute, clearly identifiable events, this is not the case for outcomes related to dementia diseases that are chronic, slowly progressive, often insidious, and requiring more specialized assessments to detect (e.g., neuroimaging, CSF). In addition, it is not fully clear how much intervention exposure and in what format would be necessary for achieving optimal effects, or at least what minimal level of exposure would be needed for some benefit to still be derived from dementia risk reduction interventions. Moreover, since most of the multidomain interventions were conducted in high-income countries, it is not clear whether their results can be generalized to low- and middle-income countries and is therefore necessary to collect further evidence from different settings. Thus, longer-term randomized controlled trials are much needed to address these issues. One such example is World Wide-FINGERS (WW-FINGERS, currently about 35 member countries), the first global network for multimodal dementia prevention trials, where the FINGER intervention model is currently being tested, adapted and optimized in different populations, and geographic and economic settings, and focus is also on data harmonization and joint planning of these worldwide trials [67].

An important point regarding development and testing of dementia and/or CVD risk scores in the context of dementia risk reduction concerns how findings are reported in the literature. Standardized and transparent reporting is crucial to facilitate decision-making about the choice of the most suitable risk estimation tools for specific purposes. The TRIPOD statement (Transparent reporting of a multivariable prediction model for individual prognosis or diagnosis) [68] was published in 2015, and these guidelines would need to be followed similarly to e.g. CONSORT guidelines for reporting clinical trials, or STROBE guidelines for reporting cohort studies.

5.1. From research to implementation

Most risk reduction interventions have been conducted in a research setting. BHSs will allow to implement risk reduction interventions in the real world by offering the opportunity for cognitively unimpaired users to actively act and reduce their chances of developing dementia in the future. Before implementing risk reduction interventions, an accurate dementia risk profiling (assessing genetic, lifestyle and biological risk factors; Ranson et al., *this issue*) is needed to tailor the interventions to individual BHS users.

The 2019 WHO guidelines for risk reduction of cognitive decline and dementia [3] have emphasized that the implementation of interventions for cardiovascular and lifestyle risk factors may be combined with existing e.g. CVD or diabetes prevention programs and targeted to relevant populations. For this purpose, it is crucial that healthcare staff are fully aware of the importance of prevention in general and dementia prevention in particular. A recent survey highlighted that about 62% of the healthcare professionals did not consider dementia as a disorder but a condition of normal aging [69]. For effective implementation of prevention programs, a resource efficient way may be to combine dementia prevention with cardiovascular prevention which is substantially more advanced in knowledge, research, and implementation compared to the more recent field of dementia prevention. Also, shared risk factors between the two diseases can help the use of existing knowledge and services to advance the idea of dementia prevention from research to practice.

Engaging participants actively and in a meaningful manner is important in implementing prevention interventions. Large, longer-term multidomain intervention trials for dementia risk reduction have already shown that such interventions are feasible [11,14,31]. A first template for an operational model for dementia risk reduction has also been developed following the FINGER trial (Figure). Although several factors such as higher age, poorer cognition, depressive symptoms, and smoking have been reported to be associated with lower adherence to multidomain interventions, results vary across the trials and different intervention components [70,71]. Individually tailored approaches to risk reduction may also be more likely to ensure adherence. For example, a person at-risk may be compliant to a healthy diet but may need support with physical and cognitive activities, or another person with diabetes may need extra support for diet and management of other cardiovascular risk factors.

Initiating and maintaining healthy lifestyle changes in general is challenging at a personal level and is impacted by factors such as participants' knowledge, access to facilities, time management, preference, and attitude towards prevention. Another layer of complexity is added especially when considering implementation of such interventions or programs in low- and middle-income countries where prevention at mid-life may not be deemed as important as perceived in the Western world.

Rosenberg et al. 2020 [72] recently studied the reasons for participation in a European multinational, multidomain eHealth lifestyle prevention trial (HATICE) targeting at-risk older adults without significant cognitive impairment. The participants were asked to specify the reasons for participation in the trial to which most responded: the desire to contribute to scientific progress, the possibility to improve their own health through lifestyle changes, access to additional medical monitoring in the trial. Whether these same reasons motivate persons from other cultures and countries to participate and adhere to lifestyle interventions remains to be ascertained.

Therefore, it is important to identify motivating factors, participants' expectation and extending support to them or their active participation. Some motivating factors for participants to join and engage in prevention programs could be personal goal setting for the maintenance of participants' current and future health and avoidance of disability or dependency later in life [72]. Knowing their expectation during and after the participation would help educate them and gauge their goals and expectations realistically and for this those who are e.g., at higher risk or lagging in motivation, to offer them extra support.

5.2. Conclusion

Evidence on the efficacy of risk reduction interventions is promising, but not yet conclusive. More long-term multidomain randomized controlled trials are needed to fill the current evidence gaps, and the WW-FINGERS points in this direction. Nevertheless, consistent evidence suggests that a precision risk reduction approach may be most effective for dementia prevention. Such approach can be implemented in BHSs.

LIST OF ABBREVIATIONS

WHO: World Health Organization; CVD: cardiovascular conditions; BHS: Brain Health Services.

DECLARATIONS

Ethics approval and consent to participate: Not applicable.

Consent for publication: Not applicable.

Availability of data and materials: Data sharing is not applicable to this article as no datasets were generated or analyzed during the current study.

Competing interests

GBF reports grants from Alzheimer Forum Suisse, Académie Suisse des Sciences Médicales, Avid Radiopharmaceuticals, Biogen, GE International, Guerbert, Association Suisse pour la Recherche sur l'Alzheimer, IXICO, Merz Pharma, Nestlé, Novartis, Piramal, Roche, Siemens, Teva Pharmaceutical Industries, Vifor Pharma, and Alzheimer's Association; he has received personal fees from AstraZeneca, Avid Radiopharmaceuticals, Elan Pharmaceuticals, GE International, Lundbeck, Pfizer, and TauRx Therapeutics.

JLM is currently a full-time employee of Lundbeck and has previously served as a consultant or at advisory boards for the following for-profit companies, or has given lectures in symposia sponsored by the following for-profit companies: Roche Diagnostics, Genentech, Novartis, Lundbeck, Oryzon, Biogen, Lilly, Janssen, Green Valley, MSD, Eisai, Alector, BioCross, GE Healthcare, ProMIS Neurosciences.

PS has received consultancy fees (paid to the institution) from AC Immune, Alkermes, Alnylam, Anavex, Biogen, Brainstorm Cell, Cortexyme, Denali, EIP, ImmunoBrain Checkpoint, GemVax, Genentech, Green Valley, Novartis, Novo Noridisk, PeopleBio, Renew LLC, Roche. He is PI of studies with AC Immune, CogRx, FUJI-film/Toyama, IONIS, UCB, Vivoryon. He serves on the board of the Brain Research Center.

The other coauthors declare that they have no competing interests.

Funding

This paper was the product of a workshop funded by the Swiss National Science Foundation entitled "Dementia Prevention Services" (grant number: IZSEZ0_193593).

AS receives research funding from European Research Council grant 804371, Academy of Finland (287490, 294061, 319318), Yrjö Jahnsson Foundation, Finnish Cultural Foundation (Finland), Alzheimerfonden and Region Stockholm ALF (Sweden).

GBF received funding by: the EU-EFPIA Innovative Medicines Initiatives 2 Joint Undertaking (IMI 2 JU) "European Prevention of Alzheimer's Dementia consortium" (EPAD, grant agreement number: 115736) and "Amyloid Imaging to Prevent Alzheimer's Disease" (AMYPAD, grant agreement number: 115952); the Swiss National Science Foundation: "Brain connectivity and metacognition in persons with subjective cognitive decline (COSCODE): correlation with clinical features and in vivo neuropathology" (grant number: 320030_182772).

MK receives research funding from the Joint Programme - Neurodegenerative Disease Research (EURO-FINGERS), Academy of Finland (305810, 317465), Swedish Research Council, Center for Innovative Medicine (CIMED) at Karolinska Institutet, Region Stockholm (ALF, NSV), Knut and Alice Wallenberg Foundation, Stiftelsen Stockholms sjukhem, Konung Gustaf V:s och Drottning Victorias Frimurarstiftelse, Swedish Research Council for Health, Working Life and Welfare (FORTE).

Authors' contribution

Alina Solomon, Ruth Stephen, Philip Scheltens, and Miia Kivipelto conceptualized this Paper, drafted the manuscript for intellectual content, and approved the manuscript.

Emmanuel Carrera, Jenni Kulmala José Luis Molinuevo, Peter Nilsson, Tiia Ngandu, and Bruno Vellas revised the manuscript for intellectual content, and approved the manuscript.

Daniele Altomare, Giovanni B. Frisoni, and Federica Ribaldi conceived and organized the workshop whence the Papers of the BHS series in this issue of *Alzheimer's Research & Therapy* originated, conceived the related editorial initiative, revised this manuscript for intellectual content, harmonized the manuscript with the other Papers of the BHS series, and approved the manuscript.

Acknowledgement

European Task Force for Brain Health Services (in alphabetical order): Marc ABRAMOWICZ, Daniele ALTOMARE, Frederik BARKHOF, Marcelo BERTHIER, Melanie BIELER, Kaj BLENNOW, Carol BRAYNE, Andrea BRIOSCHI, Emmanuel CARRERA, Gael CHÉTELAT, Chantal CSAJKA, Jean-François DEMONET, Alessandra DODICH, Bruno DUBOIS, Giovanni B. FRISONI, Valentina GARIBOTTO, Jean GEORGES, Samia HURST, Frank JESSEN, Miia KIVIPELTO, David LLEWELLYN, Laura McWHIRTER, Richard MILNE, Carolina MINGUILLÓN, Carlo MINIUSSI, José Luis MOLINUEVO, Peter M NILSSON, Janice RANSON, Federica RIBALDI, Craig RITCHIE, Philip SCHELTENS, Alina SOLOMON, Cornelia VAN DUIJN, Wiesje VAN DER FLIER, Bruno VELLAS, Leonie VISSER.

REFERENCES

1. Frisoni GB, Molinuevo JL, Altomare D, Carrera E, Barkhof F, Berkhof J, et al. Precision prevention of Alzheimer's and other dementias: Anticipating future needs in the control of risk factors and implementation of disease-modifying therapies. Alzheimer's Dement [Internet]. John Wiley and Sons Inc; 2020 [cited 2020 Nov 24];16:1457–68. Available from: https://pubmed.ncbi.nlm.nih.gov/32815289/

2. Livingston G, Huntley J, Sommerlad A, Ames D, Ballard C, Banerjee S, et al. Dementia prevention, intervention, and care: 2020 report of the Lancet Commission [Internet]. Lancet. Lancet Publishing Group; 2020 [cited 2020 Sep 29]. p. 413–46. Available from: https://doi.org/10.1016/

3. World Health Organization. WHO | Risk reduction of cognitive decline and dementia. WHO. World Health Organization; 2019.

4. Hou X-H, Feng L, Zhang C, Cao X-P, Tan L, Yu J-T. Models for predicting risk of dementia: a systematic review. J Neurol Neurosurg Psychiatry. 2019;90:373.

5. Karmali KN, Persell SD, Perel P, Lloyd-Jones DM, Berendsen MA, Huffman MD. Risk scoring for the primary prevention of cardiovascular disease. Cochrane Database Syst Rev [Internet]. John Wiley & Sons, Ltd; 2017 [cited 2020 Nov 23]; Available from: http://doi.wiley.com/10.1002/14651858.CD006887.pub4

6. Barnes DE, Santos-Modesitt W, Poelke G, Kramer AF, Castro C, Middleton LE, et al. The mental activity and exercise (MAX) trial: A randomized controlled trial to enhance cognitive function in older adults. JAMA Intern Med [Internet]. JAMA Intern Med; 2013 [cited 2020 Nov 23];173:797–804. Available from: https://pubmed.ncbi.nlm.nih.gov/23545598/

7. Alves CRR, Merege Filho CAA, Benatti FB, Brucki S, Pereira RMR, de Sá Pinto AL, et al. Creatine Supplementation Associated or Not with Strength Training upon Emotional and Cognitive Measures in Older Women: A Randomized Double-Blind Study. Blachier F, editor. PLoS One [Internet]. Public Library of Science; 2013 [cited 2020 Dec 10];8:e76301. Available from: https://dx.plos.org/10.1371/journal.pone.0076301

8. Ihle-Hansen H, Thommessen B, Fagerland MW, Øksengård AR, Wyller TB, Engedal K, et al. Multifactorial vascular risk factor intervention to prevent cognitive impairment after stroke and TIA: A 12-month randomized controlled trial. Int J Stroke [Internet]. Blackwell Publishing Ltd; 2014 [cited 2020 Nov 23];9:932–8. Available from: https://pubmed.ncbi.nlm.nih.gov/23205666/

9. Fiatarone Singh MA, Gates N, Saigal N, Wilson GC, Meiklejohn J, Brodaty H, et al. The Study of Mental and Resistance Training (SMART) Study-Resistance Training and/or Cognitive Training in Mild Cognitive Impairment: A Randomized, Double-Blind, Double-Sham Controlled Trial. J Am Med Dir Assoc [Internet]. Elsevier Inc.; 2014 [cited 2020 Nov 23];15:873–80. Available from: https://pubmed.ncbi.nlm.nih.gov/25444575/

10. Lam LCW, Chan WC, Leung T, Fung AWT, Leung EMF. Would older adults with mild cognitive impairment adhere to and benefit from a structured lifestyle activity intervention to enhance cognition?: A cluster randomized controlled trial. PLoS One [Internet]. Public Library of Science; 2015 [cited 2020 Nov 23];10. Available from: /pmc/articles/PMC4380493/?report=abstract

11. Ngandu T, Lehtisalo J, Solomon A, Levälahti E, Ahtiluoto S, Antikainen R, et al. A 2 year multidomain intervention of diet, exercise, cognitive training, and vascular risk monitoring versus control to prevent cognitive decline in at-risk elderly people (FINGER): A randomised controlled trial. Lancet. Lancet Publishing Group; 2015;385:2255–63.

12. Matz K, Teuschl Y, Firlinger B, Dachenhausen A, Keindl M, Seyfang L, et al. Multidomain lifestyle interventions for the prevention of cognitive decline after ischemic stroke randomized trial. Stroke [Internet]. Lippincott Williams and Wilkins; 2015 [cited 2020 Sep 29];46:2874–80. Available from: https://pubmed.ncbi.nlm.nih.gov/26374482/

13. Van Dalen JW, Moll Van Charante EP, Caan MWA, Scheltens P, Majoie CBLM, Nederveen AJ, et al. Effect of Long-Term Vascular Care on Progression of Cerebrovascular Lesions. Stroke. Lippincott Williams and Wilkins; 2017;48:1842–8.

14. Andrieu S, Guyonnet S, Coley N, Cantet C, Bonnefoy M, Bordes S, et al. Effect of long-term omega 3 polyunsaturated fatty acid supplementation with or without multidomain intervention on cognitive function in elderly adults with memory complaints (MAPT): a randomised, placebo-controlled trial. Lancet Neurol. Lancet Publishing Group; 2017;16:377–89.

15. Espeland MA, Lipska K, Miller ME, Rushing J, Cohen RA, Verghese J, et al. Effects of Physical Activity Intervention on Physical and Cognitive Function in Sedentary Adults With and Without Diabetes. J Gerontol A Biol Sci Med Sci [Internet]. J Gerontol A Biol Sci Med Sci; 2017 [cited 2020 Sep 30];72:861–6. Available from: https://pubmed.ncbi.nlm.nih.gov/27590629/

16. Bae S, Lee S, Lee S, Jung S, Makino K, Harada K, et al. The effect of a multicomponent intervention to promote community activity on cognitive function in older adults with mild cognitive impairment: A randomized controlled trial. 2018; Available from: www.elsevier.com/locate/ctim

17. Blumenthal JA, Smith PJ, Mabe S, Hinderliter A, Lin PH, Liao L, et al. Lifestyle and neurocognition in older adults with cognitive impairments: A randomized trial. Neurology [Internet]. Lippincott Williams and Wilkins; 2019 [cited 2020 Dec 10];92:E212–23. Available from: https://n.neurology.org/content/92/3/e212

18. McMaster M, Kim S, Clare L, Torres SJ, Cherbuin N, D'Este C, et al. Lifestyle Risk Factors and Cognitive Outcomes from the Multidomain Dementia Risk Reduction Randomized Controlled Trial, Body Brain Life for Cognitive Decline (<scp>BBL-CD</scp>). J Am Geriatr Soc [Internet]. Blackwell Publishing Inc.; 2020 [cited 2020 Sep 29];jgs.16762. Available from: https://onlinelibrary.wiley.com/doi/10.1111/jgs.16762

19. Bischoff-Ferrari HA, Vellas B, Rizzoli R, Kressig RW, Da Silva JAP, Blauth M, et al. Effect of Vitamin D Supplementation, Omega-3 Fatty Acid Supplementation, or a Strength-Training Exercise Program on Clinical Outcomes in Older Adults: The DO-HEALTH Randomized Clinical Trial. JAMA - J Am Med Assoc [Internet]. American Medical Association; 2020 [cited 2020 Dec 9];324:1855–68. Available from: https://pubmed.ncbi.nlm.nih.gov/33170239/

20. Ngandu T, Lehtisalo J, Solomon A, Levälahti E, Ahtiluoto S, Antikainen R, et al. A 2 year multidomain intervention of diet, exercise, cognitive training, and vascular risk monitoring versus control to prevent cognitive decline in at-risk elderly people (FINGER): A randomised controlled trial. Lancet. 2015;385:2255–63.

21. Kulmala J, Ngandu T, Kivipelto M. Prevention Matters: Time for Global Action and Effective Implementation [Internet]. J. Alzheimer's Dis. IOS Press; 2018 [cited 2020 Nov 23]. p. S191–8. Available from: https://pubmed.ncbi.nlm.nih.gov/29504541/

22. Kivipelto M, Ngandu T, Laatikainen T, Winblad B, Soininen H, Tuomilehto J. Risk score for the prediction of dementia risk in 20 years among middle aged people: a longitudinal, population-based study. Lancet Neurol. Lancet Neurol; 2006;5:735–41.

23. Rosenberg A, Ngandu T, Rusanen M, Antikainen R, Backman L, Havulinna S, et al. Multidomain lifestyle intervention benefits a large elderly population at risk for cognitive decline and dementia regardless of baseline characteristics: The FINGER trial. Alzheimers Dement. Institute of Clinical Medicine, Department of Neurology, University of Eastern Finland, Kuopio, Finland. Electronic address: anna.rosenberg@uef.fi.; Department of Public Health Solutions, Chronic Disease Prevention Unit, National Institute for Health and : The Authors. Published by Elsevier Inc; 2018;14:263–70.

24. Solomon A, Turunen H, Ngandu T, Peltonen M, Levälahti E, Helisalmi S, et al. Effect of the apolipoprotein e genotype on cognitive change during a multidomain lifestyle intervention a subgroup analysis of a randomized clinical trial. JAMA Neurol [Internet]. American Medical Association; 2018 [cited 2020 Nov 23];75:462–70. Available from: https://pubmed.ncbi.nlm.nih.gov/29356827/

25. Deckers K, Koehler S, Ngandu T, Frans RJ V, Kivipelto M, Solomon A. Quantifying dementia prevention potential in the FINGER randomized controlled trial using the LIBRA prevention index. Alzheimer's Assoc Int Conf [Internet]. 2020;P4-[Posters: Public Health] Prevention (nonpharm. Available from: https://alz.confex.com/alz/20amsterdam/meetingapp.cgi/Paper/37948

26. Sindi S, Ngandu T, Hovatta I, K'areholt I, Antikainen R, Hänninen T, et al. Baseline telomere length and effects of a multidomain lifestyle intervention on cognition: The FINGER randomized controlled trial. J Alzheimer's Dis [Internet]. IOS Press; 2017 [cited 2020 Nov 23];59:1459–70. Available from: https://pubmed.ncbi.nlm.nih.gov/28777749/

27. Stephen R, Liu Y, Ngandu T, Antikainen R, Hulkkonen J, Koikkalainen J, et al. Brain volumes and cortical thickness on MRI in the Finnish Geriatric Intervention Study to Prevent Cognitive Impairment and Disability (FINGER). Alzheimer's Res Ther. BioMed Central Ltd.; 2019;11.

28. Tabue-Teguo M, Barreto de Souza P, Cantet C, Andrieu S, Simo N, Fougère B, et al. Effect of Multidomain Intervention, Omega-3 Polyunsaturated Fatty Acids Supplementation or their Combinaison on Cognitive Function in Non-Demented Older Adults According to Frail Status: Results from the MAPT Study. J Nutr Heal Aging [Internet]. Springer-Verlag France; 2018 [cited 2020 Nov 23];22:923–7. Available from: https://pubmed.ncbi.nlm.nih.gov/30272094/

29. Delrieu J, Payoux P, Carrié I, Cantet C, Weiner M, Vellas B, et al. Multidomain intervention and/or omega-3 in nondemented elderly subjects according to amyloid status. Alzheimer's Dement [Internet]. Elsevier Inc.; 2019 [cited 2020 Nov 23];15:1392–401. Available from: https://pubmed.ncbi.nlm.nih.gov/31558366/

30. Chhetri JK, de Souto Barreto P, Cantet C, Pothier K, Cesari M, Andrieu S, et al. Effects of a 3-Year Multi-Domain Intervention with or without Omega-3 Supplementation on Cognitive Functions in Older Subjects with Increased CAIDE Dementia Scores. J Alzheimers Dis. Department of Geriatrics, Gerontopole, CHU Toulouse, Purpan University Hospital, Toulouse, France.; Department of Geriatrics, Xuanwu Hospital, Capital Medical University, Beijing, China.; NSGG, Kathmandu, Nepal.; Department of Geriatrics, Gerontopole, CHU; 2018;64:71–8.

31. van Charante EPM, Richard E, Eurelings LS, van Dalen JW, Ligthart SA, van Bussel EF, et al. Effectiveness of a 6-year multidomain vascular care intervention to prevent dementia (preDIVA): a cluster-randomised controlled trial. Lancet. Lancet Publishing Group; 2016;388:797–805.

32. van Middelaar T, Hoevenaar-Blom MP, van Gool WA, Moll van Charante EP, van Dalen JW, Deckers K, et al. Modifiable dementia risk score to study heterogeneity in treatment effect of a dementia prevention trial: a post hoc analysis in the preDIVA trial using the LIBRA index. Alzheimers Res Ther. Department of Neurology, Academic Medical Center (AMC), Meibergdreef 9, 1105, AZ, Amsterdam, the Netherlands. t.vanmiddelaar@amc.uva.nl.; Department of Neurology, Donders Institute for Brain, Cognition and Behaviour, Radboud University Medical Center, Nij; 2018;10:62–4.

33. Kivipelto M, Solomon A, Ahtiluoto S, Ngandu T, Lehtisalo J, Antikainen R, et al. The Finnish Geriatric Intervention Study to Prevent Cognitive Impairment and Disability (FINGER): Study design and progress. Alzheimer's Dement. Alzheimers Dement; 2013;9:657–65.

34. Schiepers OJG, Kohler S, Deckers K, Irving K, O'Donnell CA, van den Akker M, et al. Lifestyle for Brain Health (LIBRA): a new model for dementia prevention. Int J Geriatr Psychiatry. School for Mental Health and Neuroscience, Alzheimer Center Limburg, Maastricht University, Maastricht, The Netherlands.; School for Mental Health and Neuroscience, Alzheimer Center Limburg, Maastricht University, Maastricht, The Netherlands.; School for : John Wiley & Sons, Ltd; 2018;33:167–75.

35. Dubois B, Hampel H, Feldman HH, Scheltens P, Aisen P, Andrieu S, et al. Preclinical Alzheimer's disease: Definition, natural history, and diagnostic criteria. Alzheimer's Dement. Elsevier Inc.; 2016. p. 292–323.

36. Dubois B, Feldman HH, Jacova C, Hampel H, Molinuevo JL, Blennow K, et al. Advancing research diagnostic criteria for Alzheimer's disease: The IWG-2 criteria. Lancet Neurol. Lancet Publishing Group; 2014. p. 614–29.

37. O'Donnell CA, Browne S, Pierce M, McConnachie A, Deckers K, van Boxtel MP, et al. Reducing dementia risk by targeting modifiable risk factors in mid-life: study protocol for the Innovative Midlife Intervention for Dementia Deterrence (In-MINDD) randomised controlled feasibility trial. Pilot feasibility Stud. General Practice and Primary Care, Institute of Health & Wellbeing, University of Glasgow, 1 Horselethill Road, Glasgow, G12 9LX Scotland UK.; General Practice and Primary Care, Institute of Health & Wellbeing, University of Glasgow, 1 Horselethill Road, ; 2015;1:40-015-0035-x. eCollection 2015.

38. Solomon A, Levälahti E, Antikainen R, Laatikainen T, Soininen H, Strandberg T, et al. Effects of a Multidomain Lifestyle Intervention on Overall Risk for Dementia: the Finger Randomized Controlled Trial. Alzheimer's Dement [Internet]. Elsevier; 2018;14:P1024–5. Available from: https://doi.org/10.1016/j.jalz.2018.06.2798

39. Barbera M, Ngandu T, Lehvälahti E, Coley N, Mangialasche F, Hoevenaar-Blom M, et al. Effect of multidomain interventions on estimated dementia and cardiovascular risk reduction: an individual-participant data meta-analysis from FINGER, MAPT, and Pre-DIVA. Alzheimer's Dement J Alzheimer's Assoc. 2020;

40. Coley N, Hoevenaar-Blom MP, van Dalen JW, Moll van Charante EP, Kivipelto M, Soininen H, et al. Dementia risk scores as surrogate outcomes for lifestyle-based multidomain prevention trials—rationale, preliminary evidence and challenges. Alzheimer's Dement [Internet]. John Wiley and Sons Inc.; 2020 [cited 2020 Sep 29]; Available from: https://pubmed.ncbi.nlm.nih.gov/32803862/

41. Vuorinen M, Spulber G, Damangir S, Niskanen E, Ngandu T, Soininen H, et al. Midlife CAIDE dementia risk score and dementia-related brain changes up to 30 years later on magnetic resonance imaging. J Alzheimer's Dis. IOS Press; 2015;44:93–101.

42. Enache D, Solomon A, Cavallin L, Kåreholt I, Kramberger MG, Aarsland D, et al. CAIDE Dementia Risk Score and biomarkers of neurodegeneration in memory clinic patients without dementia. Neurobiol Aging. Elsevier Inc.; 2016;42:124–31.

43. Stephen R, Liu Y, Ngandu T, Rinne JO, Kemppainen N, Parkkola R, et al. Associations of CAIDE Dementia Risk Score with MRI, PIB-PET measures, and cognition. J Alzheimer's Dis. IOS Press; 2017;59:695–705.

44. Hooshmand B, Polvikoski T, Kivipelto M, Tanskanen M, Myllykangas L, Mäkelä M, et al. CAIDE Dementia Risk Score, Alzheimer and cerebrovascular pathology: a population-based autopsy study. J Intern Med. John Wiley & Sons, Ltd (10.1111); 2018;283:597–603.

45. O'Brien JT, Firbank MJ, Ritchie K, Wells K, Williams GB, Ritchie CW, et al. Association between midlife dementia risk factors and longitudinal brain atrophy: the PREVENT-Dementia study. J Neurol Neurosurg Psychiatry. Department of Psychiatry, University of Cambridge, Cambridge, Cambridgeshire, UK.; Institute of Neuroscience and Newcastle University Institute for Ageing, Newcastle University, Newcastle upon Tyne, Tyne and Wear, UK michael.firbank@ncl.ac.uk.; Centre for: . No commercial re-use. See rights and permissions. Published by BMJ; 2019;

46. Cherbuin N, Shaw ME, Walsh E, Sachdev P, Anstey KJ. Validated Alzheimer's Disease Risk Index (ANU-ADRI) is associated with smaller volumes in the default mode network in the early 60s. Brain Imaging Behav. Springer New York LLC; 2019;13:65–74.

47. Johnson KA, Minoshima S, Bohnen NI, Donohoe KJ, Foster NL, Herscovitch P, et al. Appropriate use criteria for amyloid PET: a report of the Amyloid Imaging Task Force, the Society of Nuclear Medicine and Molecular Imaging, and the Alzheimer's Association. Alzheimers Dement [Internet]. Alzheimers Dement; 2013 [cited 2020 Dec 9];9. Available from: https://pubmed.ncbi.nlm.nih.gov/23360977/

48. Kaffashian S, Dugravot A, Elbaz A, Shipley MJ, Sabia S, Kivimäki M, et al. Predicting cognitive decline: A dementia risk score vs the framingham vascular risk scores. Neurology. Neurology; 2013;80:1300–6.

49. Li SS, Zheng J, Mei B, Wang HY, Zheng M, Zheng K. Correlation study of Framingham risk score and vascular dementia: An observational study. Med (United States) [Internet]. Lippincott Williams and Wilkins; 2017 [cited 2020 Nov 23];96. Available from: /pmc/articles/PMC5815664/?report=abstract

50. Viticchi G, Falsetti L, Buratti L, Boria C, Luzzi S, Bartolini M, et al. Framingham risk score can predict cognitive decline progression in Alzheimer's disease. Neurobiol Aging [Internet]. Elsevier Inc.; 2015 [cited 2020 Nov 23];36:2940–5. Available from: https://pubmed.ncbi.nlm.nih.gov/26279114/

51. Gourley D, Pasha EP, Kaur SS, Haley AP, Tanaka H. Association of Dementia and Vascular Risk Scores With Cortical Thickness and Cognition in Low-risk Middle-aged Adults. Alzheimer Dis Assoc Disord [Internet]. 2020;34. Available from: https://journals.lww.com/alzheimerjournal/Fulltext/2020/10000/Association_of_Dementia_and_Vascular_Risk_Scores.4.aspx

52. Torres S, Alexander A, O'Bryant S, Medina LD. Cognition and the Predictive Utility of Three Risk Scores in an Ethnically Diverse Sample. J Alzheimer's Dis [Internet]. IOS Press BV; 2020 [cited 2020 Nov 23];75:1049–59. Available from: https://pubmed.ncbi.nlm.nih.gov/32390625/

53. Lloyd-Jones DM, Hong Y, Labarthe D, Mozaffarian D, Appel LJ, Van Horn L, et al. Defining and setting national goals for cardiovascular health promotion and disease reduction: The american heart association's strategic impact goal through 2020 and beyond [Internet]. Circulation. Circulation; 2010 [cited 2020 Nov 23]. p. 586–613. Available from: https://pubmed.ncbi.nlm.nih.gov/20089546/

54. Sabia S, Fayosse A, Dumurgier J, Schnitzler A, Empana J-P, Ebmeier KP, et al. Association of ideal cardiovascular health at age 50 with incidence of dementia: 25 year follow-up of Whitehall II cohort study. BMJ [Internet]. 2019;366:l4414. Available from: http://www.bmj.com/content/366/bmj.l4414.abstract

55. Zeki Al Hazzouri A, Haan MN, Neuhaus JM, Pletcher M, Peralta CA, López L, et al. Cardiovascular risk score, cognitive decline, and dementia in older Mexican Americans: the role of sex and education. J Am Heart Assoc [Internet]. J Am Heart Assoc; 2013 [cited 2020 Nov 23];2. Available from: https://pubmed.ncbi.nlm.nih.gov/23608609/

56. Rundek T, Gardener H, Dias Saporta AS, Loewenstein DA, Duara R, Wright CB, et al. Global Vascular Risk Score and CAIDE Dementia Risk Score Predict Cognitive Function in the Northern Manhattan Study. J Alzheimers Dis [Internet]. NLM (Medline); 2020 [cited 2020 Nov 23];73:1221–31. Available from: https://pubmed.ncbi.nlm.nih.gov/31884476/

57. Tarraf W, Kaplan R, Daviglus M, Gallo LC, Schneiderman N, Penedo FJ, et al. Cardiovascular Risk and Cognitive Function in Middle-Aged and Older Hispanics/Latinos: Results from the Hispanic Community Health Study/Study of Latinos (HCHS/SOL). J Alzheimer's Dis [Internet]. IOS Press; 2020 [cited 2020 Nov 23];73:103–16. Available from: https://pubmed.ncbi.nlm.nih.gov/31771064/

58. Stephen R, Soininen H. Biomarker validation of a dementia risk prediction score. Nat Rev Neurol. 2020;

59. Duron E, Hanon O. Vascular risk factors, cognitve decline, and dementia [Internet]. Vasc. Health Risk Manag. Dove Press; 2008 [cited 2020 Nov 23]. p. 363–81. Available from: /pmc/articles/PMC2496986/?report=abstract

60. Van Wijk N, Broersen LM, De Wilde MC, Hageman RJJ, Groenendijk M, Sijben JWC, et al. Targeting synaptic dysfunction in Alzheimer's disease by administering a specific nutrient combination [Internet]. J. Alzheimer's Dis. IOS Press; 2014 [cited 2020 Nov 23]. p. 459–79. Available from: https://pubmed.ncbi.nlm.nih.gov/23985420/

61. Scheltens P, Twisk JWR, Blesa R, Scarpini E, Von Arnim CAF, Bongers A, et al. Efficacy of souvenaid in mild alzheimer's disease: Results from a randomized, controlled trial. J Alzheimer's Dis [Internet]. IOS Press; 2012 [cited 2020 Nov 23];31:225–36. Available from: https://pubmed.ncbi.nlm.nih.gov/22766770/

62. Scheltens P, Kamphuis PJGH, Verhey FRJ, Olde Rikkert MGM, Wurtman RJ, Wilkinson D, et al. Efficacy of a medical food in mild Alzheimer's disease: A randomized, controlled trial. Alzheimer's Dement [Internet]. Elsevier Inc.; 2010 [cited 2020 Nov 23];6. Available from: https://pubmed.ncbi.nlm.nih.gov/20129316/

63. Shah RC, Kamphuis PJ, Leurgans S, Swinkels SH, Sadowsky CH, Bongers A, et al. The S-Connect study: Results from a randomized, controlled trial of Souvenaid in mild-to-moderate Alzheimer's disease. Alzheimer's Res Ther [Internet]. BioMed Central; 2013 [cited 2020 Nov 23];5:59. Available from: /pmc/articles/PMC3978853/?report=abstract

64. Soininen H, Solomon A, Visser PJ, Hendrix SB, Blennow K, Kivipelto M, et al. 24-month intervention with a specific multinutrient in people with prodromal Alzheimer's disease (LipiDiDiet): a randomised, double-blind, controlled trial. Lancet Neurol. Lancet Publishing Group; 2017;16:965–75.

65. Dubois B, Feldman HH, Jacova C, DeKosky ST, Barberger-Gateau P, Cummings J, et al. Research criteria for the diagnosis of Alzheimer's disease: revising the NINCDS-ADRDA criteria. Lancet Neurol. Lancet Neurol; 2007. p. 734–46.

66. Soininen H, Solomon A, Visser PJ, Hendrix SB, Blennow K, Kivipelto M, et al. 36-month LipiDiDiet multinutrient clinical trial in prodromal Alzheimer's disease. Alzheimer's Dement [Internet]. John Wiley & Sons, Ltd; 2020;n/a. Available from: https://doi.org/10.1002/alz.12172

67. Kivipelto M, Mangialasche F, Snyder HM, Allegri R, Andrieu S, Arai H, et al. World-Wide FINGERS Network: A global approach to risk reduction and prevention of dementia. Alzheimer's Dement [Internet]. John Wiley and Sons Inc.; 2020 [cited 2020 Sep 29];16:1078–94. Available from: https://pubmed.ncbi.nlm.nih.gov/32627328/

68. Collins GS, Reitsma JB, Altman DG, Moons KGM. Transparent reporting of a multivariable prediction model for individual prognosis or diagnosis (TRIPOD): The TRIPOD statement. Ann Intern Med [Internet]. American College of Physicians; 2015 [cited 2020 Nov 23];162:55–63. Available from: https://pubmed.ncbi.nlm.nih.gov/25560714/

69. International D. World Alzheimer Report 2019: Attitudes to dementia; World Alzheimer Report 2019: Attitudes to dementia [Internet]. Available from: www.daviddesigns.co.uk

70. Beishuizen CRL, Coley N, van Charante EP, van Gool WA, Richard E, Andrieu S. Determinants of Dropout and Nonadherence in a Dementia Prevention Randomized Controlled Trial: The

Prevention of Dementia by Intensive Vascular Care Trial. J Am Geriatr Soc [Internet]. 2017;65:1505—1513. Available from: https://doi.org/10.1111/jgs.14834

71. Coley N, Ngandu T, Lehtisalo J, Soininen H, Vellas B, Richard E, et al. Adherence to multidomain interventions for dementia prevention: Data from the FINGER and MAPT trials. Alzheimer's Dement [Internet]. John Wiley & Sons, Ltd; 2019;15:729–41. Available from: https://doi.org/10.1016/j.jalz.2019.03.005

72. Rosenberg A, Coley N, Soulier A, Kulmala J, Soininen H, Andrieu S, et al. Experiences of dementia and attitude towards prevention: A qualitative study among older adults participating in a prevention trial. BMC Geriatr [Internet]. BioMed Central Ltd.; 2020 [cited 2020 Nov 23];20:99. Available from: https://bmcgeriatr.biomedcentral.com/articles/10.1186/s12877-020-1493-4

Box. Recommendations for practical implementation of precision dementia risk reduction interventions.

1. Target populations

- A risk reduction intervention should not be applied unselectively (focus on various at risk groups).
- At-risk groups should be preferably selected using validated risk scores or algorithms.
- The most suitable risk score or algorithm should be carefully chosen to fit the purpose, e.g., stage of the risk/disease continuum, age group, level of cognitive performance and type of intervention to be applied. For example, for multidomain lifestyle interventions the risk score/algorithm should select individuals with the type of risk profile that the intervention aims to modify.
- Risk reduction interventions should preferably start early, before substantial brain pathology and cognitive/functional impairment have already occurred.
- People with genetic susceptibility for dementia (e.g., based on *APOE* ε4 genotype) can also benefit from early risk reduction interventions.

2. Interventions

- Multidomain interventions (targeting several risk factors and disease mechanisms simultaneously) may be needed for an optimal dementia risk reduction.
- Interventions should (i) do the right things, and (ii) do enough of them, i.e., target an individual's overall risk profile with sufficient intensity to produce an effect. Only general healthy lifestyle advice may not be enough, and a more structured intervention program should be proposed.
- Intervention content should be adapted to local/national risk context (e.g., some risk factors may be more prevalent/severe in some countries than others) and various settings, and integrated with other chronic non-communicable diseases risk reduction programs when feasible.
- Radical lifestyle changes may be difficult to both initiate and maintain longer-term. Smaller changes gradually introduced across multiple lifestyle domains may facilitate long-term adherence.
- As the social component is important, group sessions and/or group activities should be facilitated when feasible.
- New technology may facilitate effective, personalized and feasible interventions and implementation (eHealth and mHealth).
- Intervention effects should be monitored. Risk scores could be useful for this purpose as well, if they include modifiable factors and are sufficiently sensitive to change over time.

Table 1. Overview of multidomain intervention trials for the prevention of cognitive decline and dementia.

**Study**	**Design and population**	**Multidomain intervention**	**Primary outcome**	**Main results**
The MAX trial (Barnes et al., 2013) [6]	N=126<br>Adults with cognitive complaints<br>Age: 65+ years<br>Duration: 12 weeks	Individual, home based mental activity plus class-based physical activity - 4 groups<br>1. Intervention (mental activity + exercise vs.<br>2. Intervention + Control (mental activity intervention + exercise control) vs.<br>3. Control + Intervention (mental activity control + exercise intervention) vs.<br>4. Control (mental activity + exercise)	Global cognitive change based on a comprehensive neuropsychological test battery	Physical plus mental activity was associated with significant improvements in global cognitive function.
Alves et al., 2013 [7]	N=56<br>Healthy women<br>Mean age: 66.8 years<br>Duration: 24 weeks	Creatine supplementation and exercise – 4 groups<br>1. Creatine supplementation vs.<br>2. Placebo vs.<br>3. Creatine supplementation + strength training vs.<br>4. Placebo + strength training	Cognitive function (memory, selective attention, and inhibitory control)	No significant effect on cognition.
Ihle-Hansen et al., 2014 [8]	N=195<br>Patients after first stroke<br>Mean age: 71.6 years<br>Duration:12 months	Outpatient stroke nurse and physician consultation 3- and 6-months post stroke, information about lifestyle and brain health. Medical treatment optimised. Tailored advice regarding risk factor management, treatment plan sent to general practitioner. Offered smoking cessation courses vs. Care as usual	Trail-making test A and 10-word test from baseline to 12 months follow-up	No difference between intervention and control groups
The SMART study (Fiatarone Singh et al., 2014) [9]	N= 100<br>Adults with MCI<br>Mean age: 70.1 years<br>Duration: 18 months	2 supervised interventions, 2-3 days/week for 6 months with 18 months follow-up<br>- Active OR sham physical training (high intensity progressive resistance training vs seated calisthenics)<br>plus<br>- Active OR sham cognitive training (computerized, multidomain cognitive training vs watching videos/quizzes)	Global cognitive function (ADAS-Cog) and functional independence	Resistance training significantly improved global cognitive function, with maintenance of executive and global benefits over 18 months
Lam et al., 2015 [10]	N= 555<br>Adults with MCI<br>Mean age: 75.4 years<br>Duration: 18 months	Physical exercise vs.<br>Cognitive activity vs.<br>Integrated cognitive and physical exercise vs.<br>Social activity (active control) groups	Clinical Dementia Rating sum of boxes (CDR-SOB) scores	No difference between groups for change in CDR-SOB and functional scores. Integrated physical and cognitive intervention exerted significantly better cognitive benefits on category verbal fluency test but not across all cognitive domains compared to single cognitive or physical activity intervention
FINGER<br>Ngandu et al., 2015 [11]	N=1260<br>Persons at-risk of dementia<br>Age: 60 to 77 years	Lifestyle intervention (diet, exercise, cognitive training, vascular risk monitoring) vs. General health advice	Cognition on neuropsychological test battery	Significant intervention benefit on cognition

	Duration: 2 years			
ASPIS (Matz et al., 2015) [12]	N=202<br>Stroke patients<br>Age: 40 to 80 years<br>Duration: 2 years	Multidomain intervention (clinical therapy, adequate blood pressure, lipid and glycaemic control, healthy diet, regular physical activity, cognitive training) vs. Standard stroke care	Cognition on Alzheimer Disease Assessment Scale & neuropsychological test battery	No difference between intervention and control groups.
Pre-DIVA (Moll van Charante et al., 2016) [13]	N= 3526<br>Community-dwelling older persons<br>Age: 70 to 78 years<br>Duration: 6 years	Multidomain intensive vascular care vs. Standard care	Incident dementia and disability score	No difference between intervention and control groups.
MAPT (Andrieu et al., 2017)[14][14]	N=1680<br>Community-dwelling older persons<br>Mean age: 75.3 years<br>Duration: 3 years	1. Multidomain intervention + omega-3 supplementation<br>2. Multidomain intervention + placebo<br>3. Omega-3 supplementation alone<br>4. Placebo alone	Cognitive decline on composite *Z* score	No difference between intervention and control groups.
Look AHEAD (Espeland et al., 2018) [15]	N=1091<br>Overweight or obese adults with type 2 diabetes<br>Age: 45 to 76 years<br>Duration: 10 years	Lifestyle intervention (diet modification and physical activity) yielding long-term weight loss vs. Support and education	Change in cognition (composite measure)	No difference between intervention and control groups
KENKOJISEICHI (Bae et al., 2019) [16]	N=83<br>Individuals with MCI<br>Mean age: 76 years<br>Duration: 24 weeks	Physical, cognitive, social activity sessions vs. Health education	Cognition on National Center for Geriatrics and Gerontology Functional Assessment Tool	Significant intervention effect on spatial working memory
Blumenthal et al., 2019 [17]	N=160<br>Older adults with cognitive impairment and no dementia<br>Mean age: >55 years<br>Duration: 6 months	Diet and exercise - 4 groups:<br>1. Aerobic exercise vs.<br>2. DASH diet nutritional counseling vs.<br>3. Combination of both aerobic exercise and DASH vs.<br>4. Health education	Global measure of executive cognitive functioning	Largest improvements were observed for combined aerobic exercise and DASH diet group
Body Brain Life for Cognitive Decline (McMaster et al., 2020) [18]	N=119<br>Subjective Cognitive Decline or Mild Cognitive Impairment<br>Age: 70 to 78 years<br>Duration: 8 weeks	Educational modules covering dementia and lifestyle risk factors, Mediterranean diet, physical activity, and cognitive engagement and additional active components: dietitian sessions, an exercise physiologist session, and online brain training vs. 4 online informational modules to reduce dementia risk	Dementia risk using Australian National University-Alzheimer's Disease Risk Index (ANU-ADRI) and cognition	Intervention group showed significantly lower ANU-ADRI score and a significantly higher cognition score than the control group
DO-HEALTH (Bischoff-Ferrari et al., 2020) [19]	N=2157<br>Adults having no major health events in the 5 years prior to enrolment, sufficient mobility, and good cognitive status<br>Age: 70 years or older<br>Duration: 3 years	Supplementation and exercise – 8 groups:<br>1. 2000 IU/d of vitamin D3, 1 g/d of omega-3s, and a strength-training exercise program vs.<br>2. Vitamin D3 and omega-3s vs.<br>3. Vitamin D3 and exercise vs.<br>4. Vitamin D3 alone vs.<br>5. Omega-3s and exercise vs.<br>6. Omega-3s alone vs.<br>7. Exercise alone vs.<br>8. Placebo	6 primary outcomes: Change in systolic and diastolic blood pressure, Short Physical Performance Battery (SPPB), Montreal Cognitive Assessment (MoCA), and incidence rates of nonvertebral fractures and infections	No statistically significant benefits of any intervention individually or in combination for all 6 end points

ADAS-Cog: Alzheimer's Disease Assessment Scale-Cognitive Subscale; ANU-ADRI: Australian National University Alzheimer's Disease Risk Index; ASPIS: Austrian Polyintervention Study to Prevent Cognitive Decline After Ischemic Stroke; CDR-SOB: Clinical Dementia Rating sum of

boxes; DASH: Dietary Approaches to Stop Hypertension; FINGER: Finnish Geriatric Intervention Study to Prevent Cognitive Impairment and Disability; MAPT: Multidomain Alzheimer Preventive Trial; MAX: The Mental Activity and eXercise; MoCA: Montreal Cognitive Assessment; PreDIVA: Prevention of Dementia by Intensive Vascular Care; SMART: Study of Mental and Resistance Training; SPPB: Short Physical Performance Battery.

Table 2. Examples of sub-group analyses assessing potential modifiers for the intervention effect on cognition in multidomain prevention trials.

Multidomain trials	Study	Potential intervention effect modifiers	Analyses	Results
FINGER	Rosenberg et al., 2018 [23]	Sex, age, and education, socioeconomic status, cognition, cardiovascular factors, and cardiovascular comorbidity at baseline	Pre-specified	No significant differences in cognitive intervention benefits by sex, age, and education, socioeconomic status, cognition, cardiovascular factors, and cardiovascular comorbidity
	Solomon et al., 2018 [24]	APOE ε4 allele	Pre-specified	Intervention benefits were not significantly different between carriers and noncarriers<br>Clear benefit in APOE4 carriers in stratified analyses
	Deckers et al., 2020 [25]	LIBRA index at baseline	Post-hoc	Participants with a higher LIBRA index at baseline had overall less cognitive improvement over time, but this effect was not different between intervention and control groups
	Sindi et al., 2017 [26]	Leukocyte telomere length	Post-hoc	More pronounced cognitive intervention benefits in individuals with shorter baseline leukocyte telomere length (higher-risk individuals)
	Stephen et al., 2019 [27]	Brain volumes and cortical thickness	Post-hoc	More pronounced cognitive intervention effects in individuals with higher brain baseline cortical thickness and volumes
MAPT	Tabue-Teguo et al., 2018 [28]	Frailty status	Post-hoc	Beneficial effects of multidomain intervention and n3 PUFA supplementation on cognition did not differ between frail and non-frail participants
	Delrieu et al., 2019 [29]	Amyloid status	Post-hoc	Multidomain intervention alone or in combination with omega-3 fatty acids was associated with improved primary cognitive outcome in individuals with positive amyloid status
	Chhetri et al., 2018 [30]	CAIDE score ≥6 points	Post-hoc	High-risk subjects for dementia screened with CAIDE dementia score might benefit more from multi-domain intervention
pre DIVA	Moll van Charante et al., 2016 [31]	Participants free from cardiovascular disease	Pre-specified	Participants with a history free from cardiovascular disease who were adherent to the intervention, had a significantly lower risk of dementia compared to control group
	Moll van Charante et al., 2016 [31]	Untreated hypertension at baseline	Pre-specified	Participants with untreated hypertension who were adherent to the intervention, had significantly lower risk of dementia compared with the control group
	van Middelaar et al., 2018 [32]	LIBRA index at baseline	Post-hoc	LIBRA modifiable dementia risk score did not identify a (high-)risk group in whom the multi-domain intervention was effective in preventing dementia or cognitive decline

Subgroup analysis type (prespecified and post-hoc) was assessed from published trial protocols.

APOE: Apolipoprotein E; CAIDE: Cardiovascular Risk Factors, Aging and Dementia; FINGER: Finnish Geriatric Intervention Study to Prevent Cognitive Impairment and Disability; LIBRA: LIfestyle for BRAin health; MAPT: Multidomain Alzheimer Preventive Trial; PreDIVA: Prevention of Dementia by Intensive Vascular Care; PUFA: Polyunsaturated fatty acids.

Table 3. Dementia risk scores used as surrogate outcomes in multidomain prevention trials.

Study	Trial	Dementia risk score	Outcome type	Main results
O'Donnell et al., 2015 [37]	In-MINDD	LIBRA	Primary	Participants in both arms of the trial showed a small improvement in their LIBRA score. The improvement was slightly larger in the intervention arm, but not statistically significant after 6 months
Solomon et al., 2018 [38]	FINGER	CAIDE	Post-hoc	Intervention had a significant impact on lowering the CAIDE risk score after 2 years
Barbera et al., 2020 [39]	FINGER<br>MAPT<br>preDIVA	CAIDE	Post-hoc,<br>Individual participants pooled analysis	CAIDE score decreased significantly as a result of the interventions after 2 years
Coley et al., 2020 [40]	preDIVA<br>MAPT<br>HATICE	LIBRA & CAIDE	Post-hoc,<br>each trial analysed separately	CAIDE and LIBRA scores showed statistically significant between-group differences after multidomain interventions after 1.5 to 2 years
Deckers et al., 2020 [25]	FINGER	LIBRA	Post-hoc	The intervention decreased dementia risk as indicated by decreasing LIBRA score after 2 years
McMaster et al., 2020 [18]	Body Brain Life	ANU-ADRI	Primary	Significant reduction in ANU-ADRI score for BBL compared with control after 2 months

ANU-ADRI: Australian National - University Alzheimer's Disease Risk Index; CAIDE: Cardiovascular Risk Factors, Aging and Dementia; FINGER: Finnish Geriatric Intervention Study to Prevent Cognitive Impairment and Disability; HATICE: Healthy ageing through internet counselling in the elderly; In-MINDD: Innovative Midlife Intervention for Dementia deterrence; LIBRA: LIfestyle for BRAin health; MAPT: Multidomain Alzheimer Preventive Trial; PreDIVA: Prevention of Dementia by Intensive Vascular Care.

Table 4. Dementia risk scores in relation to brain pathology markers.

Risk score	Study design	Biomarkers	Findings
**CAIDE**			
Vuorinen et al., 2015 [41]	Cohort, General population<br>N= 181<br>Mean age: 50 years<br>Follow-up: 30 years	Brain cortical thickness, white matter lesions, medial temporal atrophy on MRI	Higher score associated with higher medial temporal atrophy, white matter lesions and lower cortical thickness two to three decades later
Enache et al., 2016 [42]	Cohort, Memory clinic patients SCI and MCI<br>N= 724<br>Age: >40 years<br>Follow-up: Cross-sectional	AD-related CSF markers	Higher score associated with CSF markers of neurodegeneration (↓Aβ and ↑total tau)
Stephen et al., 2017 [43]	Cohort, at-risk for dementia<br>N= 132<br>Age: 60-77 years<br>Follow-up: 20-30 years	Brain volumes and cortical thickness, medial temporal atrophy, white matter lesions on MRI; and amyloid positivity on PiB-PET	Higher score associated with lower volumes and cortical thickness, medial temporal atrophy, white matter lesions but not with amyloid on PiB-PET
Hooshmand et al., 2018 [44]	Cohort, without dementia at baseline<br>N= 149<br>Age: ≥85 years<br>Follow-up: 10 years	Brain pathology at autopsy	Higher score associated with increased cerebral infractions
O'Brien et al., 2019 [45]	Cohort, middle-aged healthy adults<br>N= 149<br>Age: 40-59 years<br>Follow-up: 2 years	Rate of change in brain and ventricular volumes on MRI	Higher score associated with progressive brain atrophy rates
**ANU-ADRI**			
Cherbuin et al., 2019 [46]	Cohort, individuals free of dementia<br>N= 461<br>Age: 60-64 years<br>Follow-up: 12 years	Total and regional brain volumes on MRI	Higher score was associated with lower cortical gray matter particularly in the default mode network

Aβ: Amyloid-beta; AD: Alzheimer's disease; ANU-ADRI: Australian National University Alzheimer's Disease Risk Index.

CSF: cerebrospinal fluid; MRI: magnetic resonance imaging; PiB-PET: Pittsburgh compound B-positron emission tomography; MCI: mild cognitive impairment; SCI: subjective cognitive impairment.

Figure. FINGER operational model for dementia risk reduction.

FINGER operational model for dementia risk reduction

Start early. Identify at-risk people using the CAIDE Dementia Risk Score.

Identify each individual's personal risk factors.

Factors		Points
Age, years	< 47	0
	47-53	3
	>53	4
Education, years	≥10	0
	7-9	2
	0-6	3
Sex	Women	0
	Men	1
Systolic BP, mmHg	≤ 140	0
	> 140	2
BMI, kg/m^2	≤ 30	0
	> 30	2
Total cholesterol, mmol/l	≤ 6.5	0
	> 6.5	2
Physical activity	Active	0
	Inactive	1

If the score is ≥6 points, the person should be encouraged to participate in the multidomain intervention program.

Plan multidomain intervention program.

Different people may have different risk profiles, and it is important to consider individual needs and goals. Due to the multifactorial nature of dementia, several risk factors may need to be targeted simultaneously. The main FINGER intervention components are summarized below:

1. Strength training and aerobic exercise

Diverse physical activity should include both muscle training at the gym (1-3 times/week) and aerobic exercise (2-5 times/week). Training should be individually tailored and progressive. Exercises to improve postural balance should be included. It is important to select exercises that motivate and support mental wellbeing and social activity. Safety issues should be taken into account.

2. Healthy dietary habits

To support a brain healthy diet, it is important to recommend high consumption of fruits and vegetables, consumption of whole grain cereal products, and low-fat milk and meat products, limiting of sugar and salt intake, use of vegetable margarine instead of butter, and fish consumption at least 2 portions/week. Even small changes can make a difference.

3. Cognitive activity

Memory needs long-term, regular and challenging activity. Learning new things, engaging in hobbies that also support mental wellbeing and social activity are good for the brain. Listening to and playing music, learning new languages, reading and playing games are all cognitive stimulating activities.

4. Monitoring cardiovascular risk factors

Management of cardiovascular and metabolic risk factors should follow national evidence-based guidelines. Regular measurement of blood pressure, blood sugar and cholesterol levels are recommended.

Provide group activities to support a socially active lifestyle.

Provide motivation and support.

Maintain a positive attitude.

Highlight the benefits.

Support healthy lifestyle maintenance, follow-up the progression and evaluate the results of the intervention.

Personalized FINGER intervention model	
My risk score and date	
My plan to modify factors that increase my own dementia risk	
Why am I motivated to make these changes?	
When and where will my progress be followed?	

The model was first published in Finnish by the Finnish Institute for Health and Welfare (http://urn.fi/URN:NBN:fi-fe2018092136291).

Societal challenges for Brain Health Services.

A user manual for Brain Health Services – Part 6 of 6

Richard Milne, PhD[1,2]; Daniele Altomare, PhD[3,4]; Federica Ribaldi, MS[3,4,5,6]; José Luis Molinuevo, MD[7]; Giovanni B. Frisoni, MD[3,4]; Carol Brayne, MD[1] *on behalf of the European Task Force for Brain Health Services*

[1]Department of Public Health and Primary Care, University of Cambridge, Cambridge, UK.

[2] Society and Ethics Research Group, Wellcome Genome Campus, Hinxton, UK

[3]Laboratory of Neuroimaging of Aging (LANVIE), University of Geneva, Geneva, Switzerland.

[4]Memory Clinic, Geneva University Hospitals, Geneva, Switzerland.

[5]Laboratory of Alzheimer's Neuroimaging and Epidemiology (LANE), Saint John of God Clinical Research Centre, Brescia, Italy.

[6]Department of Molecular and Translational Medicine, University of Brescia, Brescia, Italy.

[7]Barcelonaβeta Brain Research Center, Pasqual Maragall Foundation, Barcelona, Spain.

Corresponding author

Richard Milne

University of Cambridge

Cambridge, UK

Email: rjm231@cam.ac.uk. Tel.: +44 (0)1223 496 836.

MANUSCRIPT DETAILS

Character count title (with spaces): 99.

Word count abstract: 162.

Word count manuscript: 2867.

Number of references: 46.

Number of tables: 1.

ABSTRACT

Brain Health Services are a novel approach to the personalized prevention of dementia. In this paper, we consider how such services can best reflect their social, cultural and economic context, and in doing so, deliver fair and equitable access to risk reduction. We present specific areas of challenge associated with the social context for dementia prevention. The first concentrates on how Brain Health Services engage with the 'at-risk' individual, recognizing the range of factors that shape an individual's risk of dementia and the efficacy of risk reduction measures. The second emphasizes the social context of Brain Health Services themselves, and their ability to provide equitable access to risk reduction. We then elaborate proposals for meeting or mitigating these challenges. We suggest that considering these challenges will enable Brain Health Services to address two fundamental questions: the balance between an individualized 'high-risk' and population focus for public health prevention, and the ability of services to meet ethical standards of justice and health equity.

Keywords

Brain health services; dementia; aging; Alzheimer's disease; prevention; public health; equity.

1. BACKGROUND

The development and implementation of 'precision' Brain Health Services (BHSs) represent a novel approach to reducing the risk of dementia in older adults. In this paper, we consider how such services can best reflect their social, cultural and economic context, and in doing so, deliver fair and equitable access to risk reduction. Our aim is to provide a constructive critique and initiate discussion around the challenges faced and raised by the brain health programs in its social context.

We focus on the model of the BHS as developed by Frisoni *et al.*.[1] This model sets out a model of personalized prevention, based around individualized multidomain interventions and educational activities and, in anticipation of potential disease-modifying therapies, around reshaped secondary care services. Whereas those with objective cognitive impairment are often served by existing memory clinics, BHSs extend to those without objective cognitive impairment but are concerned they are at higher risk of developing dementia for a potentially varying set of reasons, including family history. BHSs has four main missions (extensively discussed in the pertinent papers published in this issue of *Alzheimer's Research & Therapy*): dementia risk profiling (Ranson et al., *this issue*), dementia risk communication (Visser et al., *this issue*), dementia risk reduction (Solomon et al., *this issue*), and cognitive enhancement (Brioschi et al., *this issue*).

Our discussion proceeds as follows. First, we consider the practical challenges associated with the social and economic context for the delivery of BHSs. In each section we frame these around a specific challenge associated with the implementation of BHSs, and suggest potential practical, policy and research responses. In closing, we argue that situating BHSs in their social context requires attention to the ability of such services to deliver access to risk reduction in a socially just and equitable fashion.

In the following sections we present two specific areas of challenge associated with the social context for dementia prevention. The first concentrates on how BHSs engage with the 'at-risk' individual, recognizing the range of factors that shape an individual's risk of dementia and the efficacy of risk reduction measures. The second emphasizes the social context of BHSs themselves, and their ability to provide equitable access to risk reduction. Rather than simply pointing out challenges however, we also highlight proposals for remedying or mitigating them. In closing, we suggest that considering these challenges will enable BHSs to address two fundamental questions: the balance between a 'high-risk' and 'population' focus for public

health prevention, and the ability of services to meet ethical standards of justice and health equity.

2. THE AT-RISK INDIVIDUAL IN SOCIAL CONTEXT

2.1. The social determinants of dementia risk

- *Challenge*. A focus on self-referring high-risk individuals within specialist BHSs does not reflect the wider social and economic determinants of brain health, and cannot be the most effective and efficient approach to risk reduction at a population level.
- *Elaboration*. An individual's risk of developing dementia is a consequence of the accumulation of factors over the life course.[2,3] Globally, it is estimated that more than a third of the incidence of dementia in the population is due to exposure to potentially modifiable risk factors. The age-specific incidence of dementia is falling in many countries as a result of improvements in childhood education, nutrition, healthcare, and lifestyle changes.[4,5]

  These findings have clear implications for the promotion of brain health. The Lancet Commission focuses recommendations on 12 modifiable risk factors: the treatment of hypertension, childhood education, exercise, social engagement, smoking and alcohol consumption, hearing loss, depression, diabetes, obesity, air pollution and traumatic brain injury.[3] Many of these risk factors may be amenable to behavior change and lifestyle approaches. However, among them are those that are not easily incorporated into an approach to brain health focused on the individual rather than populations or groups – such as access to education in early life or exposure to air pollution. Further, even factors which are often considered as 'individually' modifiable are not equally distributed by ethnicity or gender, and are closely associated with poverty and inequality, unequally distributed across the social gradient and grounded in social and economic conditions that run through the life course.[3,6,7]

  The implication of these findings for the development of BHSs is two-fold. First, it suggests that primary prevention activities in populations have already had significant societal value and thinking how to develop these sensibly within contemporary populations is worthwhile. Second, it suggests that a 'high-risk' focus needs to engage with social and economic determinants of dementia risk, as this is where the greatest gain may be achieved in terms of future risk.
- *Solution*. Individualized approaches to brain health need to explicitly account for the social and economic distribution of risk across the life course in order to shift population risk

profiles for cognitive decline and dementia at each life stage.[8] All BHSs should commit to programs that are not only individual risk reduction programs, but that aim to empower individuals and communities, particularly those at increased disadvantage and therefore risk, to address the economic and social determinants of health and disease.

2.2. Risk communication and behavior change

- *Challenge*. The concept of BHSs relies largely on individualized risk prediction, using models derived from a combination of lifestyle, genetic and biomarker information. This information forms the basis for personalized 'precision' prevention plans. It is essential, however, that these interventions recognize the psycho-social challenges associated with behavior change.
- *Elaboration*. The use of genetic, biomarker and lifestyle information to identify 'high-risk' individuals for 'secondary prevention' is central to the BHS model. The use of this information to make significant differences to individual risk, however, remains understudied. Here, there is potential to learn from risk reduction programs in other clinical areas. The central message of systematic reviews of this work has been that the individualized provision of risk information alone has neither strong nor consistent effects on health-related behaviors.[9] In the case of cardiovascular disease, it has been shown that personalized risk information can improve the accuracy of an individual's perception of their risk, and may have implications for improving clinical prescribing, but again, has little effect on the way individuals live their lives.[10] In the case of genetic risk information, trials of the provision of genetic risk information have shown no or very limited effects on health behaviors.[11] For dementia itself, there is a need to be cognizant of the limitations of prediction models when applied to individuals, and the consequences of acting upon them given uncertainty in the evidence base that feed into these models.[12] This is particularly the case among those attending BHSs from the general population, rather than traditional memory clinic populations or among those with a diagnosis of mild cognitive impairment.[13–15]

  The sub-analyses of the FINGER and MAPT studies suggest that a multimodal programs of risk reduction may have some, limited in size, value for high-risk individuals, particularly those at greater genetic risk (i.e. *APOE* ε4 carriers)[16] or with brain amyloidosis. [17] However, these were not the primary endpoints and such findings must

be confirmed in targeted studies. Together, the current risk reduction interventions and the apparently limited value of approaches based solely on risk communication suggest there is an opportunity for work on dementia prevention to learn from, rather than repeat, the mistakes of other fields, and to recognize that to make a significant difference, BHSs should have a robust model of the complexity of health behaviors and their change.[18]

- *Solution*. Approaches to risk reduction that rely on individualized risk prediction leading to behavior changes are likely to be of limited effectiveness. Indeed, the vast bulk of evidence about changing behavior takes account of the fact most decision making is automatic, and that the way to change behavior is to change our environment to enable healthy behaviors. For dementia this means life-course environments that encourage physical activity, good nutrition, educational opportunities, social engagement, a health physical environment. The actual nature of the evidence base, rather than the hyperbole, for individualized risk reduction programs should be discussed with members of the public and patients who seek such approaches, in relation to their age, gender and ethnicity, with regards to short-, medium- and long- term prospects of benefit, known harms and likely cost.

3. THE BHSs IN A SOCIAL CONTEXT

3.1. Access to BHSs

- *Challenge*. Specialist BHSs risk compounding issues of access associated with existing clinical services, limiting their value to disadvantaged populations.
- *Elaboration*. Expanding services targeting the 'worried well' risk reinforcing the 'inverse care law', targeting those already well-served by health care and preventative health services, many of whom would probably have relatively little likelihood of benefitting and are probably most likely to have optimal brain health.[19] Where these services are reimbursed it is important to consider the trade-offs with services that are not being implemented, with better evidence bases that might achieve more for more people. [20] Where private health care providers offer services, potential customers who feel they may be beneficial should be informed about the state of evidence and its limitations. Indeed, emphasizing this point, the recent history of direct-to-consumer genetic testing and 'brain training' tools shows that there are likely to be many products and services whose marketing may lead individuals to assume a health benefit that is not justified by the evidence base.[21,22]

  In addition, interventions that require an individual to draw on their own resources – whether social, economic or psychological – tend to disproportionately benefit those with more of these resources.[23] This can result in the accumulation of disadvantage – both through exposure to risk, as discussed above, and in terms of access to testing and diagnostic services. Access to memory clinics, and even dementia services, is unequally distributed within and between countries, and is low around the world, varying significantly by country. In the UK and USA, access to a diagnosis has been shown to be associated with older age, male gender, and higher level of education and socio-economic status including income.[24–26] Those from less deprived socio-economic groups are more likely to be initiated on anti-dementia drugs than the most deprived, and may also present at clinic earlier in the progression of symptoms.[27,28] Ethnicity is also associated with access. In the USA, those from Hispanic populations are reported to be less likely to have received a diagnosis and to access diagnostic services later, and be less likely to access medication or research trials.[29] Similar findings have been found in the UK for Asian men and women, and Black men.[26,30,31] However, data on ethnicity is often not collected – in a study

based on a representative sample of UK primary care records, ethnicity was far more likely to be missing from records than information on deprivation.[30] This presents challenges in understanding who is accessing, and who is excluded, from BHSs.

Further, access to BHSs is likely to be shaped by socio-cultural framings of both dementia and risk. Available evidence suggests cultural factors that shape access to memory clinics, and, while specific evidence is lacking, it seems likely they would similarly affect the distribution of those who attend BHSs. Such cultural factors may differ by country and between ethnic groups. In the USA, for example, public expectations related to learning dementia risk reflect concerns about employment and insurance discrimination, considerations that, as Frisoni *et al.* rightly point out, impact on the decision to communicate risk status. Such considerations also shape how people access services.[32] In the UK, for example, qualitative research suggest hesitations associated with seeking a diagnosis among minority ethnic groups, and that medical help may only be sought for severe problems, with dementia seen as a private problem, associated with significant social stigma.[33–36]

To ensure that BHSs address and do not exacerbate inequalities in access, we need robust trial evidence before extending reimbursed BHSs to asymptomatic individuals. The overall benefit to societies of targeted early detection services has not yet been proven in clinical trials and the only international study of screening did not reveal any tangible benefits. It is important to acknowledge the existing clinical context for memory clinics that themselves were not developed out of an evidence base, but out of a need to identify individuals at an earlier stage of dementia or cognitive impairment from which to recruit to trials.

Here again, the development of the BHSs might usefully draw on the experience of other areas, such as the introduction of routine health checks in the field of cardiovascular disease prevention. In the UK, the introduction of general health checks has been evaluated as unlikely to be beneficial.[37] In fact, both health checks and individualized behavior change approaches may have potentially negative impacts on overall population health equity.[23,38] In contrast, evaluations of interventions in healthy eating suggest that those that focus ‘upstream’, for example by intervening in price, are likely to reduce inequalities.[39] The development of brain health programs should thus consider and evaluate the impact of such programs on health inequalities, and the potential for alternative approaches that may reduce inequity. This offers the opportunity to better understanding the impact of interventions across social inequalities, and to increase the potential for genuinely beneficial interventions that consider whole systems and the complex realities of

public health.[40] For example, modelling incorporating an explicit emphasis on equity suggests that targeting health checks to areas of disadvantage – using health care records to identify those at greatest risk of adverse outcomes – may make them more efficacious, beneficial and cost-effective.[38]

- *Solution*. To be of value, investment or reimbursement for individualized approaches to risk reduction must evaluate the value for those they represent in terms of long-term brain health, where/whether an individualized approach will lead to sufficient risk reduction to be systematically supported, and what the implications are in terms of equity. This includes the routine collection and evaluation of socio-economic data related to the impact of BHSs. It also involves the exploration of interventions whose ambition is to address systemic and population-level challenges.

4. ACHIEVING JUST BRAIN HEALTH

In the preceding sections, we have suggested that the challenges for BHSs, when considered in their social context, are considerable but not insurmountable. These challenges, and the remedies we have proposed, are summarized in Table 1. In closing, we consider the benefits of addressing them.

[Table 1 here]

The first relates to the balance between individualized 'high risk' and population prevention strategies. The former may have more significant impacts on individuals, whereas the latter may have more modest individual impacts but a greater impact across the population. Further, shifting the norm of behaviors across the population may have a subsequent impact on what is considered as 'high-risk' behavior. Thus, high-risk individuals may be more likely to take physical exercise or reduce smoking if this is considered to be normal.[41] As Frisoni *et al.* note, following Rose, high-risk and population strategies are not inherently exclusive.[1,42] The benefits of population prevention may sit alongside those targeting individuals with pathological changes associated with increased risk of dementia. However, as Rose also recognized, the complementarity of these approaches relies on a lack of competition for resources. In the current environment for healthcare where competition for resources is intense, a resource intensive high-risk approach inevitably limits the possibilities for population measures – even when the overall benefit of the latter may be greater.

The development of BHSs thus requires addressing a wider range of issues than currently considered within the 'ethics' of BHSs. Discussion of the ethics of dementia prevention has been dominated by a focus on autonomy, particularly the right of individuals to know, or not to know, their risk.[1,43] The challenges presented here emphasize the critical and compelling importance of widening this discussion, particularly as the concerns of the memory clinic and clinical ethics encounter those of population health, public health and societal ethics. It has been recognized that it is essential that efforts to prevent dementia "leave no one behind".[44] It is critical that social justice is embedded as a core value and guiding principle for brain health programs.[45,46] This means improving health to improve well-bring, by focusing on the needs of the most disadvantaged, with the aim of ensuring the fair distribution of common advantages

and the sharing of common burdens. At heart, following Beauchamp's framing of public health ethics, it requires thinking about and reacting to the problem of brain health as "primarily collective problems of the entire society".[46]

Conclusions

BHSs that focus on the delivery of personalized risk scores and interventions targeted at 'high risk' individuals may have potential as an approach limited to reducing risk in some sections of the population. To address risk requires this to be developed hand in glove with improving the means of reducing the overall population burden of dementia through life-course and broader societal measures. The latter are those that will change the incidence and prevalence of dementia, as well illustrated in the last 50 years. However, while high-risk and population approaches can be complementary, they risk competing for scarce resources, particularly given the resource-intensiveness of clinical risk assessment and follow-up. Without attention to the factors discussed here, an approach focusing primarily on high-risk populations is likely to struggle to deliver fair and equitable access to services or to risk reduction. It is therefore essential that those who invest in the development of BHSs aimed at individualized services consider the just allocation of resources.

LIST OF ABBREVIATIONS

BHS: Brain Health Service.

DECLARATIONS

Ethics approval and consent to participate: Not applicable.

Consent for publication: Not applicable.

Availability of data and materials: Data sharing is not applicable to this article as no datasets were generated or analyzed during the current study.

Competing interests

GBF reports grants from Alzheimer Forum Suisse, Académie Suisse des Sciences Médicales, Avid Radiopharmaceuticals, Biogen, GE International, Guerbert, Association Suisse pour la Recherche sur l'Alzheimer, IXICO, Merz Pharma, Nestlé, Novartis, Piramal, Roche, Siemens, Teva Pharmaceutical Industries, Vifor Pharma, and Alzheimer's Association; he has received personal fees from AstraZeneca, Avid Radiopharmaceuticals, Elan Pharmaceuticals, GE International, Lundbeck, Pfizer, and TauRx Therapeutics.

The other coauthors declare that they have no competing interests.

Funding

This paper was the product of a workshop funded by the Swiss National Science Foundation entitled "Dementia Prevention Services" (grant number: IZSEZ0_193593).

GBF received funding by: the EU-EFPIA Innovative Medicines Initiatives 2 Joint Undertaking (IMI 2 JU) "European Prevention of Alzheimer's Dementia consortium" (EPAD, grant agreement number: 115736) and "Amyloid Imaging to Prevent Alzheimer's Disease" (AMYPAD, grant agreement number: 115952); the Swiss National Science Foundation: "Brain connectivity and metacognition in persons with subjective cognitive decline (COSCODE): correlation with clinical features and in vivo neuropathology" (grant number: 320030_182772).

RM received funding by the EU-EFPIA Innovative Medicines Initiatives 2 Joint Undertaking (IMI 2 JU) "European Prevention of Alzheimer's Dementia consortium" (EPAD, grant agreement number: 115736).

Authors' contribution

Richard Milne and Carol Brayne conceptualized this Paper, drafted the manuscript for intellectual content, and approved the manuscript.

José Luis Molinuevo revised the manuscript for intellectual content, and approved the manuscript.

Daniele Altomare, Giovanni B. Frisoni, and Federica Ribaldi conceived and organized the workshop whence the Papers of the BHS series in this issue of *Alzheimer's Research & Therapy* originated, conceived the related editorial initiative, revised this manuscript for intellectual content, harmonized the manuscript with the other Papers of the BHS series, and approved the manuscript.

Acknowledgments

European Task Force for Brain Health Services (in alphabetical order): Marc ABRAMOWICZ, Daniele ALTOMARE, Frederik BARKHOF, Marcelo BERTHIER, Melanie BIELER, Kaj BLENNOW, Carol BRAYNE, Andrea BRIOSCHI, Emmanuel CARRERA, Gael CHÉTELAT, Chantal CSAJKA, Jean-François DEMONET, Alessandra DODICH, Bruno DUBOIS, Giovanni B. FRISONI, Valentina GARIBOTTO, Jean GEORGES, Samia HURST, Frank JESSEN, Miia KIVIPELTO, David LLEWELLYN, Laura McWHIRTER, Richard MILNE, Carolina MINGUILLÓN, Carlo MINIUSSI, José Luis MOLINUEVO, Peter M NILSSON, Janice RANSON, Federica RIBALDI, Craig RITCHIE, Philip SCHELTENS, Alina SOLOMON, Wiesje VAN DER FLIER, Cornelia VAN DUIJN, Bruno VELLAS, Leonie VISSER.

REFERENCES

1. Frisoni GB, Molinuevo JL, Altomare D, Carrera E, Barkhof F, Berkhof J, et al. Precision prevention of Alzheimer's and other dementias: Anticipating future needs in the control of risk factors and implementation of disease-modifying therapies. Alzheimer's & Dementia [Internet]. 2020 [cited 2020 Sep 22];n/a. Available from: https://alz-journals.onlinelibrary.wiley.com/doi/abs/10.1002/alz.12132

2. Norton S, Matthews FE, Barnes DE, Yaffe K, Brayne C. Potential for primary prevention of Alzheimer's disease: an analysis of population-based data. The Lancet Neurology. 2014;13:788–94.

3. Livingston G, Huntley J, Sommerlad A, Ames D, Ballard C, Banerjee S, et al. Dementia prevention, intervention, and care: 2020 report of the Lancet Commission. The Lancet [Internet]. Elsevier; 2020 [cited 2020 Jul 31];0. Available from: https://www.thelancet.com/journals/lancet/article/PIIS0140-6736(20)30367-6/abstract

4. Wu Y-T, Fratiglioni L, Matthews FE, Lobo A, Breteler MMB, Skoog I, et al. Dementia in western Europe: epidemiological evidence and implications for policy making. The Lancet Neurology. 2016;15:116–24.

5. Wolters FJ, Chibnik LB, Waziry R, Anderson R, Berr C, Beiser A, et al. 27-year time trends in dementia incidence in Europe and the US: the Alzheimer Cohorts Consortium. Neurology. 2020;

6. Marmot M, Allen J, Goldblatt P, Boyce T, McNeish D, Grady M. Fair society, healthy lives: the Marmot Review: strategic review of health inequalities in England post-2010. 2010. London: Department of International Development; 2019.

7. Russ TC, Stamatakis E, Hamer M, Starr JM, Kivimäki M, Batty GD. Socioeconomic status as a risk factor for dementia death: individual participant meta-analysis of 86 508 men and women from the UK. The British Journal of Psychiatry. Cambridge University Press; 2013;203:10–7.

8. Brayne C, Matthews FE. The determinants of cognitive decline and dementia. BMJ. 2019;l4946.

9. French DP, Cameron E, Benton JS, Deaton C, Harvie M. Can Communicating Personalised Disease Risk Promote Healthy Behaviour Change? A Systematic Review of Systematic Reviews. Ann Behav Med. Oxford Academic; 2017;51:718–29.

10. Usher-Smith JA, Silarova B, Schuit E, Moons KG, Griffin SJ. Impact of provision of cardiovascular disease risk estimates to healthcare professionals and patients: a systematic review. BMJ Open. British Medical Journal Publishing Group; 2015;5:e008717.

11. Hollands GJ, French DP, Griffin SJ, Prevost AT, Sutton S, King S, et al. The impact of communicating genetic risks of disease on risk-reducing health behaviour: systematic review with meta-analysis. BMJ. 2016;352:i1102.

12. Angehrn Z, Sostar J, Nordon C, Turner A, Gove D, Karcher H, et al. Ethical and Social Implications of Using Predictive Modeling for Alzheimer's Disease Prevention: A Systematic Literature Review. Journal of Alzheimer's Disease. IOS Press; 2020;76:923–40.

13. Stephan BCM, Kurth T, Matthews FE, Brayne C, Dufouil C. Dementia risk prediction in the population: are screening models accurate? Nature reviews Neurology. 2010;6:318–26.

14. Tang EYH, Harrison SL, Errington L, Gordon MF, Visser PJ, Novak G, et al. Current Developments in Dementia Risk Prediction Modelling: An Updated Systematic Review. PLOS ONE. Public Library of Science; 2015;10:e0136181.

15. van Maurik IS, Vos SJ, Bos I, Bouwman FH, Teunissen CE, Scheltens P, et al. Biomarker-based prognosis for people with mild cognitive impairment (ABIDE): a modelling study. The Lancet Neurology. 2019;18:1034–44.

16. Solomon A, Turunen H, Ngandu T, Peltonen M, Levälahti E, Helisalmi S, et al. Effect of the Apolipoprotein E Genotype on Cognitive Change During a Multidomain Lifestyle Intervention: A Subgroup Analysis of a Randomized Clinical Trial. JAMA Neurol. American Medical Association; 2018;75:462–70.

17. Andrieu S, Guyonnet S, Coley N, Cantet C, Bonnefoy M, Bordes S, et al. Effect of long-term omega 3 polyunsaturated fatty acid supplementation with or without multidomain intervention on cognitive function in elderly adults with memory complaints (MAPT): a randomised, placebo-controlled trial. Lancet Neurol. 2017;16:377–89.

18. Richard E, Moll van Charante EP, Hoevenaar-Blom MP, Coley N, Barbera M, van der Groep A, et al. Healthy ageing through internet counselling in the elderly (HATICE): a multinational, randomised controlled trial. The Lancet Digital Health. 2019;1:e424–34.

19. Hart JT. The Inverse Care Law. The Lancet. Elsevier; 1971;297:405–12.

20. Fowler NR, Perkins AJ, Gao S, Sachs GA, Boustani MA. Risks and Benefits of Screening for Dementia in Primary Care: The Indiana University Cognitive Health Outcomes Investigation of the Comparative Effectiveness of Dementia Screening (IU CHOICE)Trial. Journal of the American Geriatrics Society [Internet]. [cited 2019 Dec 18];n/a. Available from: https://onlinelibrary.wiley.com/doi/abs/10.1111/jgs.16247

21. Annas GJ, Elias S. 23andMe and the FDA. New England Journal of Medicine. Massachusetts Medical Society; 2014;370:985–8.

22. Schaper M, Schicktanz S. Medicine, market and communication: ethical considerations in regard to persuasive communication in direct-to-consumer genetic testing services. BMC Med Ethics. 2018;19:56.

23. Capewell S, Graham H. Will Cardiovascular Disease Prevention Widen Health Inequalities? PLOS Medicine. Public Library of Science; 2010;7:e1000320.

24. Savva GM, Arthur A. Who has undiagnosed dementia? A cross-sectional analysis of participants of the Aging, Demographics and Memory Study. Age Ageing. 2015;44:642–7.

25. Lang L, Clifford A, Wei L, Zhang D, Leung D, Augustine G, et al. Prevalence and determinants of undetected dementia in the community: a systematic literature review and a meta-analysis. BMJ Open. 2017;7:e011146.

26. Amjad H, Roth DL, Sheehan OC, Lyketsos CG, Wolff JL, Samus QM. Underdiagnosis of Dementia: an Observational Study of Patterns in Diagnosis and Awareness in US Older Adults. J Gen Intern Med. 2018;33:1131–8.

27. Cooper C, Lodwick R, Walters K, Raine R, Manthorpe J, Iliffe S, et al. Observational cohort study: deprivation and access to anti-dementia drugs in the UK. Age Ageing. 2016;45:148–54.

28. Qian W, Schweizer TA, Fischer CE. Impact of socioeconomic status on initial clinical presentation to a memory disorders clinic. International Psychogeriatrics. Cambridge University Press; 2014;26:597–603.

29. Cooper C, Tandy AR, Balamurali TBS, Livingston G. A systematic review and meta-analysis of ethnic differences in use of dementia treatment, care, and research. Am J Geriatr Psychiatry. 2010;18:193–203.

30. Pham TM, Petersen I, Walters K, Raine R, Manthorpe J, Mukadam N, et al. Trends in dementia diagnosis rates in UK ethnic groups: analysis of UK primary care data. Clin Epidemiol. 2018;10:949–60.

31. Ogliari G, Turner Z, Khalique J, Gordon AL, Gladman JRF, Chadborn NH. Ethnic disparity in access to the memory assessment service between South Asian and white British older adults in the United Kingdom: A cohort study. International Journal of Geriatric Psychiatry. 2020;35:507–15.

32. Stites SD, Milne R, Karlawish J. Advances in Alzheimer’s imaging are changing the experience of Alzheimer’s disease. Alzheimer’s and Dementia: Diagnosis, Assessment and Disease Monitoring. 2018;10.

33. Mukadam N, Cooper C, Livingston G. Improving access to dementia services for people from minority ethnic groups. Curr Opin Psychiatry. 2013;26:409–14.

34. Berwald S, Roche M, Adelman S, Mukadam N, Livingston G. Black African and Caribbean British Communities’ Perceptions of Memory Problems: “We Don’t Do Dementia.” PLoS One [Internet]. 2016 [cited 2020 Jul 17];11. Available from: https://www.ncbi.nlm.nih.gov/pmc/articles/PMC4821595/

35. Mukadam N, Cooper C, Basit B, Livingston G. Why do ethnic elders present later to UK dementia services? A qualitative study. Int Psychogeriatr. 2011;23:1070–7.

36. Stites SD, Rubright JD, Karlawish J. What features of stigma do the public most commonly attribute to Alzheimer’s disease dementia? Results of a survey of the U.S. general public. Alzheimer’s & Dementia. 2018;14:925–32.

37. Krogsbøll LT, Jørgensen KJ, Gøtzsche PC. General health checks in adults for reducing morbidity and mortality from disease. Cochrane Database of Systematic Reviews [Internet]. John Wiley & Sons, Ltd; 2019 [cited 2020 Jul 31]; Available from: https://www.cochranelibrary.com/cdsr/doi/10.1002/14651858.CD009009.pub3/full

38. Kypridemos C, Collins B, McHale P, Bromley H, Parvulescu P, Capewell S, et al. Future cost-effectiveness and equity of the NHS Health Check cardiovascular disease prevention programme: Microsimulation modelling using data from Liverpool, UK. PLOS Medicine. Public Library of Science; 2018;15:e1002573.

39. McGill R, Anwar E, Orton L, Bromley H, Lloyd-Williams F, O'Flaherty M, et al. Are interventions to promote healthy eating equally effective for all? Systematic review of socioeconomic inequalities in impact. BMC Public Health. 2015;15:457.

40. Rutter H, Savona N, Glonti K, Bibby J, Cummins S, Finegood DT, et al. The need for a complex systems model of evidence for public health. The Lancet. Elsevier; 2017;390:2602–4.

41. John SD. Risk, Contractualism, and Rose's "Prevention Paradox." Social Theory and Practice. Florida State University Department of Philosophy; 2014;40:28–50.

42. Rose G. Sick Individuals and Sick Populations. Int J Epidemiol. Oxford Academic; 1985;14:32–8.

43. Molinuevo JL, Cami J, Carné X, Carrillo MC, Georges J, Isaac MB, et al. Ethical challenges in preclinical Alzheimer's disease observational studies and trials: Results of the Barcelona summit. Alzheimer's & Dementia. 2016;12:614–22.

44. Prince M. Progress on dementia—leaving no one behind. The Lancet. Elsevier; 2017;390:e51–3.

45. Gostin LO, Powers M. What Does Social Justice Require For The Public's Health? Public Health Ethics And Policy Imperatives. Health Affairs. Health Affairs; 2006;25:1053–60.

46. Beauchamp DE. Public Health as Social Justice. Inquiry. Sage Publications, Inc.; 1976;13:3–14.

Table 1. The societal challenges encountered by the Brain Health Service model and accompanying recommendations.

**Challenge**	**Recommendation**
A focus on self-referring individuals does not reflect social determinants of brain health	Individualized approaches should explicitly account for social and economic distribution of risk<br><br>Brain Health Services should commit to wider work with communities to address social and economic determinants of health
Individualized risk reduction strategies face significant psycho-social barriers to implementation	Measures to reduce risk should recognize the importance of changing environments, rather than behaviors<br><br>The evidence for risk reduction should be discussed with individuals contemplating changing behaviors
Specialist Brain Health Services risk compounding inequalities in access to clinical services	Evidence is needed of the value of extending services to asymptomatic populations in terms of long-term effects on brain health<br><br>Brain health programs should consider and evaluate their impact on health inequalities
